# Trifluoromethylthiolation–arylation of diazocarbonyl compounds by modified Hooz multicomponent coupling†
†Electronic supplementary information (ESI) available: Experimental procedures, characterization and NMR spectra of the products. See DOI: 10.1039/c9sc00829b


**DOI:** 10.1039/c9sc00829b

**Published:** 2019-05-06

**Authors:** Marvin Lübcke, Dina Bezhan, Kálmán J. Szabó

**Affiliations:** a Department of Organic Chemistry , Arrhenius Laboratory , Stockholm University , SE-106 91 Stockholm , Sweden . Email: kalman.j.szabo@su.se

## Abstract

A new Zn-mediated trifluoromethylthiolation-based bifunctionalization reaction is developed. In this process, simultaneous C–SCF_3_ and C–C bond formation takes place in a multicomponent reaction, in which an aryl and a SCF_3_ group arise from different reagents. Our studies show that the reaction mechanism is similar to the Hooz multicomponent coupling. The process involves *in situ* generation of BAr_3_, which reacts with a diazocarbonyl compound, and the reaction is terminated by an electrophilic SCF_3_ transfer. The reaction can also be extended to fluorination based bifunctionalization which proceeds with somewhat lower yield than the analogous trifluoromethylthiolation reaction.

## Abstract 2

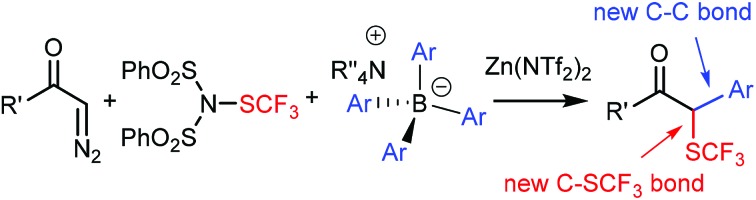
Multicomponent reaction of diazocarbonyl and dibenzenesulfonimide-SCF_3_ reagents with BAr_4_ salts in the presence of Zn(NTf_2_)_2_ gives α,α′-difunctionalized trifluoromethylthio compounds.

## 

The Hooz multicomponent reaction is based on coupling of diazocarbonyl compounds with organoboranes and electrophiles (Scheme 1).^1^ This and related reactions^2^ involve the formation of an adduct of the diazocarbonyl and the organoboron reagent followed by 1,2-migration of an alkyl substituent from the boron and being terminated by the reaction of an electrophile with the generated boron enolate (Scheme 1). The reaction is very useful for the synthesis of α,α′-bifunctionalized carbonyl compounds with formation of one or two new carbon–carbon bonds. As a part of our research program in organofluorine chemistry, we have developed several bifunctionalization methods^3^ based on the introduction of F/CF_3_/SCF_3_ groups. Recently, our interest^4^ turned to the synthesis of α,α′-(geminal)bifunctionalized species by using diazocarbonyl compounds1d,^5^ and electrophilic fluorine (F/CF_3_/SCF_3_) transfer reagents in multicomponent reactions.

**Scheme 1:**
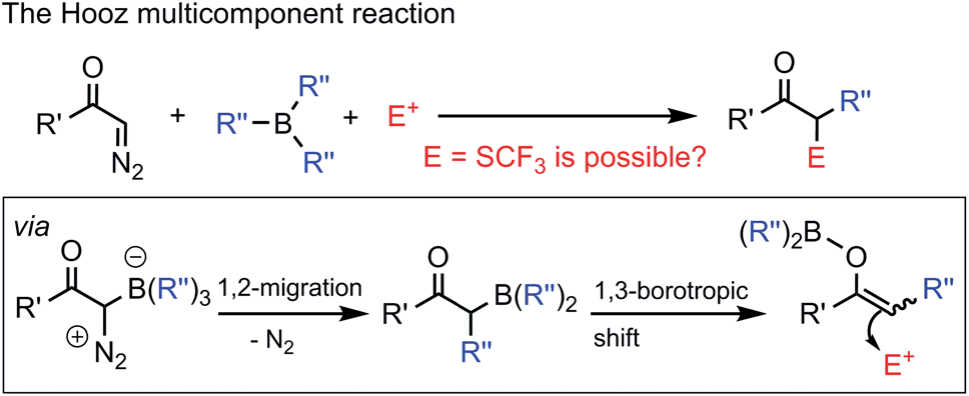
The Hooz multicomponent reaction of organoboranes and electrophiles E^+^.

Development of new methods for the synthesis of SCF_3_ compounds is particularly important, as functionalized trifluoromethylthiolates are attractive species in pharmaceutical industry, in crop protection and even in Positron Emission Tomography (PET) based medical diagnostics.^6^ The broad interest in synthesis of structurally diverse SCF_3_ compounds6a–c,^7^ is based on the favorable properties of the trifluoromethylthiolation group. Trifluoromethylthiolates can modify the binding properties and lipophilicity (Hansch parameter^8^*π* = 1.43) of bioactive small molecules. For instance, the activity of cephalosporin antibiotics can be substantially improved by installing a SCF_3_ functionality in cefazaflur,^9^ and tiflorex is a more efficient anorectic drug (Fig. 1) than its –CF_3_ analog.^10^

**Fig. 1:**
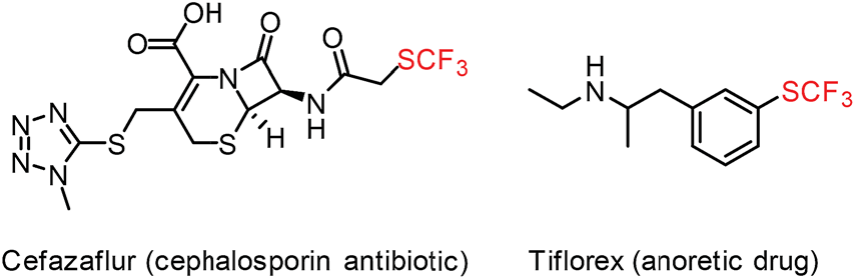
Examples of SCF_3_ containing drugs.

Many excellent methods have been reported recently for mono-trifluoromethylthiolation of organic substrates.6a–c,^7^,^11^ Our efforts have been focused on the development of trifluoromethylthiolation based bifunctionalization reactions to create structural complexity in a single multicomponent reaction. We have recently reported an efficient Rh-catalyzed procedure for geminal oxy-trifluoromethylthiolation of diazocarbonyl compounds (Scheme 2a).4*c* In the present study we aimed to develop a trifluoromethylthiolation based bifunctionalization reaction involving carbon–carbon coupling. There are relatively few reactions reported in the literature, in which bifunctionalization of diazoketones involve simultaneous C–SCF_3_ and C–C bond formation. A recent example is published by Wang and coworkers^12^ for the asymmetric trifluoromethylthiolation of sulfonium ylides *via* sigmatropic rearrangement (Scheme 2b), which is based on the Doyle–Kirmse^13^ reaction. Our idea was to develop a new multicomponent reaction, in which the SCF_3_ and aryl groups arise from different reagents. Considering the tremendous problems in functional group incompatibility in such multicomponent couplings, we hypothesized that the Hooz reaction (Scheme 1) could be a suitable platform for the realization of this transformation.

**Scheme 2:**
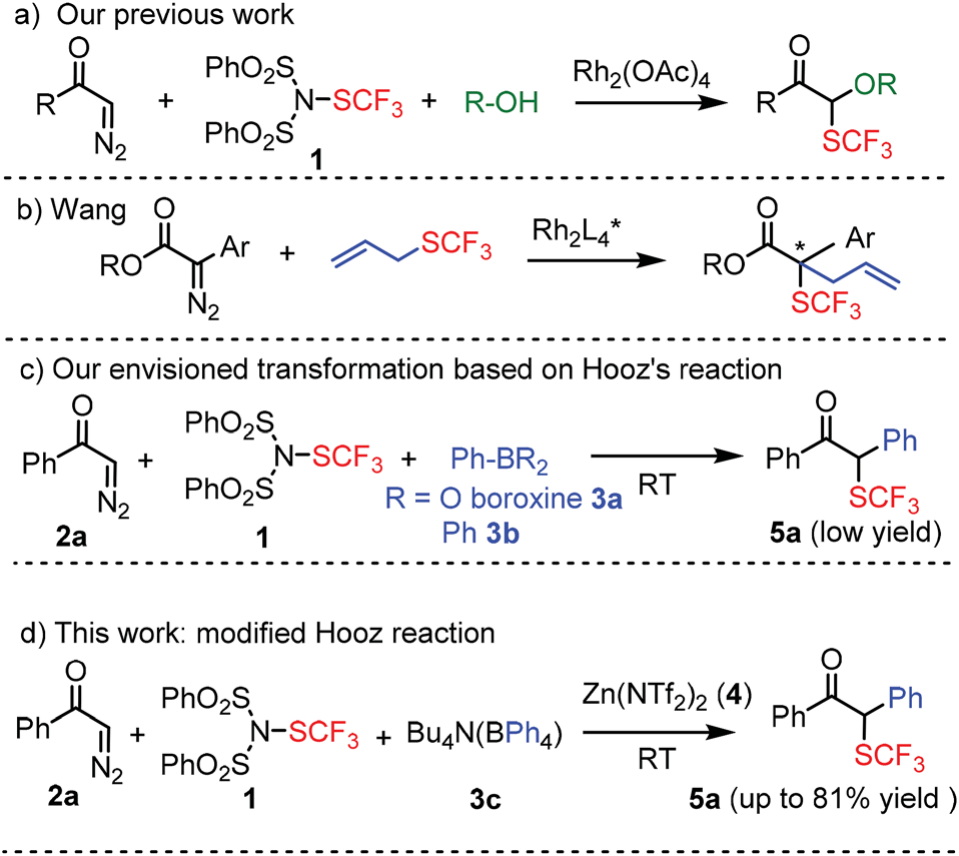
Trifluoromethylthiolation based bifunctionalization of diazocarbonyl compounds (a–d) including reactions with C–C and C–SCF_3_ coupling (b–d).

Our initial studies were performed under the typical conditions^1^ of Hooz multicomponent reactions (Scheme 2c). In this process, diazoketone **2a**, dibenzenesulfonimide^14^**1** (as electrophile) were reacted with various organoboron species (including phenylboronic acid/boroxine and BPh_3_). However, the reaction proceeded with low yield (up to 15%) even under strictly inert conditions (see below). When we modified the typical conditions of the Hooz reaction by application of tetraphenylborate derivatives (such as **3c**) and Zn(NTf_2_)_2_ (**4**), the SCF_3_/Ph-bifunctionalized product **5a** was obtained in high yield (Scheme 2d).

After careful optimization we found that the reaction proceeds in 81% yield when excess amounts of diazoketone **2a** (1.5 equiv.) and phenyl source **3c** (1.5 equiv.) were reacted with SCF_3_ transfer reagent **1** in the presence of (0.5 equiv.) Zn-salt (**4**) and molecular sieves at –10 °C (Table 1, entry 1). Deviations from these optimal conditions led to decreased yields or the formation of SCF_3_ product **5a** could not be observed. Reduction of the amount of Zn-mediator **4** (entry 2) led to a decrease of the yield of **5a** (37%) and without **4**, formation of **5a** was not observed at all (entry 3). As mentioned above, Rh_2_(OAc)_4_ was an excellent catalyst in the geminal oxy-trifluoromethylthiolation of diazocarbonyl compounds4*c* (Scheme 2a). However, phenyl-trifluoromethylthiolation of **2a** did not occur, when Zn-mediator **4** was replaced by a Rh-catalyst (entry 4).

**Table 1:** Deviation from the optimal reaction conditions for the α,α′-trifluoromethylthiolation–phenylation of diazoketone **2a**^*a*^

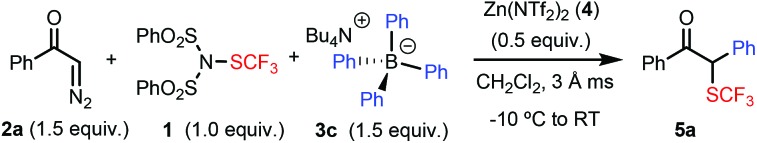		
Entry	Deviation from the standard conditions	Yield^*b*^ (%)
1	None	81
2	0.25 equiv. Zn(NTf_2_)_2_ (**4**)	37
3	Without Zn(NTf_2_)_2_ (**4**)	<5
4	Rh_2_(OAc)_4_ (5 mol%) instead of **4**	0
5	Pd(OAc)_2_ (15 mol%) instead of **4**	30
6	Zn(OTf)_2_ instead of **4**	<5
7	0.5 equiv. of **3c**	28
8	Na(BPh_4_) instead of **3c**	52
9	(PhBO)_3_ (**3a**) instead of **3c**, without **4**	0
10	BPh_3_ (**3b**) instead of **3c**, without **4**	15
11	ZnPh_2_ instead of **3c**	<5
12	ZnPh_2_ instead of **4** and BPh_3_**3b** instead of **3c**	0
13	PhMe as the solvent	71
14	THF as the solvent	35
15	MeCN as the solvent	0
16	Without 3Å ms	59
17	22 °C instead of –10 °C	66

^*a*^To reagent **1** (0.1 mmol), Bu_4_N(BPh_4_) (**3c**) (0.15 mmol), Zn(NTf_2_)_2_ (**4**) (0.05 mmol) and 80 mg 3Å molecular sieves (ms) was added a solution of diazoketone **2a** (0.15 mmol) in CH_2_Cl_2_ (1.0 ml) at –10 °C. This mixture was stirred at –10 °C for 2 h before allowing it to warm up to RT overnight.

^*b*^Isolated yield.

When Pd(OAc)_2_ was used instead of **4**, a complex reaction mixture was obtained, from which **5a** could be isolated in 30% yield (entry 5). According to the analysis of the crude reaction mixture by ^19^F NMR, in this process a large amount (up to 35%) of Ph–SCF_3_ was formed indicating that two components (**1** and **3c**) of the three-component reaction may react directly in a Pd-catalyzed process. Other Zn-salts in place of **4**, such as Zn(OTf)_2_, were not able to mediate the reaction (entry 6). Using Na(BPh_4_) instead of Bu_4_N(BPh_4_) **3c** led to a decrease of the yield (52%) probably because of its poor solubility in DCM (entry 8). This gave the idea to study various solubilizing reagents together with Na(BPh_4_) (see below). As mentioned above, phenylboroxine **3a** was inefficient as phenyl source (entry 9) and application of BPh_3_ (**3b**) (typical Hooz conditions) instead of **3c**/**4** led to a poor yield (15%) of **5a** (entry 10). A boron-based phenyl source is important for the bifunctionalization reaction, as ZnPh_2_ instead of **3c** gave no product **5a** (entry 11). The combination of BPh_3_ (**3b**) and phenylzinc reagent ZnPh_2_ did not result in product **5a** formation, indicating that phenyl transfer cannot happen from an external phenylzinc source in the presence of BPh_3_ (entry 12). We briefly screened the solvent effects as well. The reaction proceeds with good yield (71%) in toluene (entry 13) but in more polar solvent, such as in THF (entry 14) the yield is lower (35%) and we did not observe any formation of **5a** in acetonitrile (entry 15). The dry conditions are apparently important for the high yield of **5a**, as without molecular sieves the yield dropped to 59% (entry 16). At room temperature instead of –10 °C, the yield was decreased to 66% (entry 17).

As mentioned above, the reactions proceeded with high yield with Bu_4_N(BPh_4_) **3c**, which is soluble in DCM, while the yield dropped, when sparingly soluble Na(BPh_4_) was employed (*c.f.* entries 1 and 8). Therefore, we attempted to increase the yield (52%) of the reaction with Na(BPh_4_) using phase transfer (PT) catalysts (Table 2). Using Bu_4_N(BPh_4_) **3c** and Bu_4_N(NTf_2_) in 10 mol% as PT catalyst (Table 2 entries 2 and 3) increased the yield to 66% and 64%, respectively. However, the yields with Na(BPh_4_) in the presence of PT catalysts were still lower than with Bu_4_N(BPh_4_) **3c** as the phenyl source.

**Table 2:** Phase transfer experiments for the 1,1-trifluoromethyl-thiolation-phenylation^*a*^

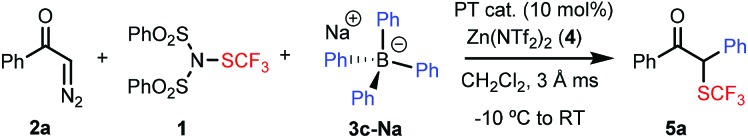		
Entry	Phase transfer (PT) catalyst	Yield^*b*^ of **5a** (%)
1	None	52
2	Bu_4_N(BPh_4_)	66
3	Bu_4_N(NTf_2_)	64

^*a*^Standard conditions according to Table 1, entry 1.

^*b*^Isolated yield.

With the optimized reaction conditions in hand we investigated the substrate scope of this reaction by varying the diazocarbonyl and the organoboron reagents. Diazoketones bearing halogen or EWD nitro substituents (Table 3, entries 2–4) on the aryl ring (**2b–d**) reacted with high yields (73–84%), similarly to phenyl derivative **2a** (entry 1), affording the corresponding SCF_3_ derivatives **5b–d**. The aromatic iodo substituent in **2c** (entry 3) remained unchanged under the reaction affording **5c**, which has a useful handle for subsequent Pd-catalyzed coupling reactions. With the presence of an electron donating methoxy group (**2e**) in the substrate, the yield (60%) was somewhat lower than for the phenyl derivative **5a**. Aryldiazoketone **2f** containing a similar tetrazole motif as cefazaflur (Fig. 1) reacted smoothly providing **5f** in 68% yield (entry 6). Due to the low solubility of **2f** in DCM, this reaction was conducted at room temperature instead of –10 °C. Furane based diazoketone **2g** reacted with high yield (84%) affording the SCF_3_ product **5g** (entry 7). Disubstituted diazoketone **2h** also underwent the phenylation–trifluoromethylthiolation reaction affording **5h**, in which the phenyl and SCF_3_ groups are attached to a quaternary carbon (entry 8). This reaction occurred with lower yield (30%) compared to the formation of the tertiary substituted trifluoromethylthio derivatives, such as **5a**, probably because of steric reasons (see below). Not only aromatic but even aliphatic diazoketones (**2i–j**) could be used as substrates (entries 9 and 10). The successful bifunctionalization of **2i–j** could be due to the mild reaction conditions without added base leaving the α-keto hydrogens unchanged. In particular, nonyl derivative **2i** reacted with high yield (83%), while the bifunctionalization of cyclopentyl derivative **2j** occurred with a lower yield (48%). Unlike in the Rh-catalyzed oxy-trifluoromethylthiolation reaction,4*c* diazoester **2k** underwent the phenylation–trifluoromethylthiolation reaction smoothly (entry 11) affording **5k** in good yield (78%). This result, together with the above mentioned observation (Table 1, entry 4), shows that the trifluoromethylthiolation based bifunctionalization of diazocarbonyl compounds in Rh-catalyzed and in Hooz-type reactions proceed with substantially different mechanisms (see below). A ten-fold scale up of the reaction of diazoester **2k** with **1** and **3c** (entry 11) could be performed without significant change of the yield (74%). When diazoacetamide **2m** was employed, bifunctionalized product **5m** was obtained in high yield (entry 12). However, diazomalonate **2n** could not be converted to the corresponding product **5n** (entry 13), in which the phenyl and SCF_3_ groups would be attached to a quaternary carbon. We have briefly studied the possibilities for a phenylation–fluorination reaction (entry 14) using NFSI instead of **1** as a fluorine-electrophile source with **2d** as the substrate. This reaction occurred with a lower yield (55%) than the analogous process with **1** (entry 4, 84%). In addition, fluoro derivative **6** and (4-nitrophenyl)-2-phenylethan-1-one were formed in a 1 : 1 mixture, indicating that protonation of the key reaction intermediate is about as fast as fluorination. The observation that substantial amounts of protonation product formed in the fluorination (entry 12) compared to the trifluoromethylthiolation (entry 4) reaction under similar conditions confirms our previous conclusion^15^ that **1** is a more efficient electrophile than NFSI in bifunctionalization reactions (see also the control experiments below). Due to their similar polarity, the separation of the mixture of the fluorinated and protonated products was cumbersome, which somewhat decreases the synthetic utility of this phenylation–fluorination-based bifunctionalization of diazoketones. The yield (55%) of **6** was determined after chromatography using a sample, which was contaminated with the protonated analog [1-(4-nitrophenyl)-2-phenylethan-1-one]. However, **6** could be further purified by selective oxidation of the protonated byproduct (see ESI†).

**Table 3:** 1,1-Trifluoromethylthiolation–phenylation of diazo compounds **2** with SCF_3_-source **1** and Bu_4_N(BPh)_4_ (**3a**)^*a*^

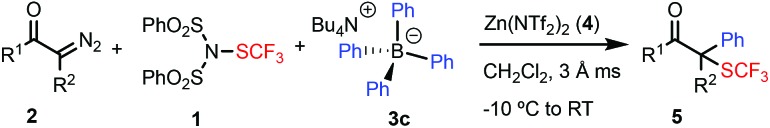			
Entry	Diazocarbonyl compound **2**	Carbonyl compound **5**	Yield^*b*^ of **5** (%)
1	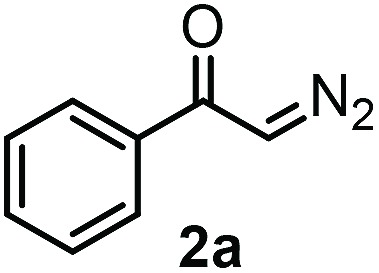	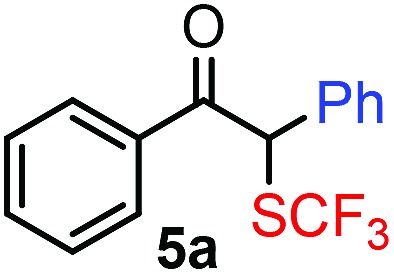	81
2	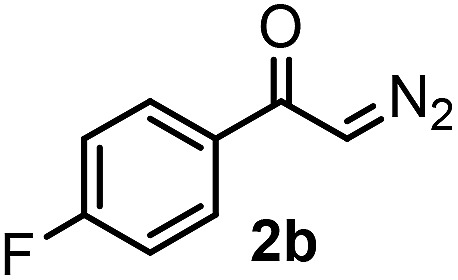	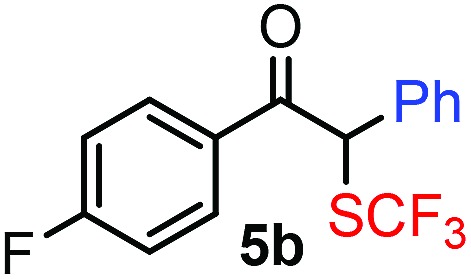	80
3	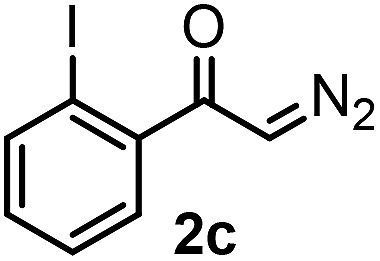	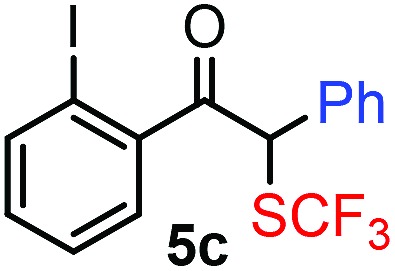	73
4	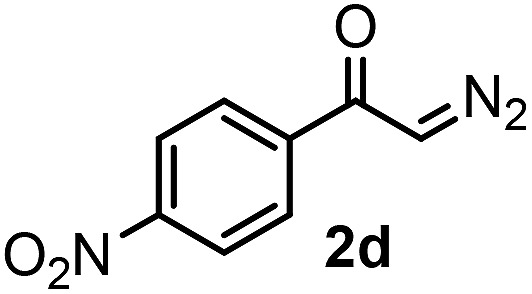	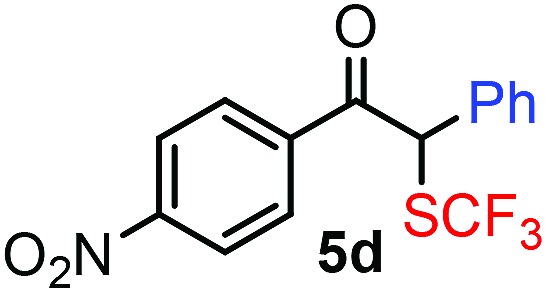	84
5	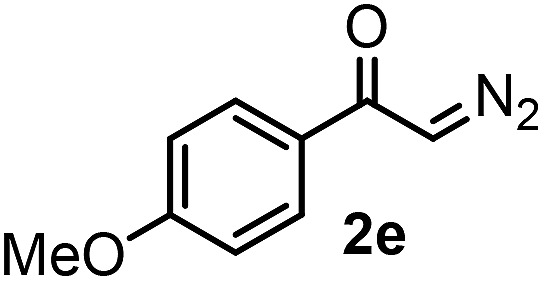	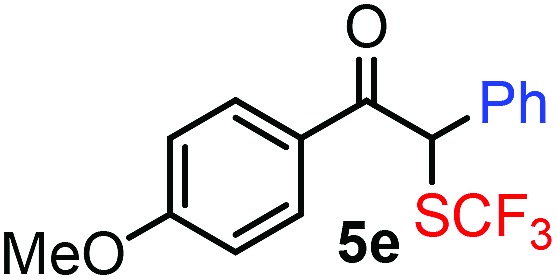	60
6	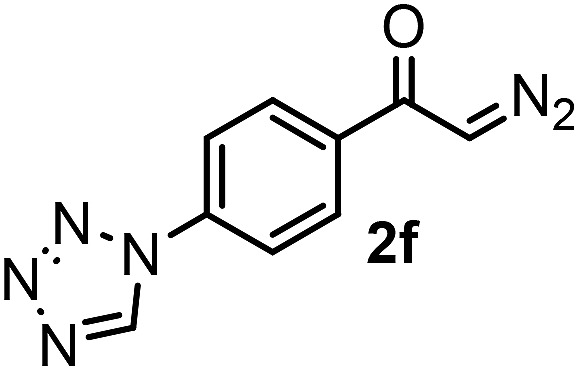	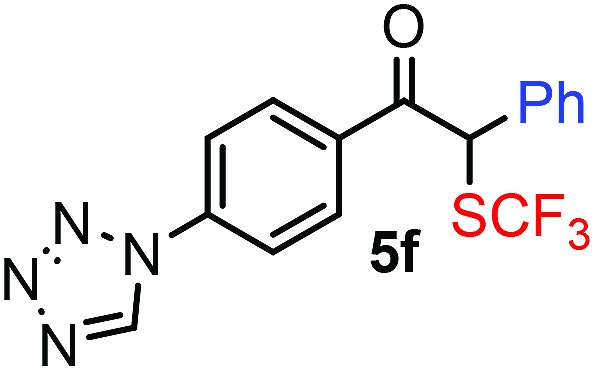	68^*c*^
7	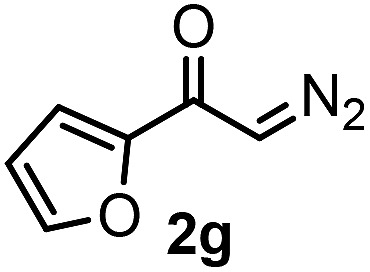	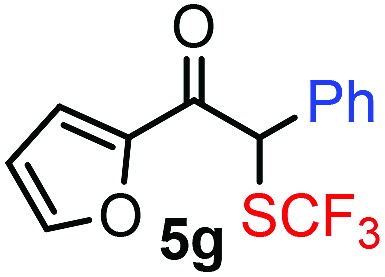	84
8	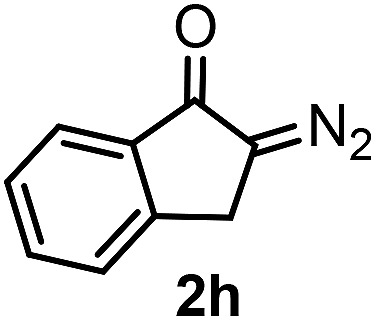	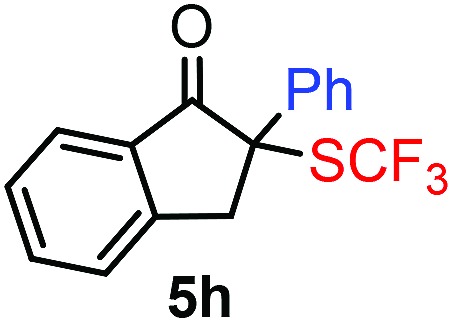	30^*c*^
9	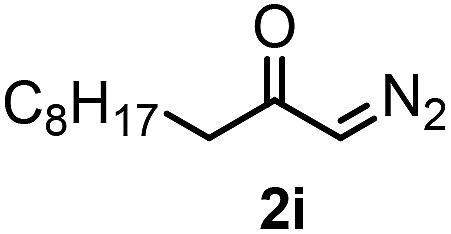	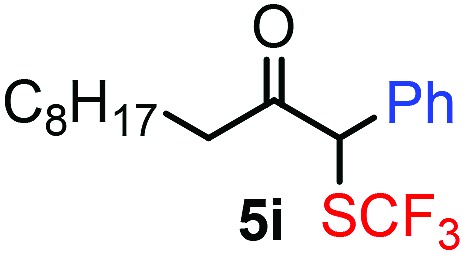	83
10	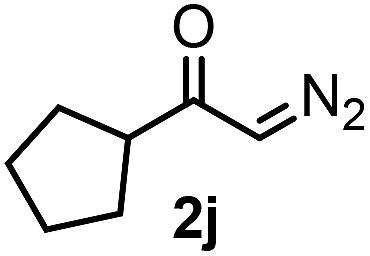	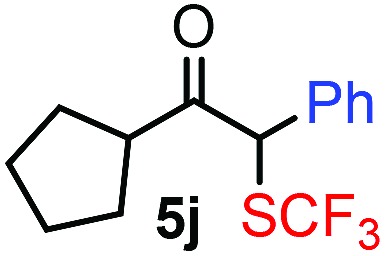	48
11	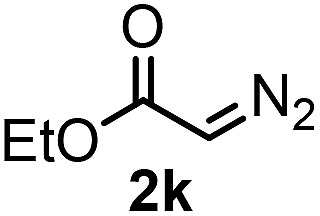	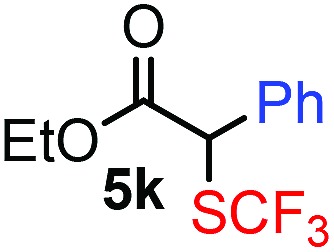	78(74)^*d*^
12	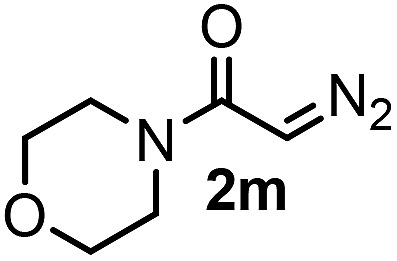	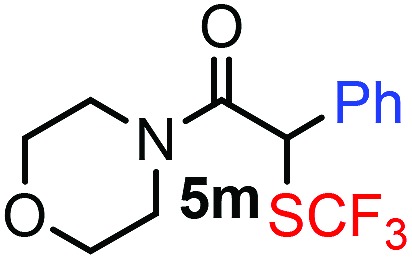	80
13	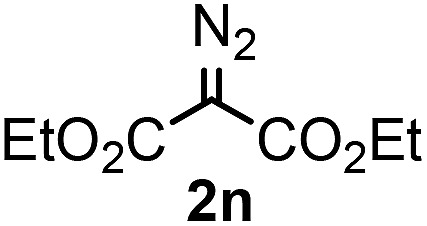	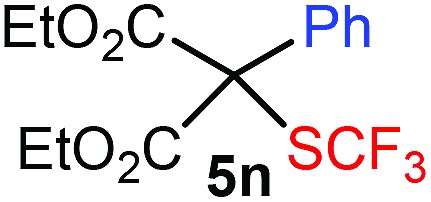	0
14	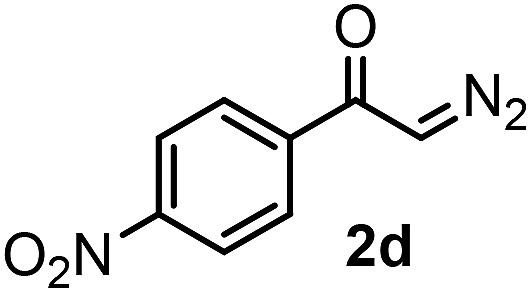	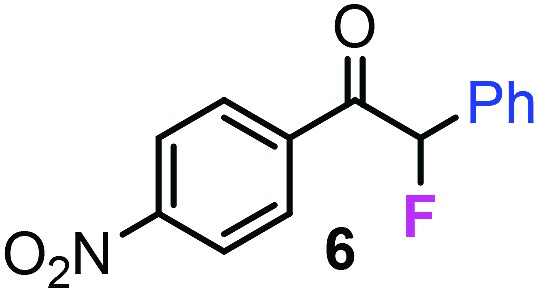	55^*c*^ ^,^^*e*^ ^,^^*f*^

^*a*^Unless otherwise stated: to **1** (0.1 mmol), Bu_4_N(BPh_4_) (**3c**) (0.15 mmol), Zn(NTf_2_)_2_ (**4**) (0.05 mmol) and 3Å ms (80 mg) was added a solution of **2** (0.15 mmol) in CH_2_Cl_2_ (1.0 ml) at –10 °C, stirred for 2 h before warmed up to RT overnight.

^*b*^Unless otherwise stated isolated yield.

^*c*^RT overnight.

^*d*^1.0 mmol scale.

^*e*^Instead of **1**, NFSI was used.

^*f*^Yield of **6** (determined by ^1^H-NMR analysis) contains side product 1-(4-nitrophenyl)-2-phenylethan-1-one.

We have also varied the aryl source, BAr_4_, of the bifunctionalization reaction (eqn (1)–(4)). When the phenyl substituent of **3c** was replaced by other aryl groups, we had to slightly modify the reactions conditions, such as the counter ion of the BAr_4_ reagent and/or the reaction temperature. The yields with these reagents were lower (36–48%) than with **3c**. When the chloro-phenyl derivative **3d** and **1** were reacted with diazoester **2k** or diazoketone **2o**, the corresponding bifunctionalized products **5o** and **5p** were formed in 48% and 47% yields, respectively (eqn (1) and (2)). These species have one (**5o**) or two (**5p**) aromatic halogenide handles for further functionalization by cross-coupling reactions. The reaction with thiophene transfer reagent **3e** was conducted at room temperature affording **5q** in 45% yield (eqn (3)). Trifluoromethylthio product **5r** with two different heterocyclic rings was obtained in 36% yield by reaction of **2g** and **3e** with **1** at room temperature (eqn (4)). We also attempted to perform alkylation–trifluoromethylthiolation reactions using Bu_4_N(BBu_4_), **3f**. However, in this reaction (eqn (5)) formation of **5s** was not observed, instead Bu-SCF_3_ (**7**) was formed by the rapid reaction of **1** and **3f**.1
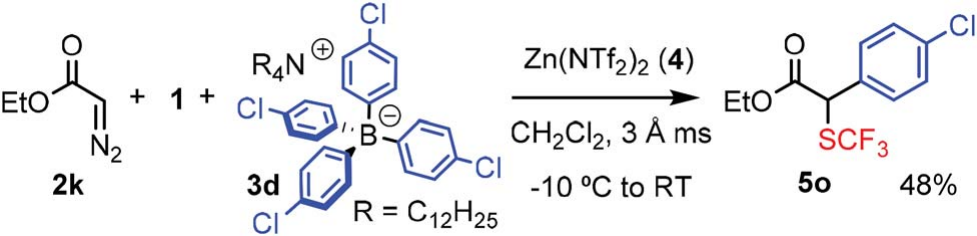

2
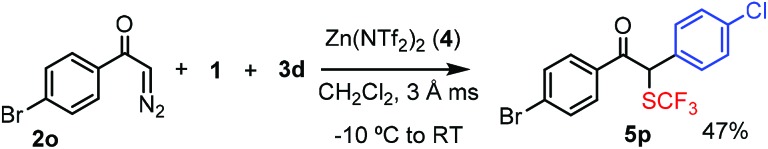

3
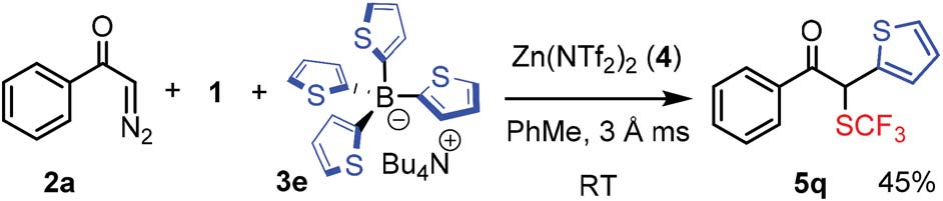

4
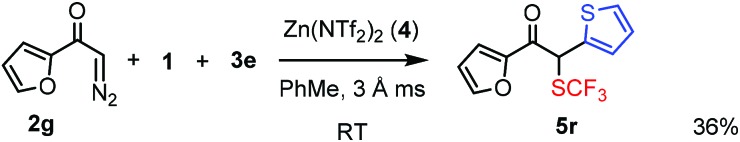

5
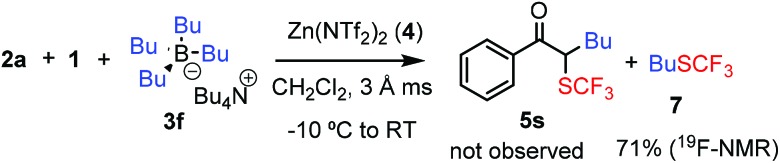



In order to get insights into the reaction mechanism, we performed a couple of control experiments (Scheme 3). First, we wanted to determine the sequence of the reactions among the four reaction components, such as **1**, **2a**, **3c** and **4**. Therefore, the systematic changes of the ^11^B NMR spectrum of the reaction of Bu_4_N(BPh_4_) **3c** and Zn(NTf_2_)_2_**4** was monitored. Pure **3c** gave a sharp ^11^B NMR signal at –6.6 ppm, which immediately disappeared, when an equimolar amount of Zn(NTf_2_)_2_ (**4**) was added (Scheme 3). The reaction of **3c** and **4** led to appearance of a new, broad signal at 67.6 ppm. The value of the ^11^B NMR shift and the broad shape of the signal indicated formation of BPh_3_**3b** from Bu_4_N(BPh_4_) **3c** by boron to zinc transmetallation.^16^ In this process PhZnNTf_2_ (**8**) was probably also formed. Phenylzinc derivative **8** rapidly reacted with residual water reversibly adsorbed by the molecular sieves to give benzene (**9**), which was observed by ^1^H NMR in the reaction mixture. The other product of the hydrolysis of PhZnNTf_2_ (**8**) is probably Zn(OH)NTf_2_ (**10**). When the resulted reaction mixture was reacted with diazoketone **2a** a new broad peak appeared at 45.6 ppm in the ^11^B NMR spectrum, which was assigned to vinyloxy-boronate **13**.^17^ Species like **13** are known to form in the Hooz reaction *via* formation of adduct **11**, followed by formation of **12** and a subsequent borotropic shift.^1^,17b Subsequent addition of dibenzenesulfonimide **1** to the reaction mixture led to the formation of trifluoromethylthiolation product **5a**, which could be observed by ^19^F NMR. In this last step (**13** → **5a**), the Zn-mediator **4** or its hydroxy derivative **10** may play an important role. For example, the electrophilic SCF_3_ transfer may be accelerated by coordination of Zn to the oxygen atom of **13**, which facilitates the cleavage of the boron–oxygen bond and delivery of **14**. As mentioned above, the three-component reaction of **2a**, BPh_3_ (**3b**) and **1** proceeds with a notoriously low yield (Scheme 2c, and Table 1/entry 10). This yield could not be improved by using strictly dry conditions in the reaction. However, a high yield was observed for a large variety of diazocarbonyl compounds, when **3c**/**4** conditions were used involving: (a) *in situ* generation of BPh_3_ (Scheme 3), (b) *in situ* removal of H_2_O and (c) Zn-mediated assistance of the electrophilic attack on **13**.

**Scheme 3:**
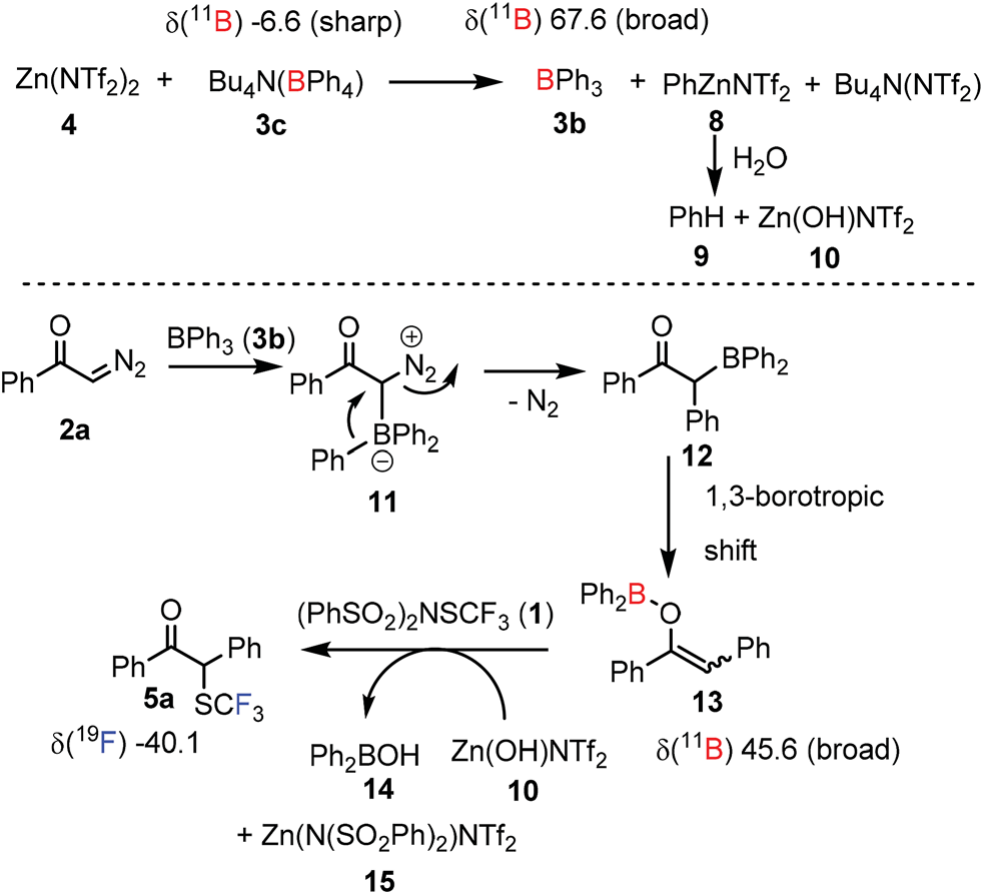
Suggested mechanism for the 1,1-trifluoro-methylthiolation–arylation of diazocarbonyl compounds. Chemical shifts (δ) are given in ppm.

Further control reactions (Scheme 4) confirmed the above mechanistic suggestions (Scheme 3). As mentioned above, Zn(OTf)_2_ (**16**), which is a close analog of Zn(NTf_2_)_2_ (**4**) did not mediate the reaction of **2a**, **3a** and **1** to form **5a** (Table 1, entry 6). This may be explained by the fact that the reaction of **16** and **3c** did not lead to an *in situ* formation of BPh_3_**3b** (Scheme 4a), which is the prerequisite for the formation of **5a***via***11** (Scheme 3). A further confirmation of the Hooz-type reaction mechanism *via* vinyloxy-boronate intermediate **13** arose from the control reaction, when **1** was replaced by another electrophile, such as benzaldehyde derivative **17** (Scheme 4b). The result of this reaction was formation of **18**, which most probably formed by reaction of **13** and **17** in the terminating step of the reaction. As mentioned above, the phenylation–fluorination reaction (Table 2, entry 12) with NFSI occurred with much lower yield than the corresponding phenylation–trifluoromethylthiolation with **1** (Table 2, entry 4). In the fluorination reaction, significant amounts of protonation product formed presumably because of competing electrophilic protonation of **13**. This led us to the conclusion that **1** is a better electrophile than NFSI in this bifunctionalization reaction (see above). Indeed, when we performed a competitive reaction of **2a**, **3a** and equimolar amounts of **1** and NFSI, we observed exclusive formation of the trifluoromethylthiolated product **5a** without formation of the fluorinated product **19**.

**Scheme 4:**
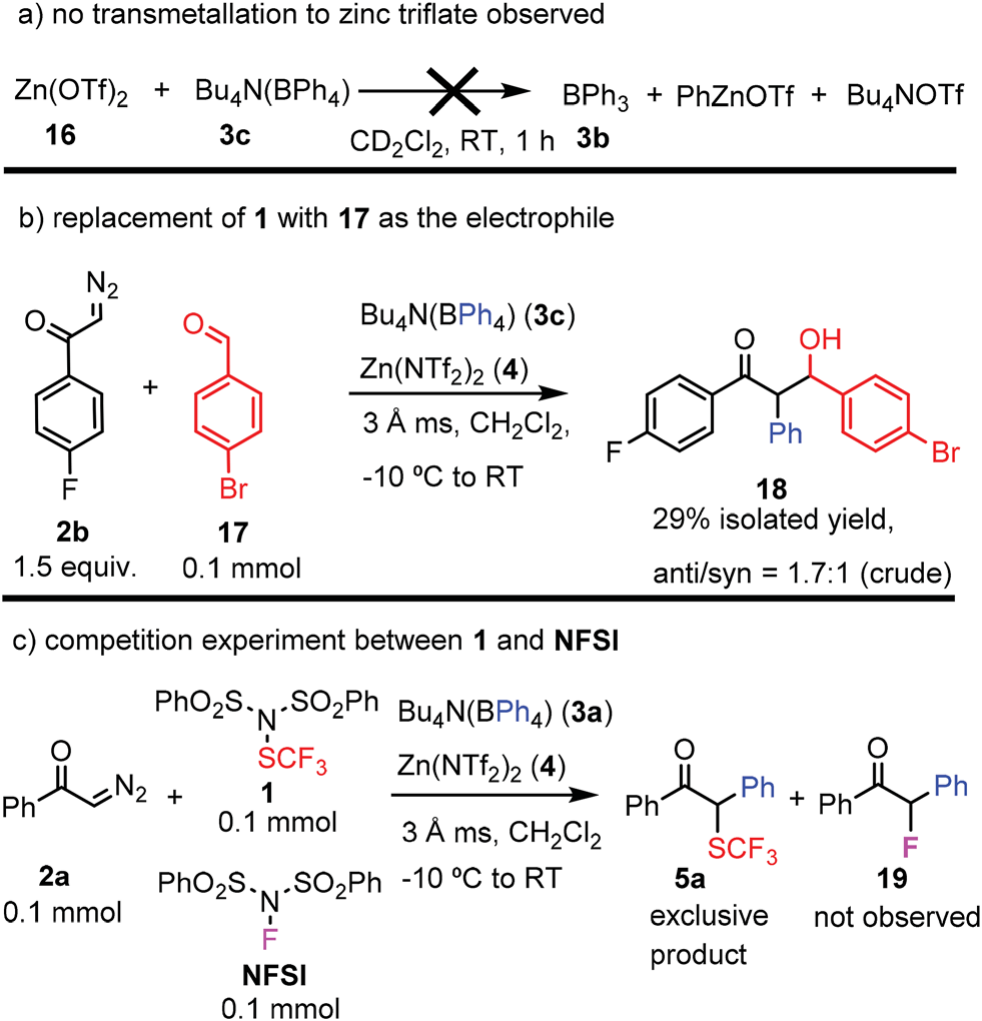
Control experiments to support the mechanism given in Scheme 3.

An alternative to the above Hooz-type mechanism could be an initial reaction of the PhZn species **8** directly with the diazo compound **2a** without involvement of BPh_3_. However, this hypothesis seems to be in conflict with the attempted phenylation–trifluoromethylthiolation reactions with ZnPh_2_ species without application of Zn(NTf_2_)_2_**4** (Table 1, entries 11 and 12). In these reactions the bifunctionalized product **5a** did not form.

In this report, we have described a new arylation–trifluoromethylthiolation reaction for an α,α′-bifunctionalization of diazocarbonyl compounds. This process can be performed as a multicomponent reaction, in which the aryl and SCF_3_ groups arise from different reagents, from **3** and **1**. This Hooz-type coupling is a novel approach for bifunctionalization based trifluoromethylthiolation, as for example the previously reported4*c* Rh-catalyzed oxy-trifluoromethylthiolation. The arylation–trifluoromethylthiolation reaction is initiated by Zn assisted formation of BAr_3_ from **3**. According to our mechanistic studies the reaction follows a Hooz-type reaction mechanism, which is terminated by electrophilic SCF_3_ transfer from **1**. As far as we know this is the first Hooz-type reaction for the synthesis of organofluorines from electrophilic transfer reagents. The reaction can also be extended to the phenylation–fluorination process, using the fluorine analog (NFSI) of **1**.

## Conflict of interest

The authors declare no competing financial interest.

## Supplementary Material

Supplementary information
