# Supplementary material for: Trifluoromethylthiolation–arylation of diazocarbonyl compounds by modified Hooz multicomponent coupling
Source: Chem Sci. 2019 May 6;10(23):5990–5. doi: 10.1039/c9sc00829b (PMC6571710; doi:10.1039/c9sc00829b)
Supplement: Supplementary file 1 [file SC-010-C9SC00829B-s001.pdf]

Electronic Supplementary Material (ESI) for Chemical Science.
This journal is © The Royal Society of Chemistry 2019

Supporting Information

Trifluoromethylthiolation-Arylation of Diazocarbonyl Compounds by Modified Hooz Multicomponent Coupling

Marvin Lübcke, Dina Bezhan and Kálmán J. Szabó*

Department of Organic Chemistry, Stockholm University
Arrhenius Laboratory, SE-106 91 Stockholm, Sweden

E-mail: kalman.j.szabo@su.se

Contents:

Page S2 General information

Page S3 Synthesis and characterization of starting materials

Page S4 General procedures for the 1,1-trifluoromethylthiolation-arylation and compound characterization

Page S13 Mechanistic experiments

Page S14 ^{1}H NMR, ^{13}C NMR and ^{19}F NMR spectra

Page S73 References

General Information

Diazo compounds **2a-j, m-o** were prepared according to literature procedures,[1-4] while trifluoromethylthiolating reagent **1** was synthesized according to a procedure by Shen and coworkers.[5] Reagents were used as obtained from commercial suppliers without further purification. $Bu_4N[BPh_4]$, $Na[BPh_4]$, K[B(*p*-Cl-Ph)$_4$], $Zn(NTf_2)_2$ and $Zn(OTf)_2$ were obtained from TCI. Ethyl diazoacetate (EDA, contains max. 15% CH_2Cl_2), (Trimethylsilyl)diazomethane solution (2 M in Et_2O), $(C_{12}H_{25})_4$N[B(*p*-Cl-Ph)$_4$], K[B(2-thiophene)$_4$], $Bu_4N(NTf_2)$, $Zn(NTf_2)_2$ and 3Å MS were obtained from Sigma-Aldrich. BPh_3 was obtained from Strem Chemicals. CH_2Cl_2, THF, Et_2O and toluene were dried by a solvent purification system (VAC Solvent Purifier from Vacuum Atmospheres Company). Extra dry CH_2Cl_2 was obtained from Sigma-Aldrich. MeCN was dried over activated 3 Å molecular sieves. Flash chromatography was carried out applying 60 Å (35-70 µm mesh) silica gel (VWR) using petroleum ether / Et_2O or petroleum ether / EtOAc mixtures as eluent. Analytical TLC was carried out on aluminum-backed plates (1.5 Å, ~ 5 cm) pre-coated (0.25 mm) with silica gel (Merck, Silica Gel 60 F254). Compounds were visualized by exposure to UV light or by dipping the plates in a solution of 0.75% $KMnO_4$ (w/v) in a aqueous solution of K_2CO_3 0.36 M. Melting points were recorded in a metal block instrument and are uncorrected.

^{1}H NMR spectra were recorded at 400 MHz; ^{13}C NMR spectra were recorded at 100 MHz, ^{19}F NMR spectra were recorded at 377 MHz and ^{11}B NMR spectra were recorded at 128 MHz with a Bruker Advance spectrometer. ^{1}H and ^{13}C NMR chemical shifts (δ) are reported in ppm from tetramethylsilane, using the residual solvent resonance (^{1}H-NMR: δ_H = 7.26 ppm ($CDCl_3$); 2.13 ppm (CD_3CN); 5.32 (CD_2Cl_2) and in ^{13}C-NMR: δ_C = 77.16 ppm (*C*DCl$_3$); 118.26 ppm (CD$_3$*C*N); 53.84 (*C*D$_2$Cl$_2$); as an internal references. Coupling constants (*J*) are given in Hz. High-resolution mass spectra (HRMS) were recorded with a Bruker microTOF ESI-TOF mass spectrometer in positive ion mode unless otherwise specified.

Experimental procedures and spctroscopic data

Preparation of starting materials

Tetrabutylammonium tetra(thiophen-2-yl)borate (3e)

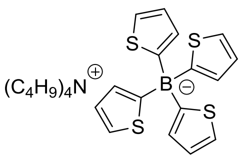

Title compound **3e** was prepared from commercial potassium tetra(thiophen-2-yl)borate by a slightly modified literature procedure.[6] Potassium tetra(thiophen-2-yl)borate (0.98 g, 2.6 mmol, 1.0 equiv.) was dissolved in dry THF (4.5 mL), then Bu_4NBr (1.9 g, 5.8 mmol, 2.2 equiv.) was added and the mixture was stirred for 30 min at room temperature. The reaction mixture was then filtered over silica gel and the solvent was removed under reduced pressure. The obtained crude product was dissolved in dry CH_2Cl_2 (15 mL) and filtered once again over silica gel. The obtained clear, beige colored solution was layered with dry Et_2O (approx 10 mL) and placed in a refrigerator. After two days, the obtained beige crystals were filtered off and dried in vacuo yielding **3e** (0.67 g, 1.1 mmol, 43% yield). **^{1}H NMR** (400 MHz, CD_3CN): δ = 7.09 (ddt, *J* = 4.7 Hz, 1.7 Hz, 0.9 Hz, 4H), 6.86 (dd, *J* = 4.7 Hz, 3.2 Hz, 4H), 6.79-6.76 (m, 4H), 3.05-2.98 (m, 8H), 1.59-1.49 (m, 8H), 1.35-1.25 (m, 8H), 0.92 (t, *J* = 8.3 Hz, 12H) ppm; **^{13}C NMR** (100 MHz, CD_3CN): δ = 165.9 (q, *J*(C,B) = 54.5 Hz), 129.5 (q, *J*(C,B) = 2.4 Hz), 126.9 (q, *J*(C,B) = 3.4 Hz), 124.4 (q, *J*(C,B) = 1.5 Hz), 59.3 (m), 24.3 (s), 20.3 (m), 13.8 (s) ppm; **^{11}B NMR** (128 MHz, CD_3CN): δ = -13.2 ppm; **HRMS** (ESI, negative mode): m/z calcd. for $C_{16}H_{12}{}^{11}BS_4^-$: 342.9923 $[M]^-$; found: 342.9907; **MP**: 162 ºC.

1-(4-(1*H*-Tetrazol-1-yl)phenyl)-2-diazoethan-1-one (2f)

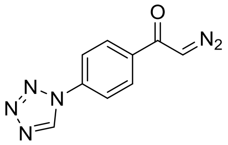

A solution of (trimethylsilyl)diazomethane (3.2 mmol; 1.6 mL, 1.2 equiv., 2.0 M in Et_2O) and Et_3N (4.0 mmol, 0.55 mL, 1.5 equiv.) in dry MeCN (7 mL) is cooled down to 0 ºC. To this mixture; a solution of 4-(1*H*-tetrazol-1-yl)benzoyl chloride[7] (2.6 mmol, 0.55 g, 1.0 equiv.) in dry MeCN (7 mL) is added slowly. The reaction mixture is allowed to warm up to room temperature (22 ºC). After 3vh of total reaction time, the solvent is evaporated under reduced pressure and the crude product is subjected to column chromatography (SiO_2; petroleum ether / EtOAc, 1:1) affording **2f** as a yellow solid (82 mg, 15% yield). **^{1}H NMR** (400 MHz, $CDCl_3$): δ = 9.08 (s, 1H), 8.02-7.97 (m, 2H), 7.88-7.83 (m, 2H), 5.97 (s, 1H) ppm; **^{13}C NMR** (100 MHz, $CDCl_3$): δ = 184.2, 140.4, 137.9, 136.7, 129.0, 121.2, 55.2 ppm; **HRMS** (ESI): m/z calcd. for $C_9H_6N_6O{+}Na^+$: 237.0495 $[M{+}Na]^+$; found: 237.0493.

General procedure A for the multicomponent reaction of diazo compounds 2 with tetraaryl borate salts 3 and trifluoromethylthio reagent 1 mediated by $Zn(NTf_2)_2$ (4)

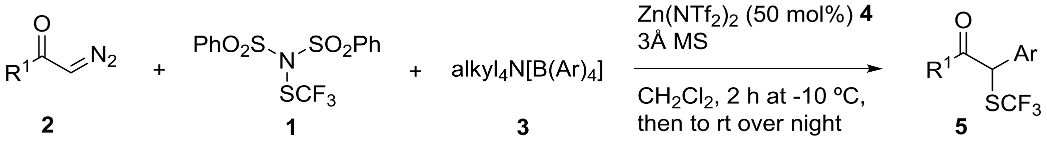

$(PhSO_2)_2NSCF_3$ (**1**) (40 mg, 0.1 mmol, 1.0 equiv.) was placed in a vial under ambient conditions together with a teflon stirring bar (3 x 10 mm). Separately, diazo compound **2** (0.15 mmol, 1.5 equiv.) was measured in a vial under ambient conditions. Both vials were transferred into an Ar-filled glove box. To the vial containing compound **1**, 3Å molecular sieves (80 mg), $Zn(NTf_2)_2$ (**4**) (31 mg, 0.05 mmol, 0.5 equiv.) and tetraalkylammonium tetraarylborate salt **3** (0.15 mmol, 1.5 equiv.) were added before the vial was sealed with an aluminum cap bearing a teflon/ rubber septum. The diazo compound **2** was dissolved in 1.0 mL dry CH_2Cl_2 and sealed in the same manner. Both vials were placed into a stirred cooling bath at -10 ºC and after 15 min, the solution of diazo compound **2** was added by syringe to the reaction vial (under Ar overpressure). The reaction mixture was stirred for 2 h at -10 ºC before it was allowed to warm to room temperature overnight. After evaporation of the reaction solvent, the crude mixture was purified by silica gel chromatography to obtain products **5**.

General procedure B for the multicomponent reaction of diazo compounds 2 with tetrabutylammonium tetra(thiophen-2-yl)borate 3e and trifluoromethylthio reagent 1 mediated by $Zn(NTf_2)_2$ (4)

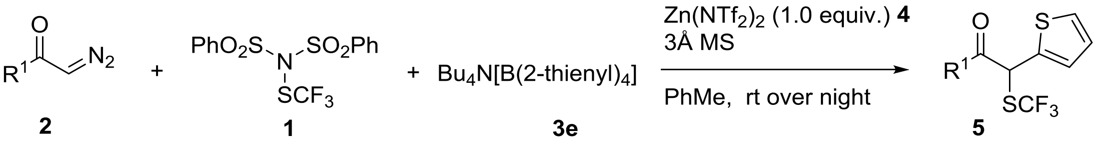

$(PhSO_2)_2NSCF_3$ (**1**) (40 mg, 0.1 mmol, 1.0 equiv.) was placed in a vial under ambient conditions together with a teflon stirring bar (3 x 10 mm). Separately, diazo compound **2** (0.15 mmol, 1.5 equiv.) was measured in a vial under ambient conditions. Both vials were transferred into an Ar-filled glove box. To the vial containing compound **1**, 3Å molecular sieves (80 mg), $Zn(NTf_2)_2$ (**4**) (63 mg, 0.1 mmol, 1.0 equiv.) and tetrabutylammonium tetra(thiophen-2-yl)borate (**3e**) (0.15 mmol, 1.5 equiv.) were added before the vial was sealed with an aluminum cap bearing a teflon/ rubber septum. The diazo compound **2** was dissolved in 1.0 mL of dry toluene and closed in the same manner. Both vials were placed into a stirred

cooling bath at -10 ºC and after 15 min, the solution of diazo compound **2** was added by syringe to the reaction vial (under Ar overpressure). The reaction mixture was taken out from the cooling bath and stirred then at room temperature overnight. The crude mixture was purified by silica gel chromatography to obtain products **5**.

1,2-Diphenyl-2-((trifluoromethyl)thio)ethan-1-one (5a)

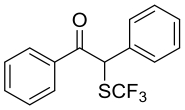

Title compound **5a** was prepared according to general procedure A and purified by column chromatography (SiO_2; petroleum ether / Et_2O, 50:1) affording **5a** as an off-white solid (26 mg, 81% yield). **^{1}H NMR** (400 MHz, $CDCl_3$): δ = 7.99-7.92 (m, 2H), 7.58-7.51 (m, 1H), 7.49-7.39 (m, 4H), 7.38-7.27 (m, 3H), 6.13 (s, 1H) ppm; **^{13}C NMR** (100 MHz, $CDCl_3$): δ = 193.2, 135.3, 134.4, 134.1, 130.7 (q, *J*(C,F) = 307.7 Hz), 129.6, 129.3, 129.0, 129.0, 128.7, 56.4 (q, *J*(C,F) = 1.8 Hz) ppm; **^{19}F NMR** (377 MHz, $CDCl_3$) ppm: δ = -40.05 ppm; **HRMS** (ESI): m/z calcd. for $C_{15}H_{11}OF_3S+Na^+$: 319.0375 [*M*+Na]$^+$; found: 319.0370; **MP**: 47 ºC.
The spectroscopic data are in agreement with the literature values.[8]

1-(4-Fluorophenyl)-2-phenyl-2-((trifluoromethyl)thio)ethan-1-one (5b)

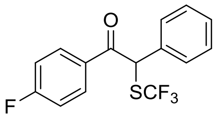

Title compound **5b** was prepared according to general procedure A and purified by column chromatography (SiO_2; petroleum ether / Et_2O, 50:1) affording **5b** as a colorless oil (26 mg, 80% yield). **^{1}H NMR** (400 MHz, $CDCl_3$): δ = 8.00-7.95 (m, 2H), 7.45-7.41 (m, 2H), 7.39-7.29 (m, 3H), 7.13-7.06 (m, 2H), 6.07 (s, 1H) ppm; **^{13}C NMR** (100 MHz, $CDCl_3$): δ = 191.7, 166.3 (d, *J*(C,F) = 257.2 Hz), 135.2, 132.0 (d, *J*(C,F) = 9.6 Hz), 130.8 (d, *J*(C,F) = 3.0 Hz), 130.6 (q, *J*(C,F) = 307.8 Hz), 129.6, 129.2, 128.7, 116.3 (d, *J*(C,F) = 22.1 Hz), 56.4 (q, *J*(C,F) = 1.8 Hz) ppm; **^{19}F NMR** (377 MHz, $CDCl_3$): δ = -40.05 (s, 3F), -(102.83-102.91) (m, 1F) ppm; **HRMS** (ESI): m/z calcd. for $C_{15}H_{10}OF_4S+Na^+$: 337.0281 [*M*+Na]$^+$; found: 337.0278.

1-(2-Iodophenyl)-2-phenyl-2-((trifluoromethyl)thio)ethan-1-one (5c)

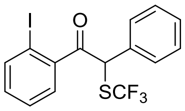

Title compound **5c** was prepared according to general procedure A and purified by column chromatography (SiO_2; petroleum ether / Et_2O, 50:1) affording **5c** as a pale yellow solid (31 mg, 73% yield). **^{1}H NMR** (400 MHz, $CDCl_3$): δ = 7.87 (dd, *J*= 7.9 Hz, 1.1 Hz, 1H), 7.36-7.30 (m, 5H), 7.28 (dd, *J*= 7.6 Hz, 1.1 Hz, 1H), 7.13 (dd, *J*= 7.7 Hz, 1.7 Hz, 1H), 7.08 (td, *J*= 7.7 Hz, 1.7Hz, 1H), 5.99 (s, 1H) ppm; **^{13}C NMR** (100 MHz, $CDCl_3$): δ = 196.6, 142.0, 140.9, 133.8, 132.4, 130.6 (q, *J*(C,F) =

307.9 Hz), 129.4, 129.2, 129.1, 129.0, 128.0, 92.0, 58.8 (q, *J*(C,F) = 1.8 Hz) ppm; ^{19}**F NMR** (377 MHz, $CDCl_3$): δ = -39.83 ppm; **HRMS** (ESI): m/z calcd. for $C_{15}H_{10}OF_3SI+Na^+$: 444.9341 [*M*+Na]$^+$; found: 444.9343; **MP**: 30 ºC.

1-(4-Nitrophenyl)-2-phenyl-2-((trifluoromethyl)thio)ethan-1-one (5d)

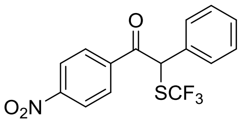

Title compound **5d** was prepared according to general procedure A and purified by column chromatography (SiO_2; petroleum ether / Et_2O, 20:1) affording **5d** as colorless oil that could not be crystallized (28 mg, 84% yield). ^{1}**H NMR** (400 MHz, $CDCl_3$): δ = 8.29-8.24 (m, 2H), 8.10-8.05 (m, 2H), 7.44-7.31 (m, 5H), 6.08 (s, 1H) ppm; ^{13}**C NMR** (100 MHz, $CDCl_3$): δ = 191.9, 150.7, 139.0, 134.0, 130.3 (q, *J*(C,F) = 308.0 Hz), 130.2, 129.9, 129.6, 128.7, 124.2, 56.7 (q, *J*(C,F) = 2.0 Hz) ppm; ^{19}**F NMR** (377 MHz, $CDCl_3$): δ = -40.01 ppm; **HRMS** (ESI, negative mode): m/z calcd. for $C_{15}H_{10}F_3NO_3S-H^+$: 340.0261 [*M*-H$^+$]$^-$; found: 340.0268.

1-(4-Methoxyphenyl)-2-phenyl-2-((trifluoromethyl)thio)ethan-1-one (5e)

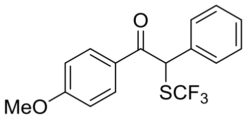

Title compound **5e** was prepared according to general procedure A and purified by column chromatography (SiO_2; petroleum ether / Et_2O, 50:1 to 30:1) affording **5e** as a white solid (20 mg, 60% yield). ^{1}**H NMR** (400 MHz, $CDCl_3$): δ = 7.95-7.91 (m, 2H), 7.47-7.43 (m, 2H), 7.37-7.32 (m, 2H), 7.31-7.26 (m, 1H), 6.91-6.87 (m, 2H), 6.08 (s, 1H), 3.83 (s, 3H) ppm; ^{13}**C NMR** (100 MHz, $CDCl_3$): δ = 191.7, 164.3, 135.9, 131.7, 130.8 (q, *J*(C,F) = 307.7 Hz), 129.5, 128.9, 128.6, 127.2, 114.3, 56.4 (q, *J*(C,F) = 1.7 Hz), 55.7 ppm; ^{19}**F NMR** (377 MHz, $CDCl_3$): δ = -40.03 ppm; **HRMS** (ESI): m/z calcd. for $C_{16}H_{13}O_2F_3S+Na^+$: 349.0481 [*M*+Na]$^+$; found: 349.0485; **MP**: 50 ºC.

1-(4-(1*H*-Tetrazol-1-yl)phenyl)-2-phenyl-2-((trifluoromethyl)thio)ethan-1-one (5f)

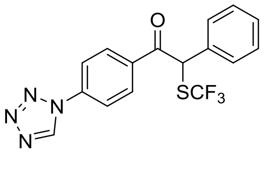

Title compound **5f** was prepared according to general procedure A with a slight modification: the reaction was run at room temperature due low solubility of the diazo starting material at -10 ºC. **5f** was purified by column chromatography (SiO_2; petroleum ether / EtOAc, 2:1) affording **5f** as a pale yellow solid (25 mg, 68% yield). ^{1}**H NMR** (400 MHz, $CDCl_3$): δ = 9.05 (s, 1H), 8.19-8.13 (m, 2H), 7.87-7.80 (m, 2H), 7.47-7.30 (m, 5H), 6.11 (s, 1H) ppm; ^{13}**C NMR** (100 MHz, $CDCl_3$): δ = 191.6, 140.4, 137.4, 135.3, 134.4, 131.4, 130.4 (q, *J*(C,F) = 307.8 Hz), 129.9, 129.5, 128.7, 121.2, 56.6 (q, *J*(C,F) = 1.9 Hz) ppm; ^{19}**F NMR** (377 MHz,

CDCl$_3$): δ = -39.97 ppm; **HRMS** (ESI): m/z calcd. for $C_{16}H_{11}OF_3N_4S+Na^+$: 387.0498 [$M$+Na]$^+$; found: 387.0498; **MP**: 156 ºC.

1-(Furan-2-yl)-2-phenyl-2-((trifluoromethyl)thio)ethan-1-one (5g)

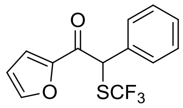

Title compound **5g** was prepared according to general procedure A and purified by column chromatography (SiO$_2$; petroleum ether / Et$_2$O, 50:1) affording **5g** as a pale yellow solid (26 mg, 84% yield). **^{1}H NMR** (400 MHz, CDCl$_3$): δ = 7.59 (dd, J= 1.7 Hz, 0.8 Hz, 1H), 7.51-7.47 (m, 2H), 7.38-7.30 (m, 3H), 7.29 (dd, J= 3.6 Hz, 0.8 Hz, 1H), 6.53 (dd, J= 3.6 Hz, 1.7 Hz, 1H), 5.93 (s, 1H) ppm; **^{13}C NMR** (100 MHz, CDCl$_3$): δ = 182.0, 150.5, 147.6, 134.7, 130.5 (q, J(C,F) = 307.8 Hz), 129.3, 129.0, 128.8, 119.8, 113.1, 55.1 (q, J(C,F) = 1.8 Hz) ppm; **^{19}F NMR** (377 MHz, CDCl$_3$): δ = -40.32 ppm; **HRMS** (ESI): m/z calcd. for $C_{13}H_9O_2F_3S+Na^+$: 309.0168 [M+Na]$^+$; found: 309.0169; **MP**: 72 ºC.

2-Phenyl-2-((trifluoromethyl)thio)-2,3-dihydro-1*H*-inden-1-one (5h)

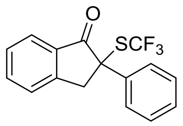

Title compound **5h** was prepared according to general procedure A with a slight modification: the reaction was run at room temperature in 0.5 mL of dry CH$_2$Cl$_2$. **5h** was purified by column chromatography (SiO$_2$; petroleum ether / Et$_2$O, 50:1) affording **5h** as a pale yellow oil (9 mg, 30% yield). **^{1}H NMR** (400 MHz, CDCl$_3$): δ = 7.85-7.81 (m, 1H), 7.71-7.66 (m, 1H), 7.65-7.61 (m, 2H), 7.52-7.49 (m, 1H), 7.47-7.41 (m, 1H), 7.37-7.26 (m, 3H), 4.12 (d, J_{gem} = 17.9 Hz, 1H), 4.00 (d, J_{gem} = 17.9 Hz, 1H) ppm; **^{13}C NMR** (100 MHz, CDCl$_3$): δ = 199.4, 150.3, 136.9, 136.3, 133.7, 129.9 (q, J(C,F) = 310.1 Hz), 129.0, 128.6, 128.5, 127.5, 126.0, 125.8, 62.7, 43.6 (q, J(C,F) = 1.2 Hz) ppm; **^{19}F NMR** (377 MHz, CDCl$_3$): δ = -36.84 ppm; **HRMS** (ESI): m/z calcd. for $C_{16}H_{11}OF_3S+Na^+$: 331.0375 [M+Na]$^+$; found: 331.0370.

1-Phenyl-1-((trifluoromethyl)thio)undecan-2-one (5i)

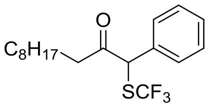

Title compound **5i** was prepared according to general procedure A and purified by column chromatography (SiO$_2$; petroleum ether / Et$_2$O, 50:1) affording **5i** as an off-white solid (32 mg, 83% yield). **^{1}H NMR** (400 MHz, CDCl$_3$): δ = 7.42-7.31 (m, 5H), 5.21 (s, 1H), 2.44 (t, J = 7.3 Hz, 2H), 1.60-1.44 (m, 2H), 1.31-1.09 (m, 12H), 0.87 (t, J = 7.0 Hz, 3H) ppm; **^{13}C NMR** (100 MHz, CDCl$_3$): δ = 203.5, 134.7, 130.5 (q, J(C,F) = 307.5 Hz), 129.5, 129.2, 128.6, 60.0 (q, J(C,F) = 1.5 Hz), 40.4, 32.0, 29.5, 29.3, 29.3, 28.9, 23.9, 22.8, 14.2 ppm; **^{19}F NMR** (377 MHz, CDCl$_3$): δ

= -40.05 ppm; **HRMS** (ESI): m/z calcd. for $C_{18}H_{25}OF_3S+Na^+$: 369.1470 [*M*+Na]$^+$; found: 369.1478; **MP**: 24 ºC.

1-Cyclopentyl-2-phenyl-2-((trifluoromethyl)thio)ethan-1-one (5j)

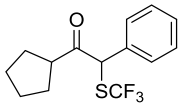

Title compound **5j** was prepared according to general procedure A and purified by column chromatography (SiO_2; petroleum ether / Et_2O, 50:1) affording **5j** as colorless oil (14 mg, 48% yield). **^{1}H NMR** (400 MHz, $CDCl_3$): δ = 7.40-7.32 (m, 5H), 5.31 (s, 1H), 2.97-2.88 (m, 1H), 1.96-1.85 (m,1H), 1.76-1.40 (m, 7H) ppm; **^{13}C NMR** (100 MHz, $CDCl_3$): δ = 206.1, 134.8, 130.6 (q, *J*(C,F) = 307.7 Hz), 129.4, 129.1, 128.8, 59.6 (q, *J*(C,F) = 1.8 Hz), 49.5, 30.7, 29.4, 26.2, 26.1 ppm; **^{19}F NMR** (377 MHz, $CDCl_3$): δ = -40.08 ppm; **HRMS** (ESI): m/z calcd. for $C_{14}H_{15}OF_3S+Na^+$: 311.0688 [*M*+Na]$^+$; found: 311.0690.

Ethyl 2-phenyl-2-((trifluoromethyl)thio)acetate (5k)

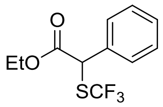

Title compound **5k** was prepared according to general procedure A and purified by column chromatography (SiO_2; petroleum ether / Et_2O, 50:1) affording **5k** as a colorless oil (20 mg, 78%). **^{1}H NMR** (400 MHz, $CDCl_3$): δ = 7.50-7.33 (m, 5H), 5.05 (s, 1H), 4.31-4.14 (m, AA'-system, 2H), 1.25 (t, *J* = 7.2 Hz, 3H) ppm; **^{13}C NMR** (100 MHz, $CDCl_3$): δ = 169.1, 134.1, 130.0 (q, *J*(C,F) = 308.0 Hz), 129.2, 129.2, 128.4, 62.8, 51.6 (q, *J*(C,F) = 2.3 Hz), 14.0 ppm; **^{19}F NMR** (377 MHz, $CDCl_3$): δ = -41.14 ppm; **HRMS** (ESI): m/z calcd. for $C_{11}H_{11}O_2F_3S+Na^+$: 287.0324 [*M*+Na]$^+$; found: 287.0330.

The spectroscopic data are in agreement with the literature values.[9]

Ethyl 2-phenyl-2-((trifluoromethyl)thio)acetate 5k at 1.0 mmol scale

The above preparation of **5k** was repeated at 1.0 mmol scale: $(PhSO_2)_2NSCF_3$ (**1**) (397 mg, 1.0 mmol, 1.0 equiv.) was placed in a 20 mL Biotage® microwave reaction vial under ambient conditions. In an Ar-filled glove box, 800 mg of 3 Å molecular sieves, $Zn(NTf_2)_2$ (**4**) (313 mg, 0.5 mmol, 0.5 equiv.) and tetrabutylammonium tetraphenylborate (**3a**) (843 mg, 1.5 mmol, 1.5 equiv.) were added. EDA (**2k**) was dissolved in 10 mL of dry CH_2Cl_2 and added to the solids at -10 ºC. The reaction mixture was stirred for 2 h at -10 ºC before it was allowed to warm to room temperature overnight. After evaporation of the reaction solvent, the crude mixture was purified by silica gel chromatography (SiO_2; petroleum ether / Et_2O, 50:1) to obtain product **5k** (193 mg, 74% yield).

1-Morpholino-2-phenyl-2-((trifluoromethyl)thio)ethan-1-one (5m)

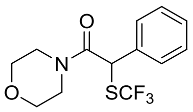

Title compound **5m** was prepared according to general procedure A and purified by column chromatography (SiO_2; petroleum ether / EtOAc, 4:1) affording **5m** as a colorless oil (25 mg, 80%). **^{1}H NMR** (400 MHz, $CDCl_3$): δ = 7.43-7.32 (m, 5H), 5.37 (s, 1H), 3.76-3.61 (m, 2H), 3.58-3.41 (m, 4H), 3.39-3.27 (m, 1H), 3.14-3.03 (m, 1H) ppm; **^{13}C NMR** (100 MHz, $CDCl_3$): δ = 166.5, 136.2, 130.7 (q, *J*(C,F) = 307.9 Hz), 129.4, 129.1, 128.2, 66.6, 66.1, 52.8 (q, *J*(C,F) = 1.9 Hz), 46.7, 43.1 ppm; **^{19}F NMR** (377 MHz, $CDCl_3$): δ = -40.24 ppm; **HRMS** (ESI): m/z calcd. for $C_{13}H_{14}NO_2F_3S+Na^+$: 328.0595 [$M$+Na]$^+$; found: 328.0594.

Ethyl 2-(4-chlorophenyl)-2-((trifluoromethyl)thio)acetate (5o)

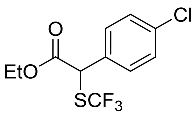

Title compound **5o** was prepared according to general procedure A and purified by column chromatography (SiO_2; petroleum ether / Et_2O, 50:1) affording **5o** as a colorless oil (15 mg, 48% yield). **^{1}H NMR** (400 MHz, $CDCl_3$): δ = 7.41-7.34 (m, 4H), 5.01 (s, 1H), 4.30-4.14 (m, AA'-system, 2H), 1.25 (t, *J* = 7.1 Hz, 3H) ppm; **^{13}C NMR** (100 MHz, $CDCl_3$): δ = 168.7, 135.3, 132.9, 129.9 (q, *J*(C,F) = 308.1 Hz), 129.7, 129.5, 63.0, 51.1 (q, *J*(C,F) = 2.3 Hz), 14.0 ppm; **^{19}F NMR** (377 MHz, $CDCl_3$): δ = -40.98 ppm; **HRMS** (ESI): m/z calcd. for $C_{11}H_{10}O_2F_3{}^{35}ClS+Na^+$: 320.9934 [$M$+Na]$^+$; found: 320.9927.

The Spectroscopic data are in agreement with the literature values.[9]

1-(4-Bromophenyl)-2-(4-chlorophenyl)-2-((trifluoromethyl)thio)ethan-1-one (5p)

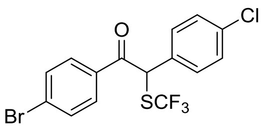

Title compound **5p** was prepared according to general procedure A and purified by column chromatography (SiO_2; petroleum ether / Et_2O, 50:1) affording **5p** as a pale yellow oil that could not be crystallized (21 mg, 47% yield). **^{1}H NMR** (400 MHz, $CDCl_3$): δ = 7.80-7.75 (m, 2H), 7.60-7.56 (m, 2H), 7.38-7.31 (m, 4H), 6.02 (s, 1H) ppm; **^{13}C NMR** (100 MHz, $CDCl_3$): δ = 192.0, 135.4, 133.6, 132.8, 132.5, 130.6, 130.5 (q, *J*(C,F) = 307.9 Hz), 130.0, 129.9, 129.9, 55.6 (q, *J*(C,F) = 1.9 Hz) ppm; **^{19}F NMR** (377 MHz, $CDCl_3$): δ = -39.89 ppm; **HRMS** (ESI): m/z calcd. for $C_{15}H_9OF_3S^{35}Cl^{79}Br+Na^+$: 430.9090 [$M$+Na]$^+$; found: 430.9107.

1-Phenyl-2-(thiophen-2-yl)-2-((trifluoromethyl)thio)ethan-1-one (5q)

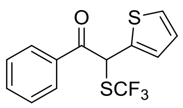

Title compound **5q** was prepared according to general procedure B and purified by column chromatography (SiO_2; petroleum ether / Et_2O, 50:1) affording **5q** as a pale yellow solid that quickly darkens (14 mg, 45% yield). **^{1}H NMR** (400 MHz, $CDCl_3$): δ = 8.02-7.97 (m, 2H), 7.62-7.57 (m, 1H), 7.50-7.44 (m, 2H), 7.33 (dd, *J* = 5.1 Hz, 1.2 Hz, 1H), 7.14 (ddd, *J* = 3.6 Hz, 1.2 Hz, 0.6 Hz, 1H), 6.95 (dd, *J* = 5.1 Hz, 3.6 Hz, 1H), 6.41 (s, 1H) ppm; **^{13}C NMR** (100 MHz, $CDCl_3$): δ = 192.2, 136.8, 134.4, 134.1, 130.4 (q, *J*(C,F) = 308.1 Hz), 129.3, 129.1, 128.6, 128.0, 127.6, 50.6 (q, *J*(C,F) = 2.0 Hz) ppm; **^{19}F NMR** (377 MHz, $CDCl_3$) ppm: δ = -40.27 ppm; **HRMS** (ESI): m/z calcd. for $C_{13}H_9OF_3S_2+Na^+$: 324.9939 [*M*+Na]$^+$; found: 324.9943; **MP**: 59 ºC.

1-(Furan-2-yl)-2-(thiophen-2-yl)-2-((trifluoromethyl)thio)ethan-1-one (5r)

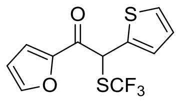

Title compound **5r** was prepared according to general procedure B and purified by column chromatography (SiO_2; petroleum ether / Et_2O, 50:1) affording **5r** as a pale yellow solid that quickly darkens (11 mg, 36% yield). **^{1}H NMR** (400 MHz, $CDCl_3$): δ = 7.65 (dd, *J* = 1.7 Hz, 0.8 Hz, 1H), 7.37 (dd, *J* = 3.7 Hz, 0.8 Hz, 1H), 7.33 (dd, *J* = 5.2 Hz, 1.3 Hz, 1H), 7.17 (ddd, *J* = 3.6 Hz, 1.3 Hz, 0.6 Hz, 1H), 6.96 (dd, *J* = 5.2 Hz, 3.6 Hz, 1H), 6.59 (dd, *J* = 3.7 Hz, 1.7 Hz, 1H), 6.22 (s, 1H) ppm; **^{13}C NMR** (100 MHz, $CDCl_3$): δ = 181.0, 150.1, 147.8, 136.1, 130.2 (q, *J*(C,F) = 308.3 Hz), 128.5, 127.9, 127.4, 120.2, 113.4, 49.4 (q, *J*(C,F) = 2.2 Hz) ppm; **^{19}F NMR** (377 MHz, $CDCl_3$) ppm: δ = -40.52 ppm; **HRMS** (ESI): m/z calcd. for $C_{11}H_7O_2F_3S_2+Na^+$: 314.9732 [*M*+Na]$^+$; found: 314.9718; **MP**: 43 ºC.

2-Fluoro-1-(4-nitrophenyl)-2-phenylethan-1-one (6)

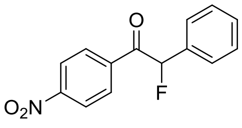

6

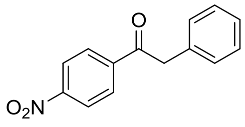

1-(4-nitrophenyl)-2-phenylethan-1-one

Title compound **6** was prepared according to general procedure A with a slight modification: the reaction was run at room temperature. **6** was purified by column chromatography (SiO_2; petroleum ether / EtOAc, 10:1) affording **6** as a pale yellow solid in a mixture with 1-(4-nitrophenyl)-2-phenylethan-1-one in a 1:1-molar ratio (determined by ^{1}H NMR; 29 mg, 55% yield of **6**). It was not possible to separate these two compounds by flash chromatography, neither could they be distinguished with thin layer chromatography (TLC). 1-(4-nitrophenyl)-2-phenylethan-1-one could only be removed by a chemical method relying on a recent publication for a-hydroxylation of ketones.[10]

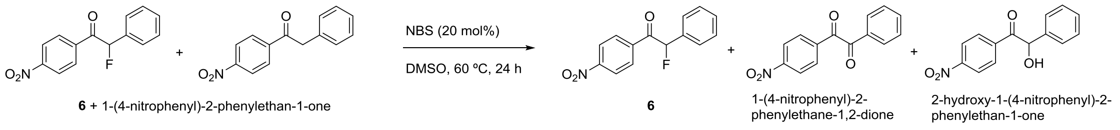

The oxidation procedure had to be carried out twice in order to convert 1-(4-nitrophenyl)-2-phenylethan-1-one into more polar oxidation products which could be removed by column chromatography (SiO_2; petroleum ether / EtOAc, 10:1) affording **6** as a colorless solid (6 mg, 23% yield).

^{1}H NMR (400 MHz, $CDCl_3$): δ = 8.29-8.24 (m, 2H), 8.12-8.02 (m, 2H), 7.49-7.39 (m, 5H), 6.46 (d, *J*(H,F) = 48.6 Hz, 1H) ppm; **^{13}C NMR** (100 MHz, $CDCl_3$): δ = 193.5 (d, *J*(C,F) = 23.1 Hz), 150.6, 138.7 (d, *J*(C,F) = 1.5 Hz), 133.5 (d, *J*(C,F) = 20.1 Hz), 130.4 (d, *J*(C,F) = 3.7 Hz), 130.1 (d, *J*(C,F) = 2.5 Hz), 129.5, 127.1 (d, *J*(C,F) = 5.9 Hz), 124.0, 94.8 (d, *J*(C,F) = 187.9 Hz); **^{19}F NMR** (377 MHz, $CDCl_3$) ppm: δ = -177.55 (d, *J*(F,H) = 48.6 Hz) ppm; **HRMS** (ESI, negative mode): m/z calcd. for $C_{14}H_{10}FNO_3$-H^+: 258.0572 [M-H^+]$^-$; found: 258.0581; **MP**: 66 °C.

3-(4-Bromophenyl)-1-(4-fluorophenyl)-3-hydroxy-2-phenylpropan-1-one (18)

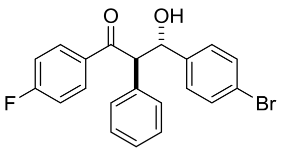

anti

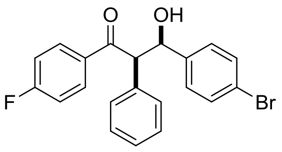

syn

Title compound **18** was prepared according to general procedure A with 4-bromobenzaldehyde instead of **1**. The initial diastereomeric ratio was determined by crude ^{19}F/^{1}H NMR analysis to be anti/syn = 1.7:1.[11] **18** was purified by column chromatography (SiO_2; petroleum ether / EtOAc, 8:1) affording **18** as a colorless solid

(11 mg, 29% yield) with a diastereomeric ratio of anti/syn = 7.7:1(determined by ^{19}F NMR analysis).

anti-**18**: **^{1}H NMR** (400 MHz, $CDCl_3$): δ = 8.00-7.93 (m, 2H), 7.33-7.28 (m, 2H), 7.19-7.15 (m, 3H), 7.07-7.01 (m, 2H), 7.00-6.96 (m, 2H), 6.96-6.92 (m, 2H), 5.33 (dd, *J* = 9.0 Hz, 3.4 Hz, C*H*OH, 1H), 4.65 (d, *J* = 9.0 Hz, 1H), 3.29 (d, *J* = 3.7 Hz, O*H*, 1H) ppm; **^{13}C NMR** (100 MHz, $CDCl_3$): δ = 198.7, 165.9 (d, *J*(C,F) = 256.0 Hz), 140.0, 135.1, 132.9 (d, *J*(C,F) = 3.0 Hz), 131.8 (d, *J*(C,F) = 9.5 Hz), 131.2, 129.2, 128.9, 128.5, 127.9, 121.7, 115.9 (d, *J*(C,F) = 22.0 Hz), 76.3, 62.7 ppm; **^{19}F NMR** (377 MHz, $CDCl_3$) ppm: δ = -(104.13-104.21) (m, 1F) ppm.

syn-**18**: **^{1}H NMR** (400 MHz, $CDCl_3$): δ = 7.89-7.84 (m, 2H), 7.39-7.35 (m, 2H), 7.27-6.91 (m, 9H), 5.48 (dd, *J* = 5.1 Hz, 1.9 Hz, C*H*OH, 1H), 4.66 (d, *J* = 5.1 Hz, 1H), 3.44 (d, *J* = 2.2 Hz, O*H*, 1H) ppm; **^{13}C NMR** (100 MHz, $CDCl_3$): δ = 199.0, 165.9 (d, *J*(C,F) = 256.2 Hz), 140.3, 133.7, 132.5 (d, *J*(C,F) = 2.9 Hz), 131.7 (d, *J*(C,F) = 9.5 Hz), 131.3, 131.2, 129.8, 128.9, 128.5, 128.1, 121.6, 116.0 (d, *J*(C,F) = 21.9 Hz), 74.1, 60.9 ppm; **^{19}F NMR** (377 MHz, $CDCl_3$) ppm: δ = -(103.91-103.98) (m, 1F) ppm.

HRMS (ESI): m/z calcd. for $C_{21}H_{16}{}^{79}BrFO_2$ +Na^+: 421.0210 [*M*+Na]$^+$; found: 421.0209.

Mechanistic NMR-experiments for the 1,1-trifluoromethylthiolation-arylation of diazocarbonyl compounds

All NMR samples were prepared in an argon-filled glove box.

Experiment a) Pure $Bu_4N(BPh_4)$ **3c** (0.05 mmol) in CD_2Cl_2 (0.5 mL) gave a sharp multiplett at -6.6 ppm (tetravalent boron center) in the ^{11}B NMR spectrum.

Experiment b) Pure BPh_3 (**3b**) (0.05 mmol) in CD_2Cl_2 (0.5 mL) showed a broad signal at 67.6 ppm in the ^{11}B NMR spectrum. The signal intensity was very weak due to low solubility in CD_2Cl_2.

Experiment c) When $Bu_4N(BPh_4)$ (**3c**) (0.05 mmol) and $Zn(NTf_2)_2$ (**4**) (0.05 mmol) were dissolved together in CD_2Cl_2 (0.5 mL) and a ^{11}B NMR spectrum was recorded immediately, only one signal at 67.6 ppm was visible, indicating formation of BPh_3 (**3b**) as the exclusive boron species.

Experiment d) To the solution of $Bu_4N(BPh_4)$ (**3c**) (0.05 mmol) and $Zn(NTf_2)_2$ (**4**) (0.05 mmol) in CD_2Cl_2 (0.5 mL) was added diazoacetophenone (**2a**) (0.05 mmol). Immediate ^{11}B NMR analysis showed a new signal at 45.6 ppm, which was assigned to vinyloxy borinate **13**.

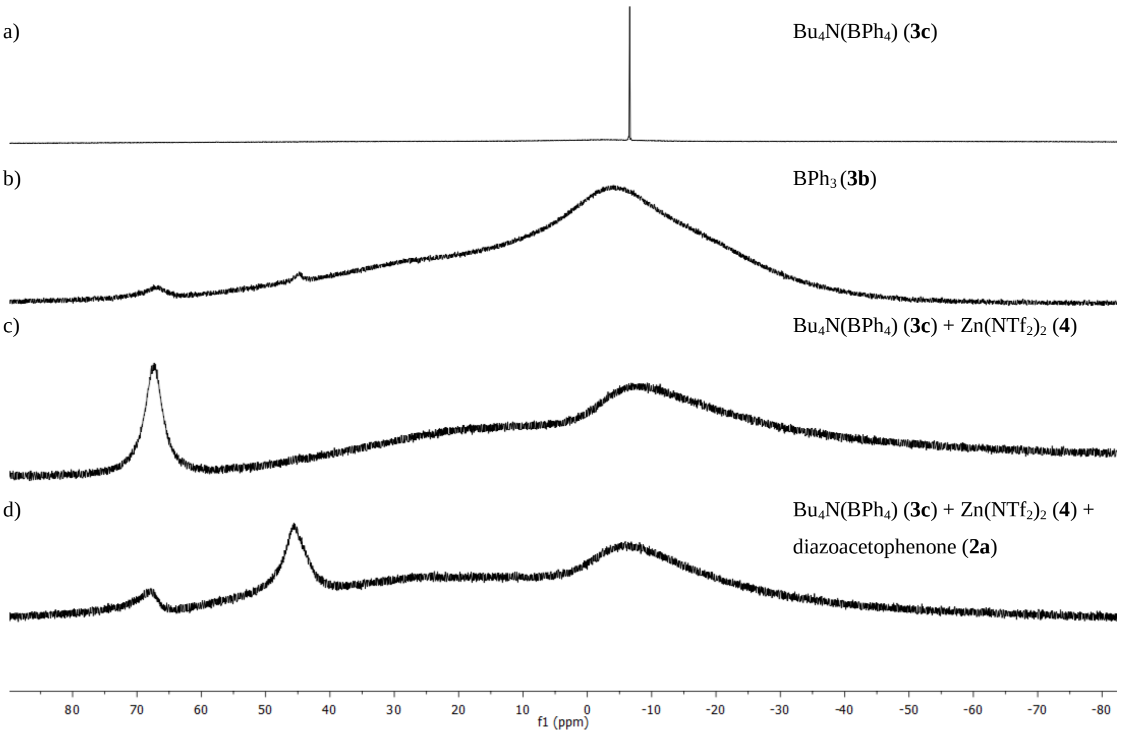

Figure S1. Monitoring the ^{11}B NMR spectra in experiments a-d at 22 °C. The broad peak between -10 ppm and 0 ppm arises from the borosilicate glass used in the experiments.

^{1}H NMR (CD_3CN, 400 MHz). Tetrabutylammonium tetra(thiophen-2-yl)borate (**3e**)

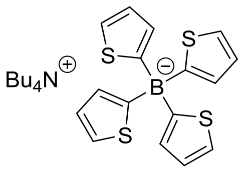

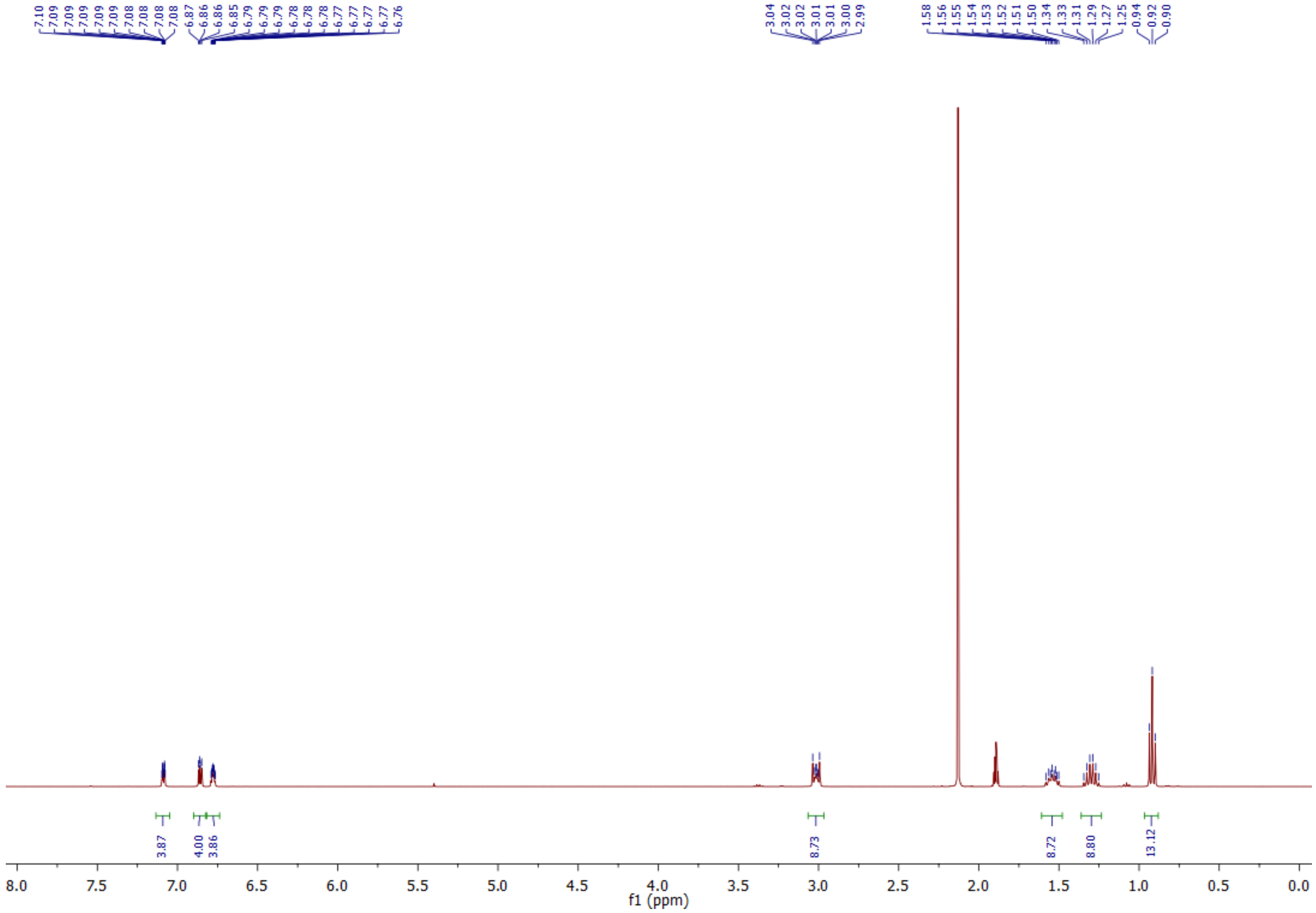

^{13}C NMR (CD_3CN, 100 MHz). Tetrabutylammonium tetra(thiophen-2-yl)borate (**3e**)

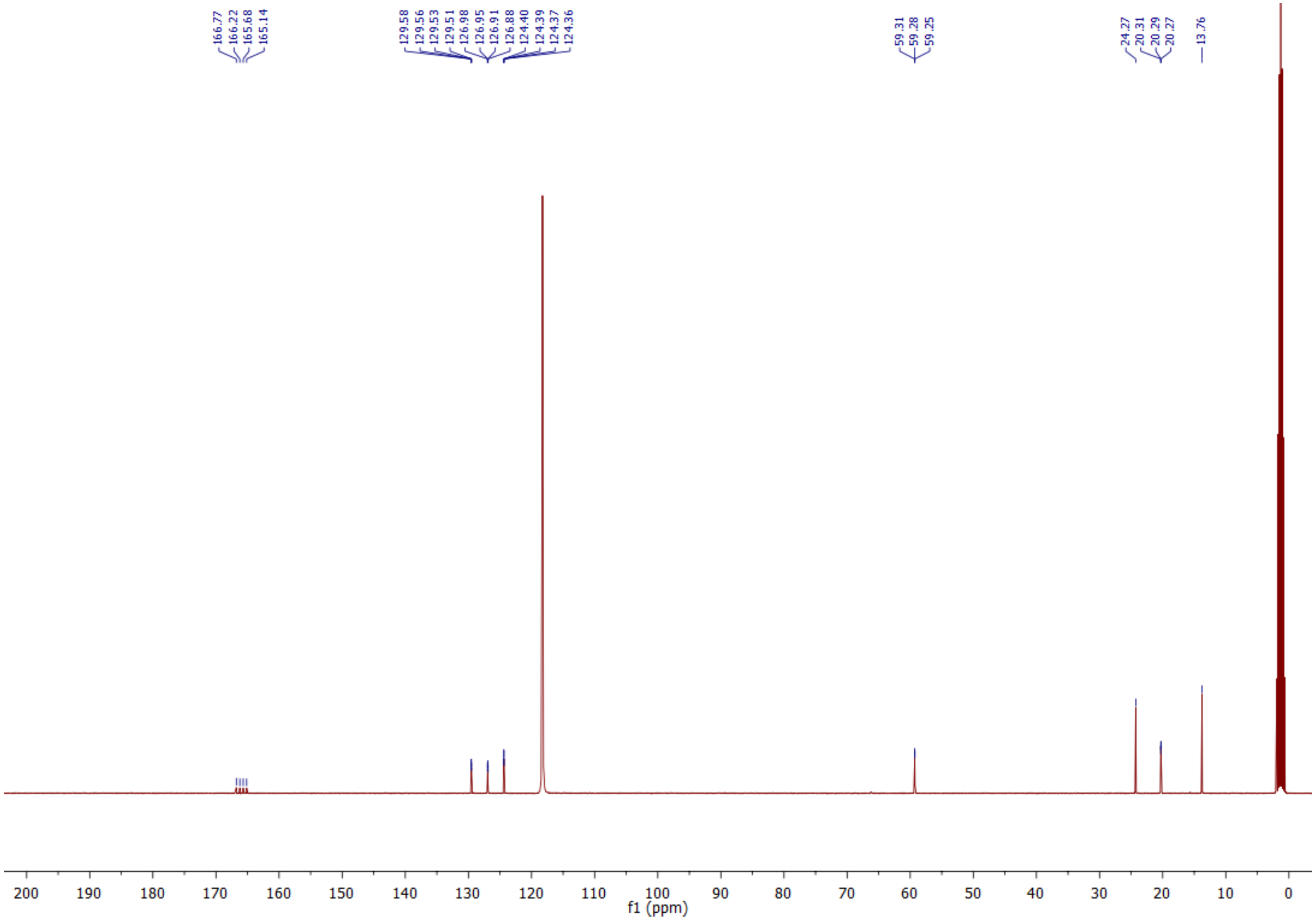

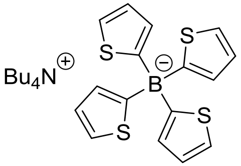

^{11}B NMR (CD_3CN, 128 MHz). Tetrabutylammonium tetra(thiophen-2-yl)borate (**3e**)

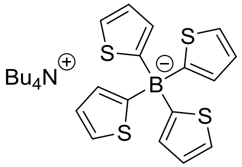

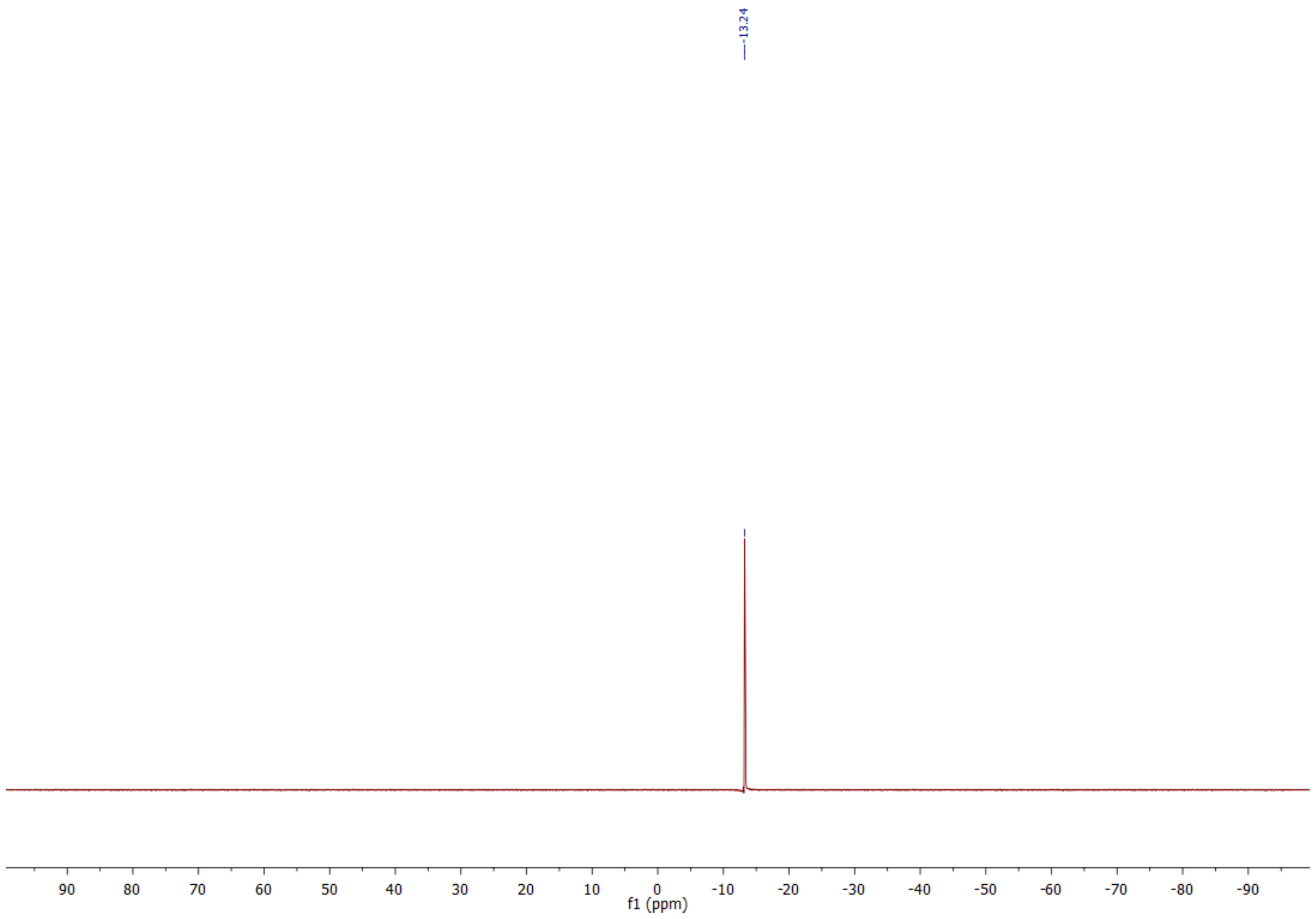

^{1}H NMR ($CDCl_3$, 400 MHz). 1-(4-(1*H*-Tetrazol-1-yl)phenyl)-2-diazoethan-1-one (**2f**)

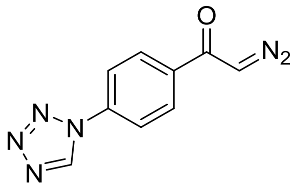

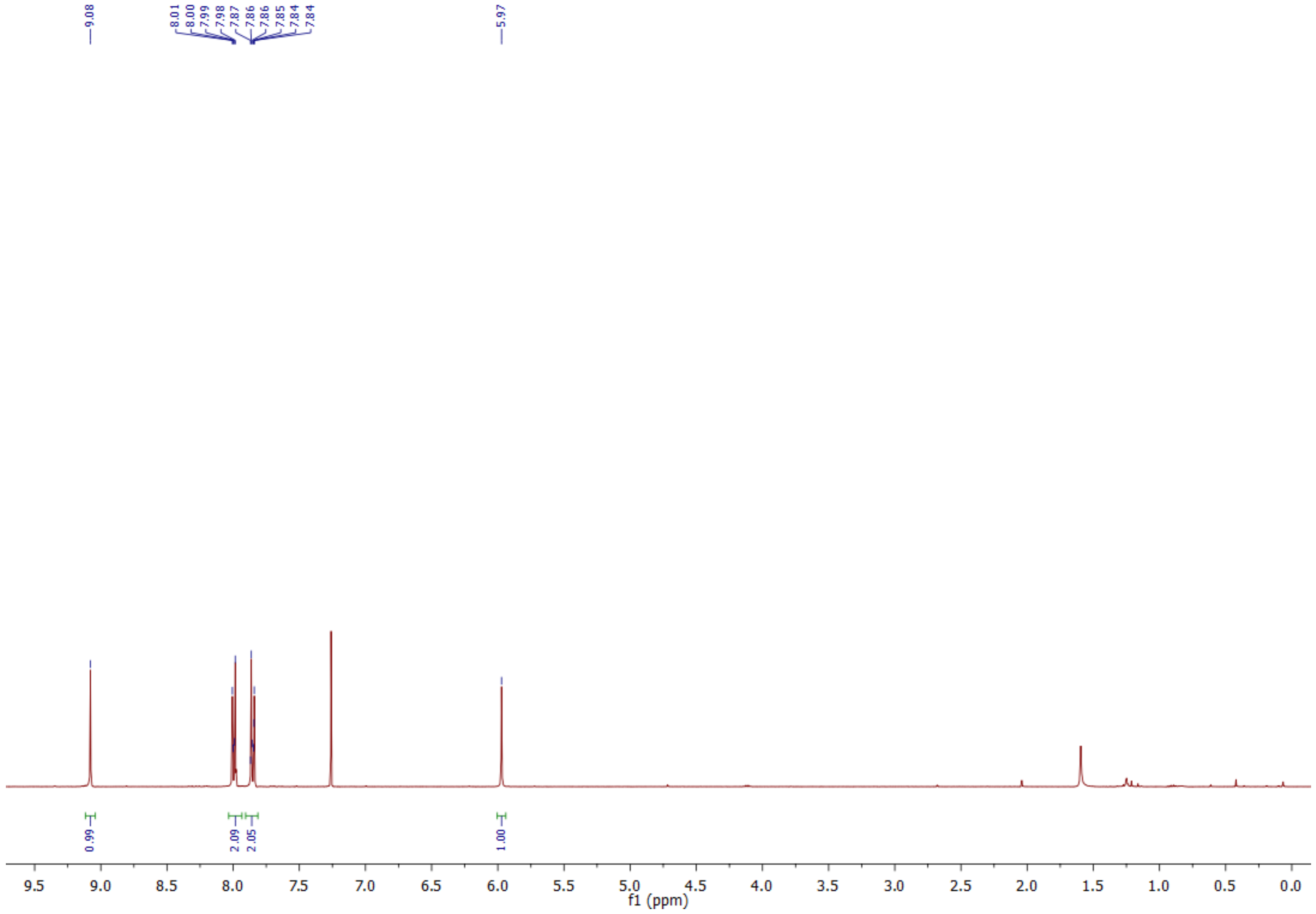

^{13}C NMR ($CDCl_3$, 100 MHz). 1-(4-(1*H*-Tetrazol-1-yl)phenyl)-2-diazoethan-1-one (**2f**)

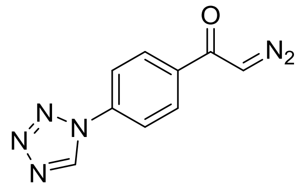

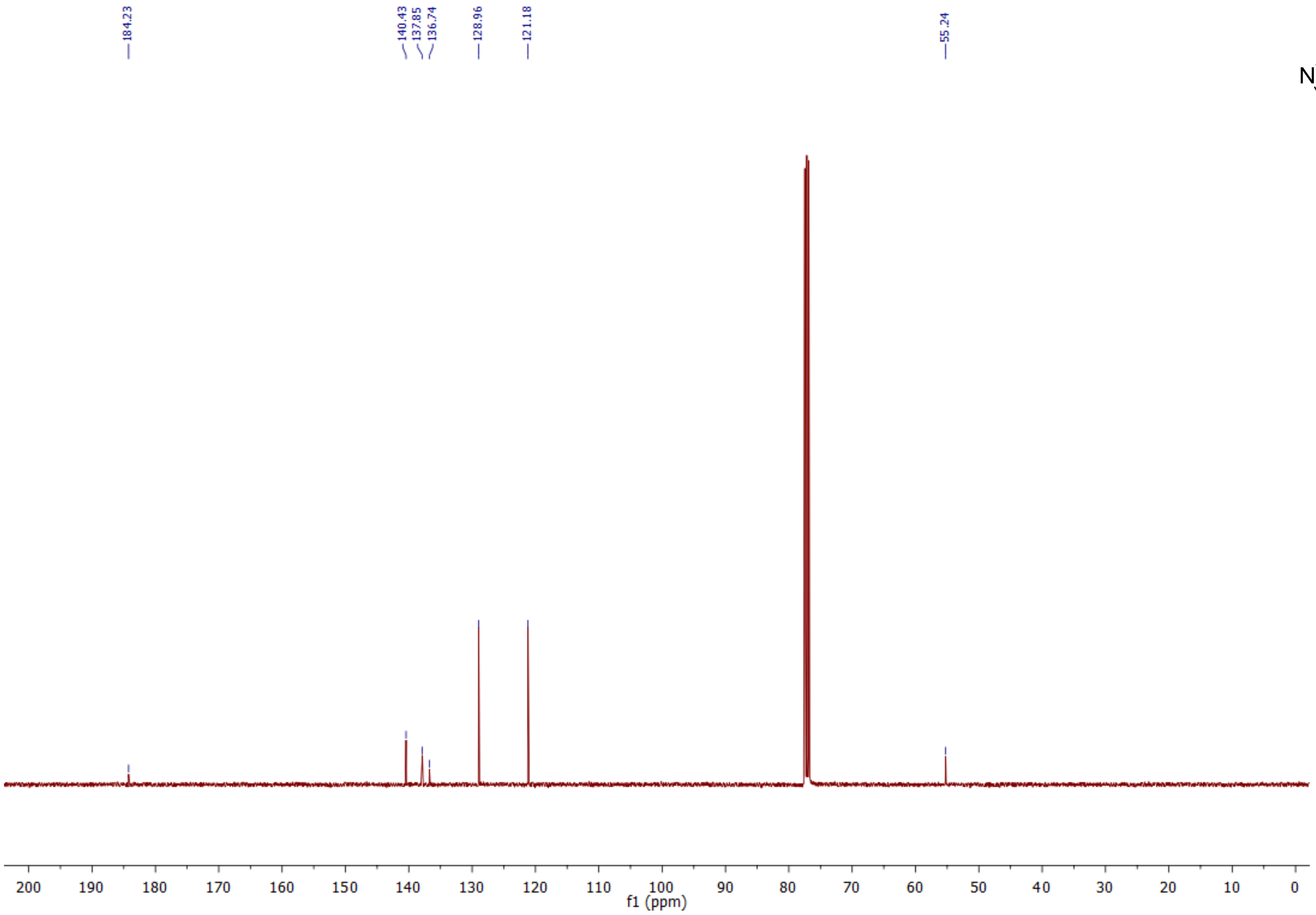

^1H NMR ($CDCl_3$, 400 MHz). 1,2-Diphenyl-2-((trifluoromethyl)thio)ethan-1-one (**5a**)

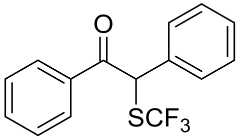

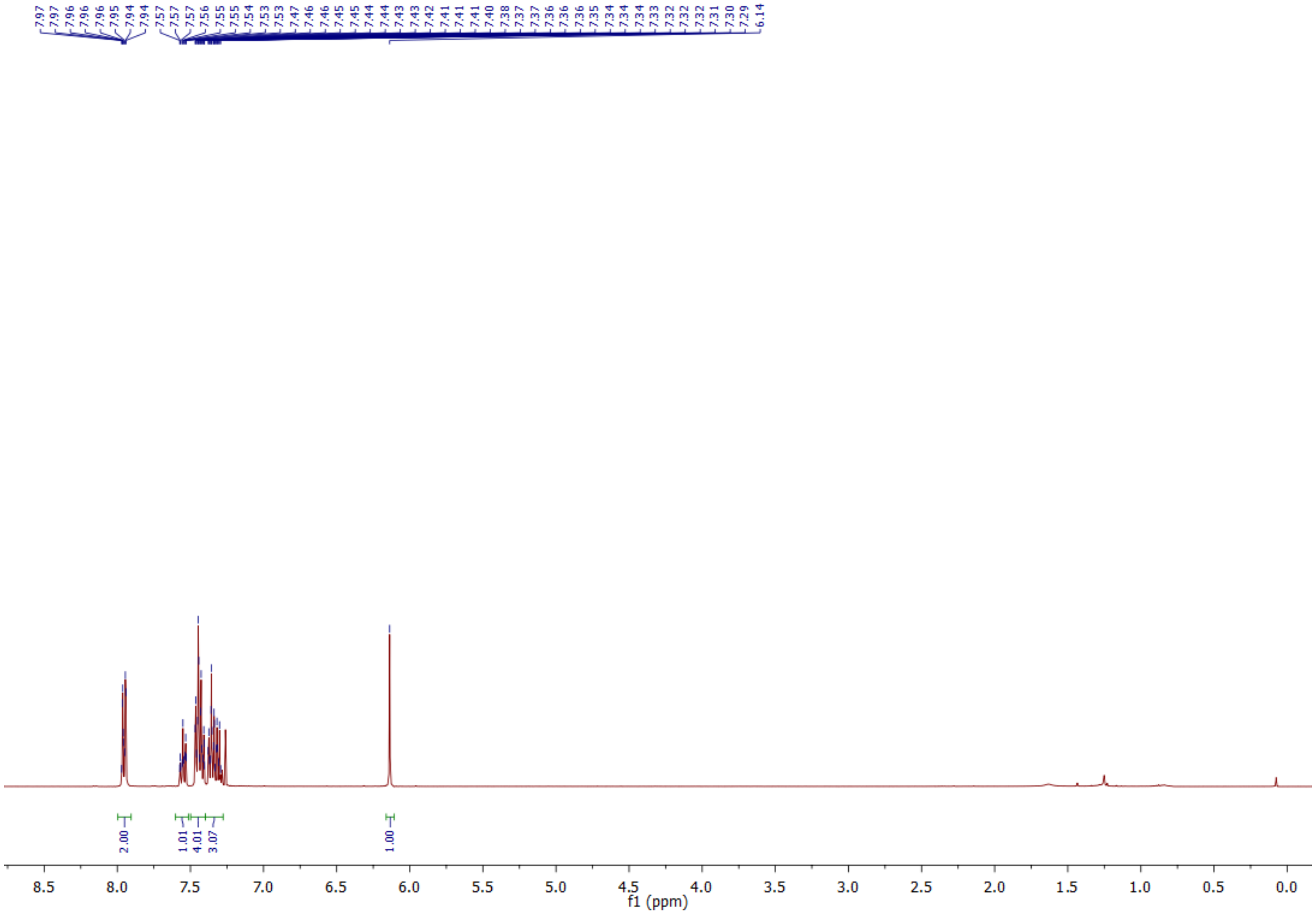

^{13}C NMR ($CDCl_3$, 100 MHz). 1,2-Diphenyl-2-((trifluoromethyl)thio)ethan-1-one (**5a**)

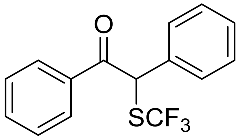

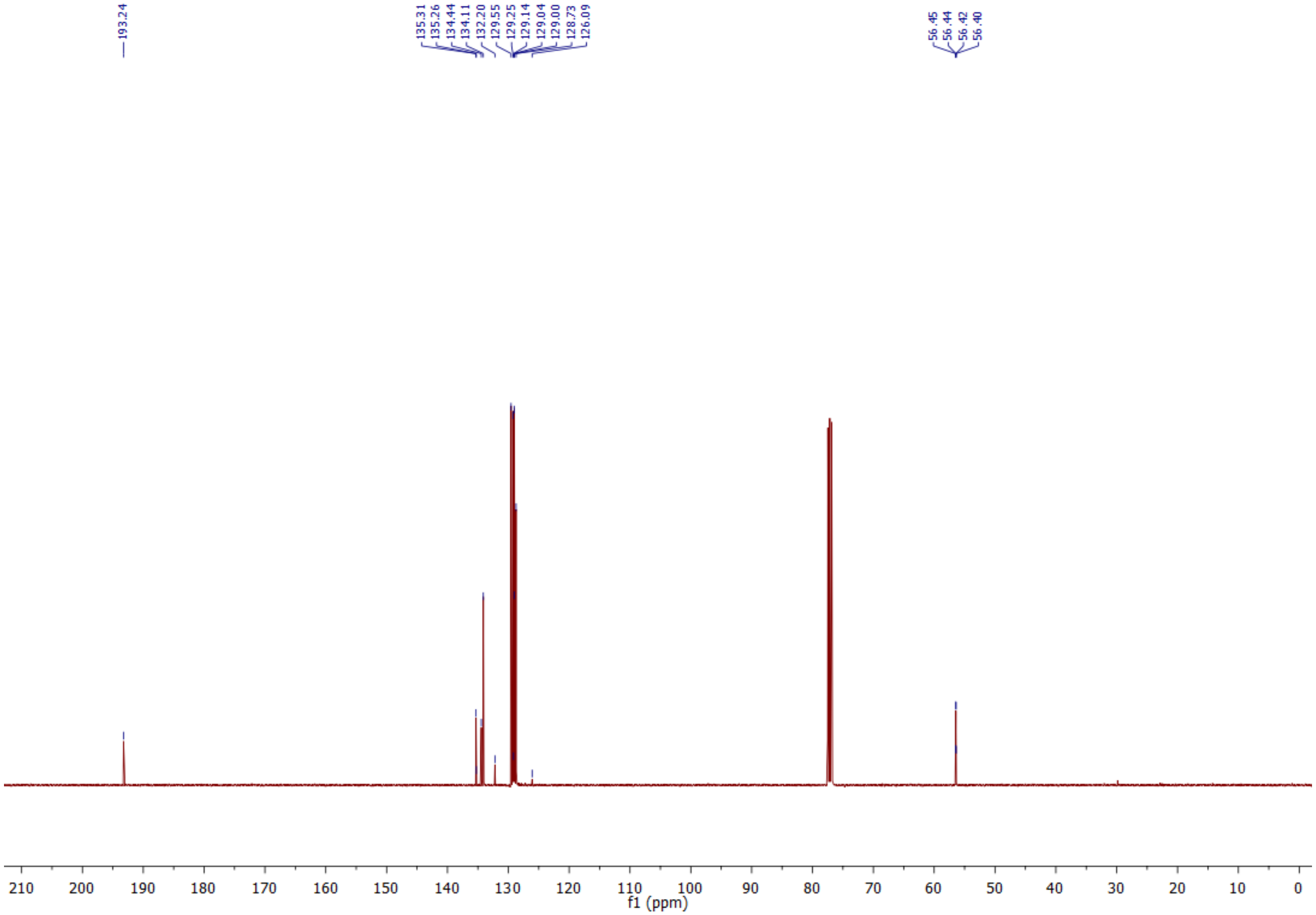

^{19}F NMR ($CDCl_3$, 377 MHz). 1,2-Diphenyl-2-((trifluoromethyl)thio)ethan-1-one (**5a**)

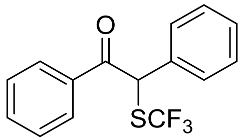

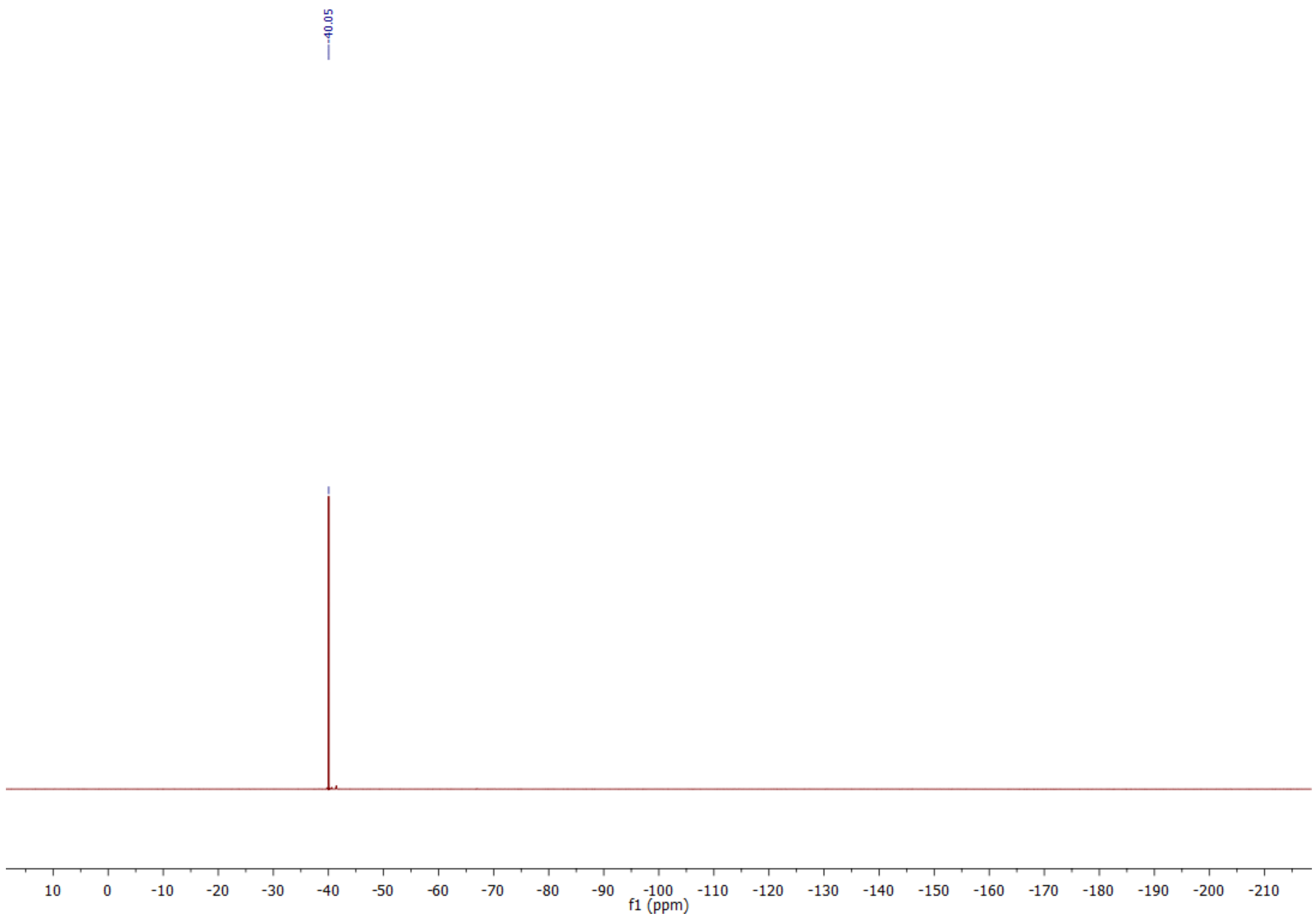

^{1}H NMR ($CDCl_3$, 400 MHz). 1-(4-Fluorophenyl)-2-phenyl-2-((trifluoromethyl)thio)ethan-1-one (**5b**)

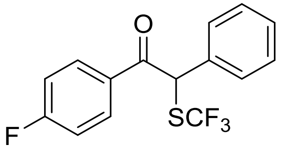

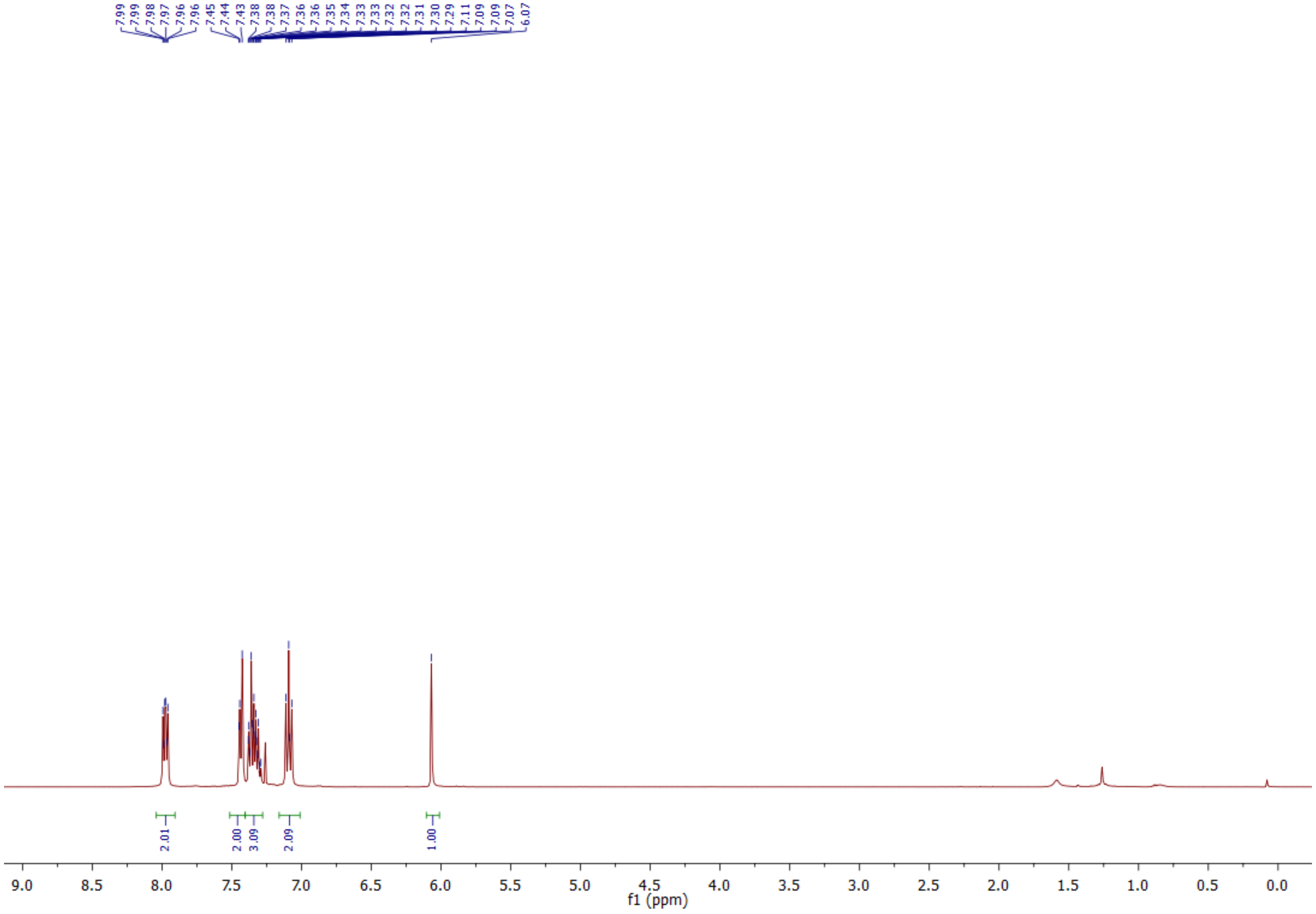

^{13}C NMR ($CDCl_3$, 100 MHz). 1-(4-Fluorophenyl)-2-phenyl-2-((trifluoromethyl)thio)ethan-1-one (**5b**)

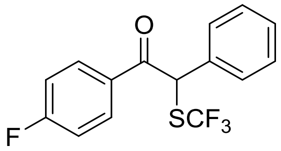

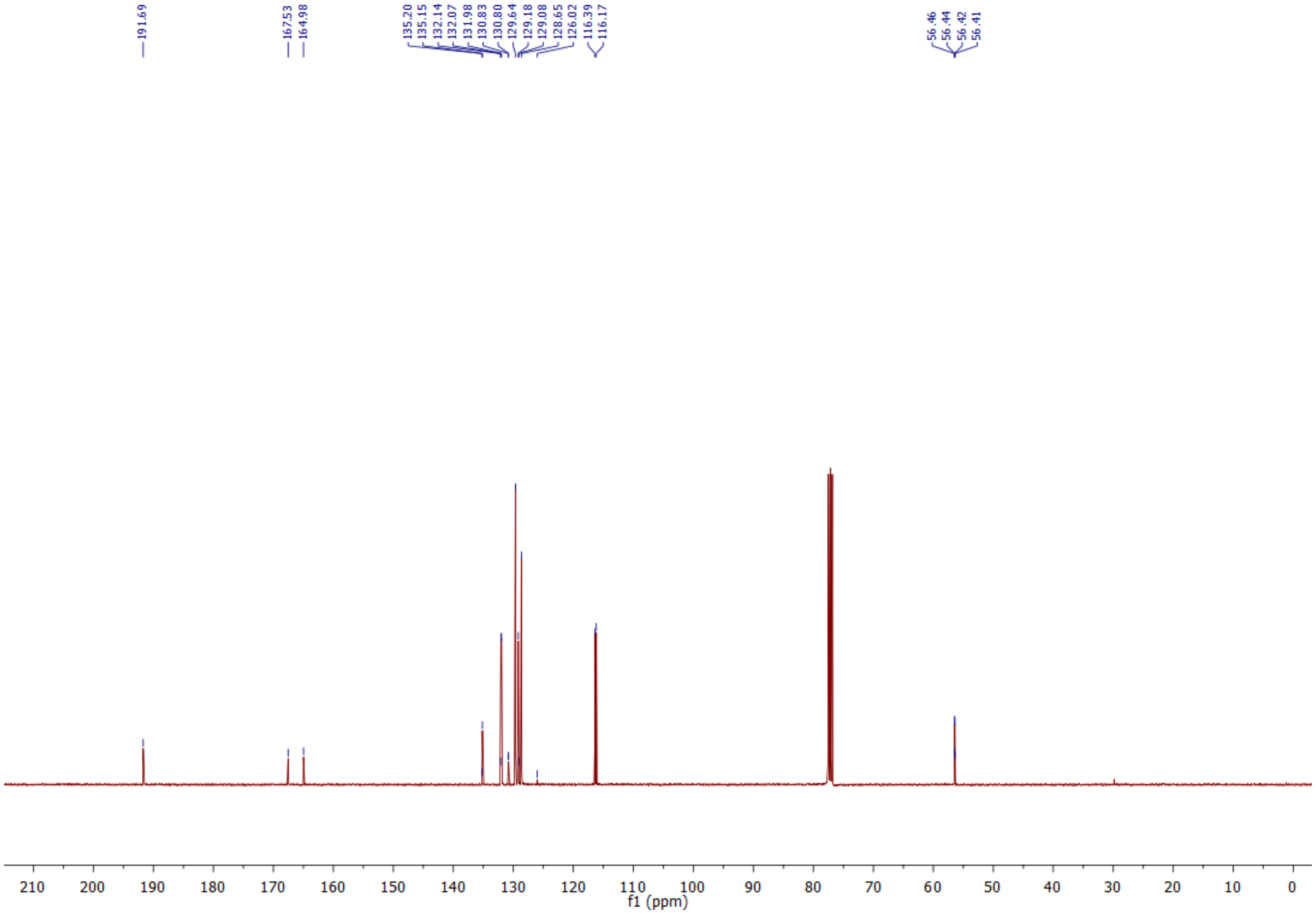

^{19}F NMR ($CDCl_3$, 377 MHz). 1-(4-Fluorophenyl)-2-phenyl-2-((trifluoromethyl)thio)ethan-1-one (**5b**)

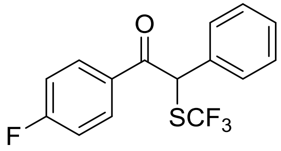

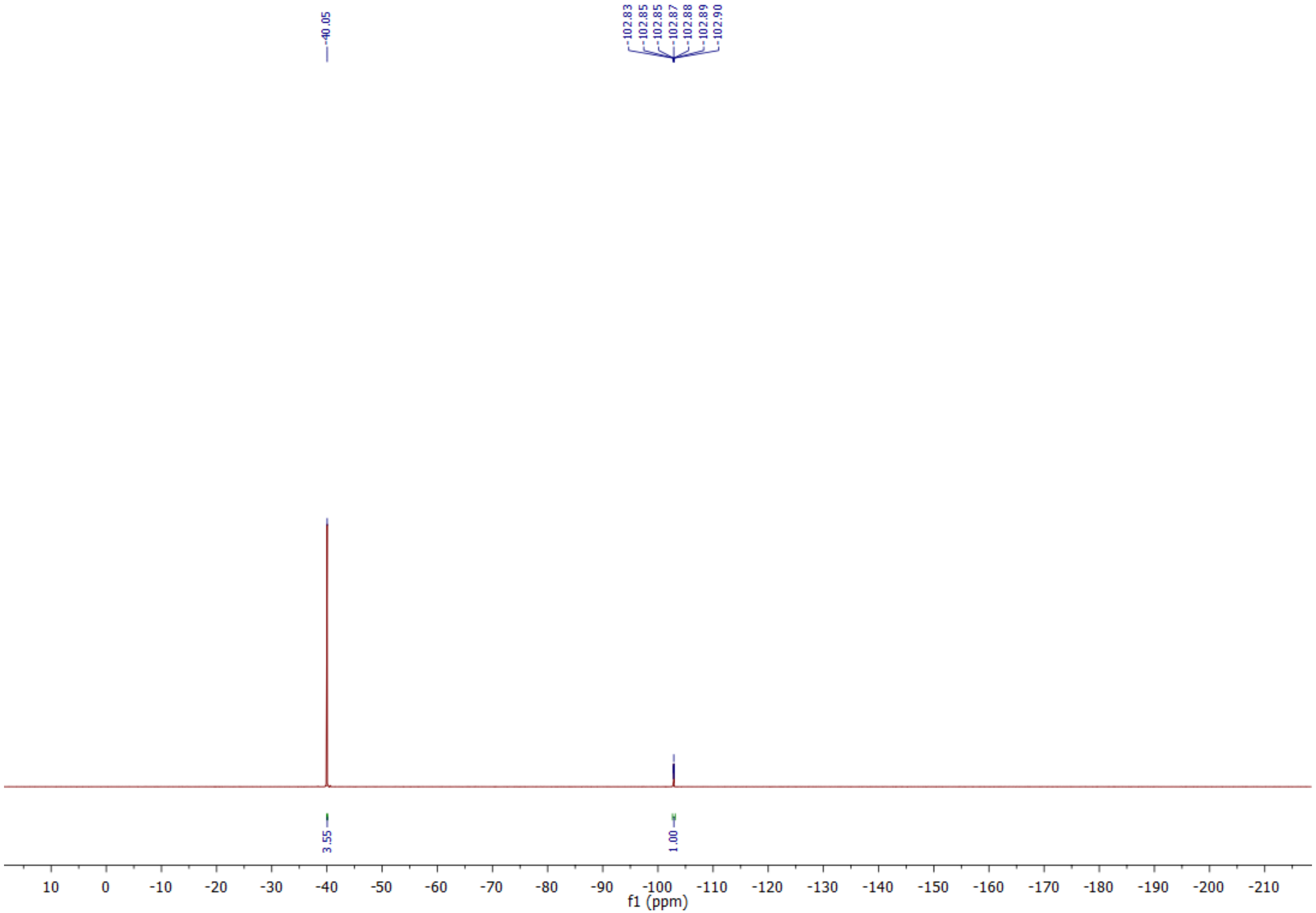

^{1}H NMR ($CDCl_3$, 400 MHz). 1-(2-Iodophenyl)-2-phenyl-2-((trifluoromethyl)thio)ethan-1-one (**5c**)

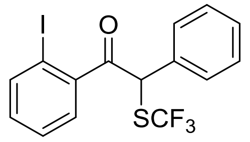

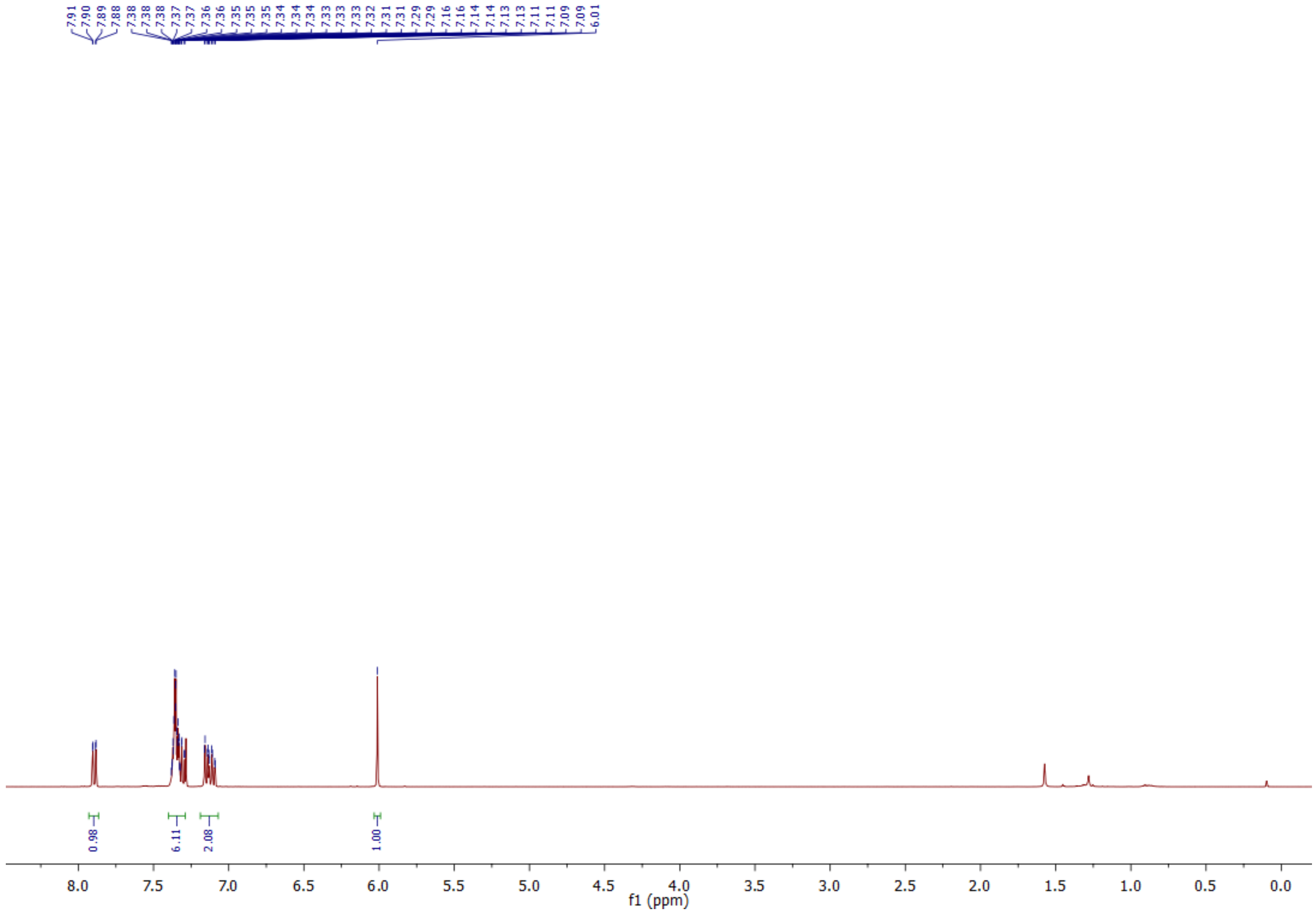

^{13}C NMR ($CDCl_3$, 100 MHz). 1-(2-Iodophenyl)-2-phenyl-2-((trifluoromethyl)thio)ethan-1-one (**5c**)

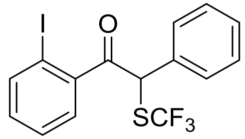

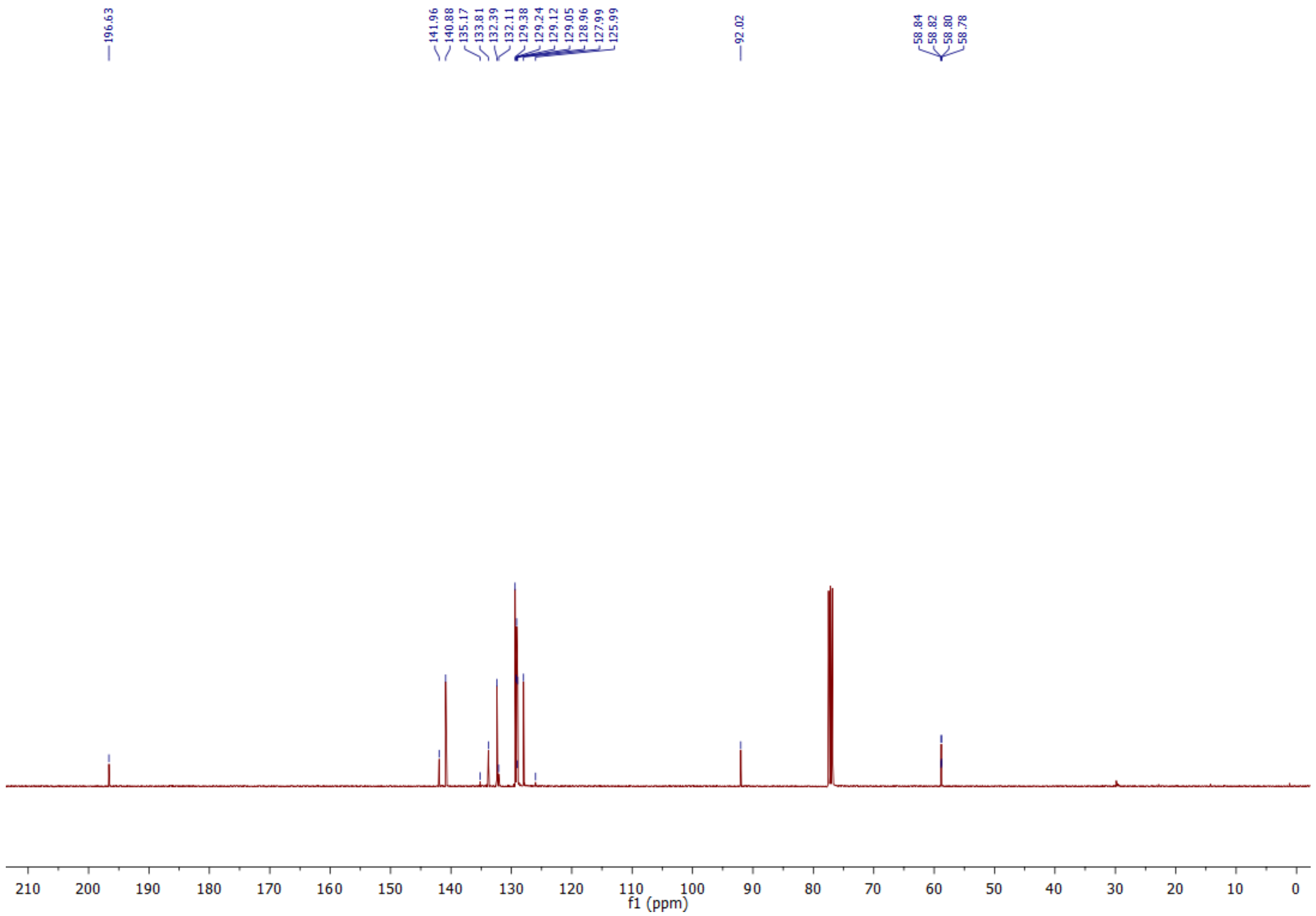

^{19}F NMR ($CDCl_3$, 377 MHz). 1-(2-Iodophenyl)-2-phenyl-2-((trifluoromethyl)thio)ethan-1-one (**5c**)

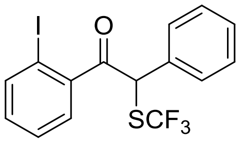

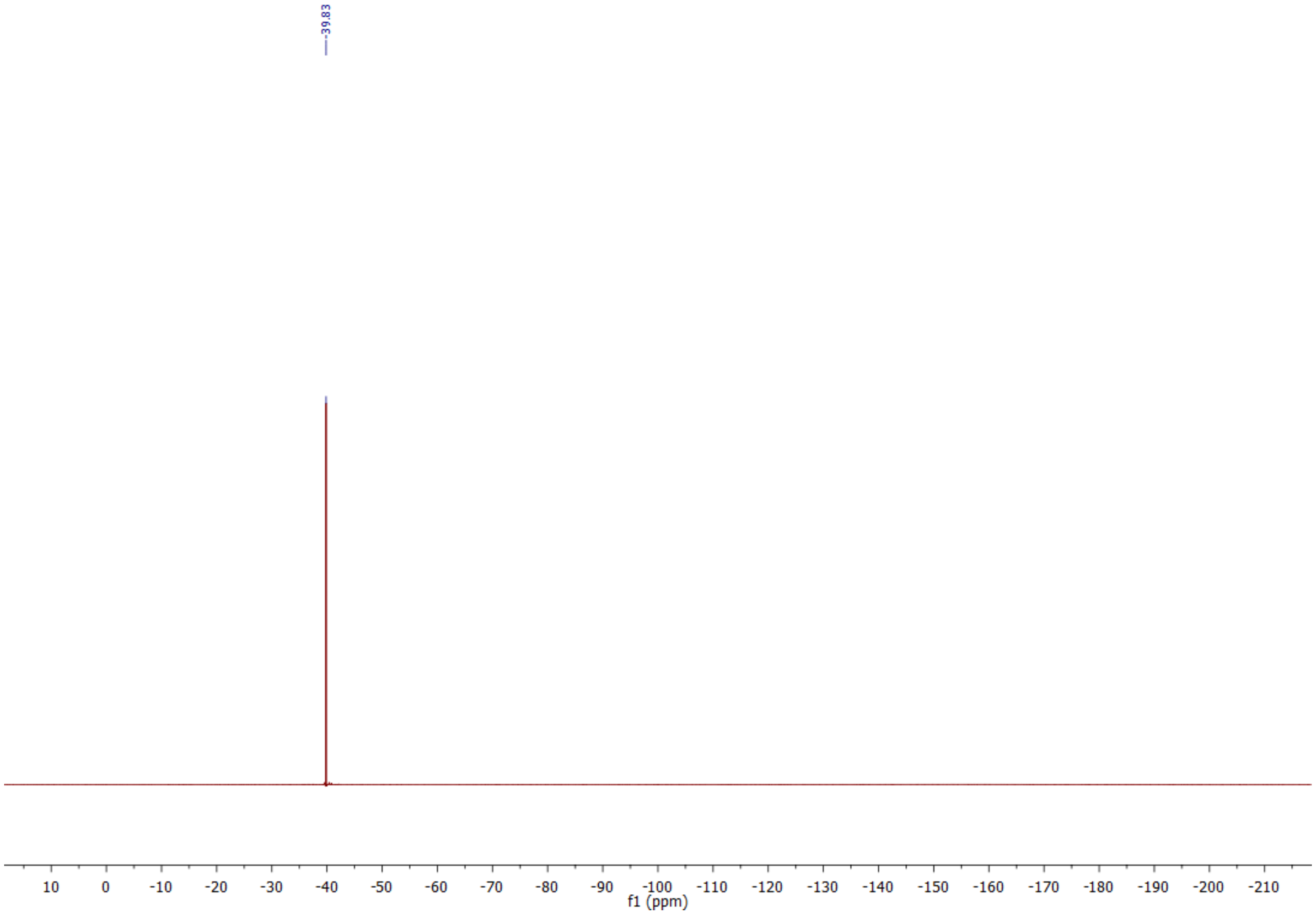

^{1}H NMR ($CDCl_3$, 400 MHz). 1-(4-Nitrophenyl)-2-phenyl-2-((trifluoromethyl)thio)ethan-1-one (**5d**)

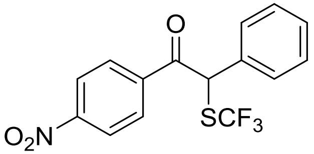

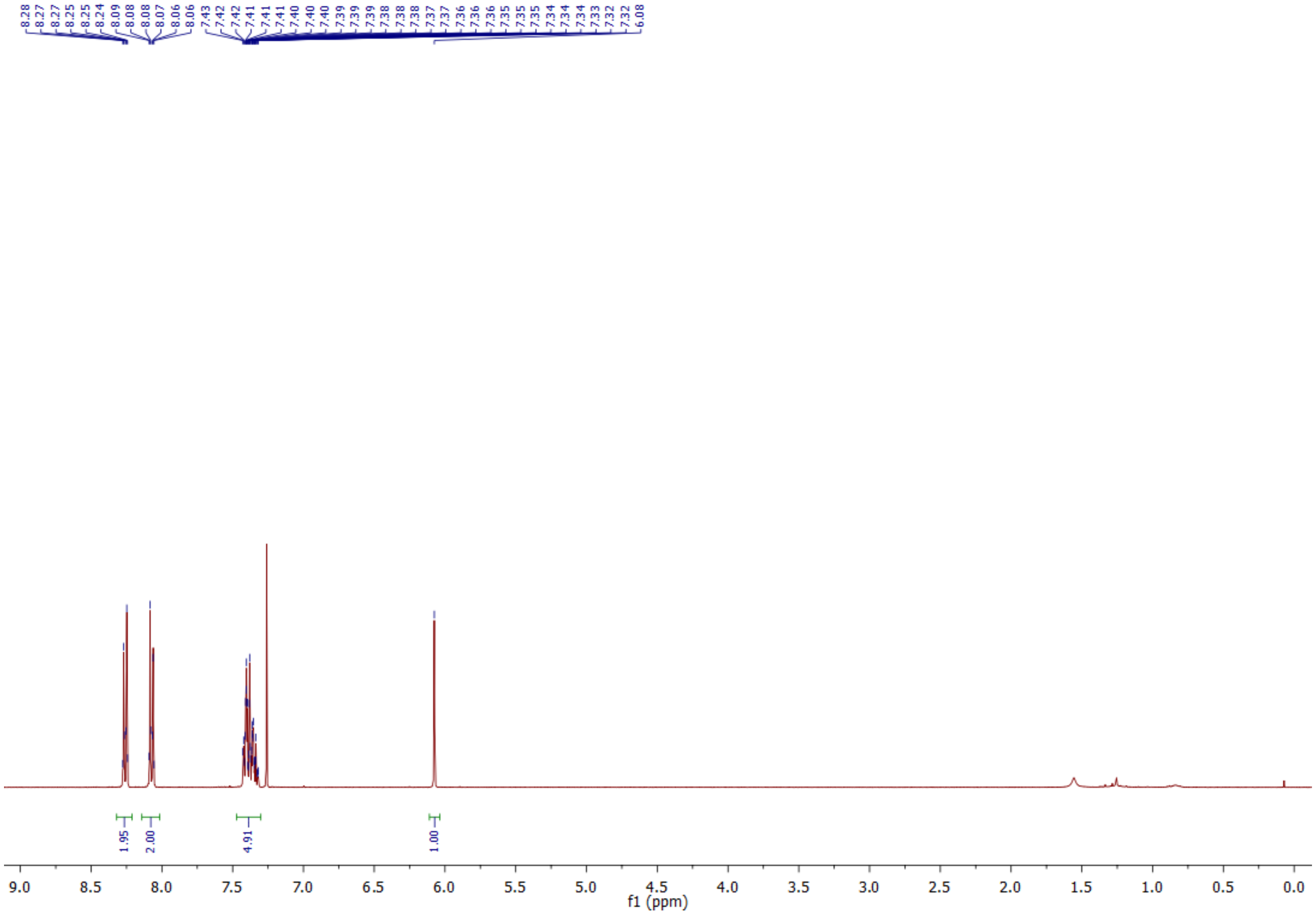

^{13}C NMR ($CDCl_3$, 100 MHz). 1-(4-Nitrophenyl)-2-phenyl-2-((trifluoromethyl)thio)ethan-1-one (**5d**)

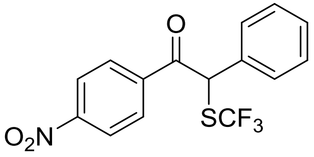

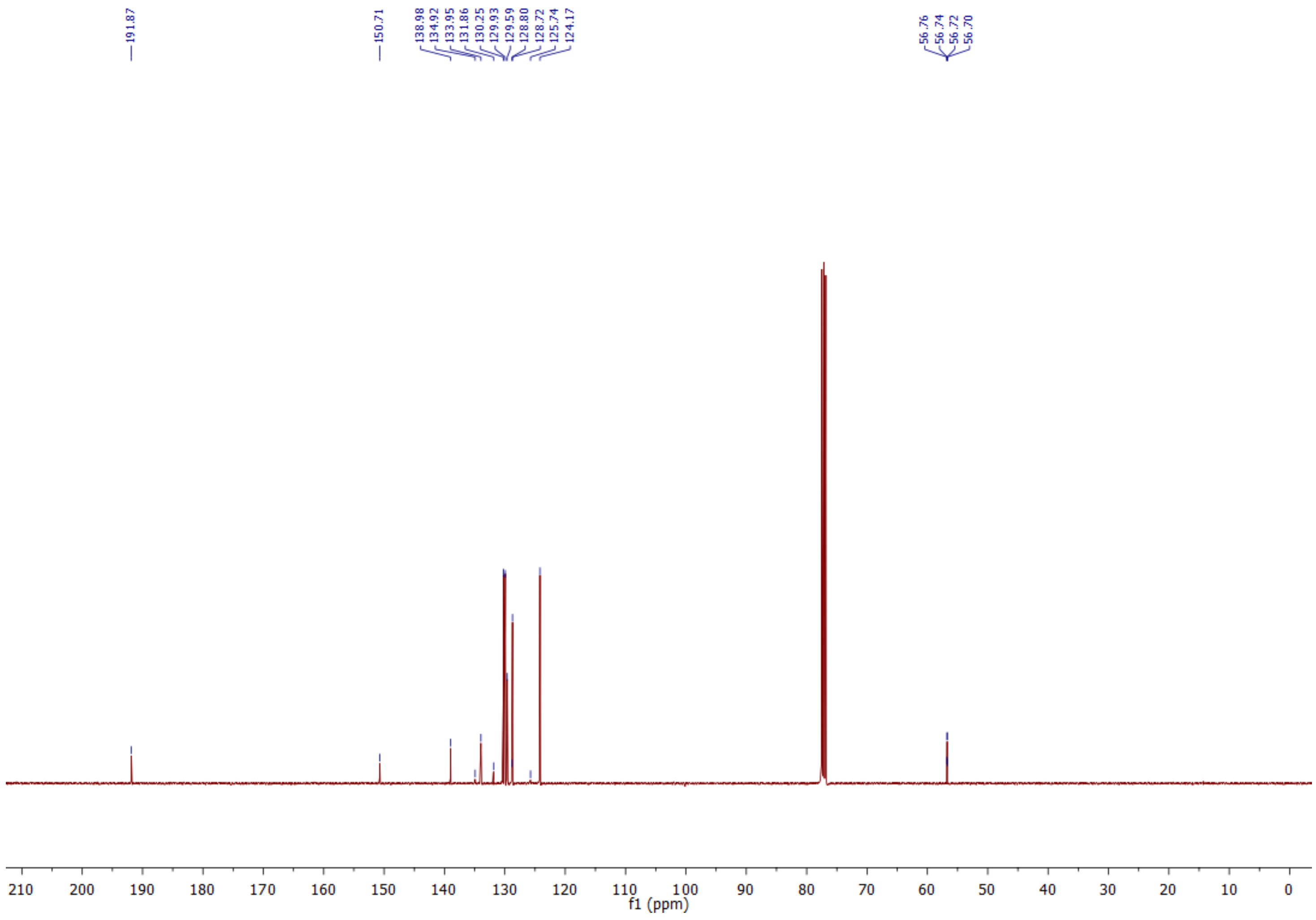

^{19}F NMR ($CDCl_3$, 377 MHz). 1-(4-Nitrophenyl)-2-phenyl-2-((trifluoromethyl)thio)ethan-1-one (**5d**)

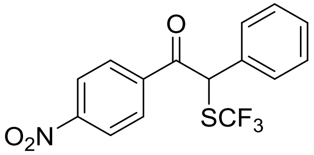

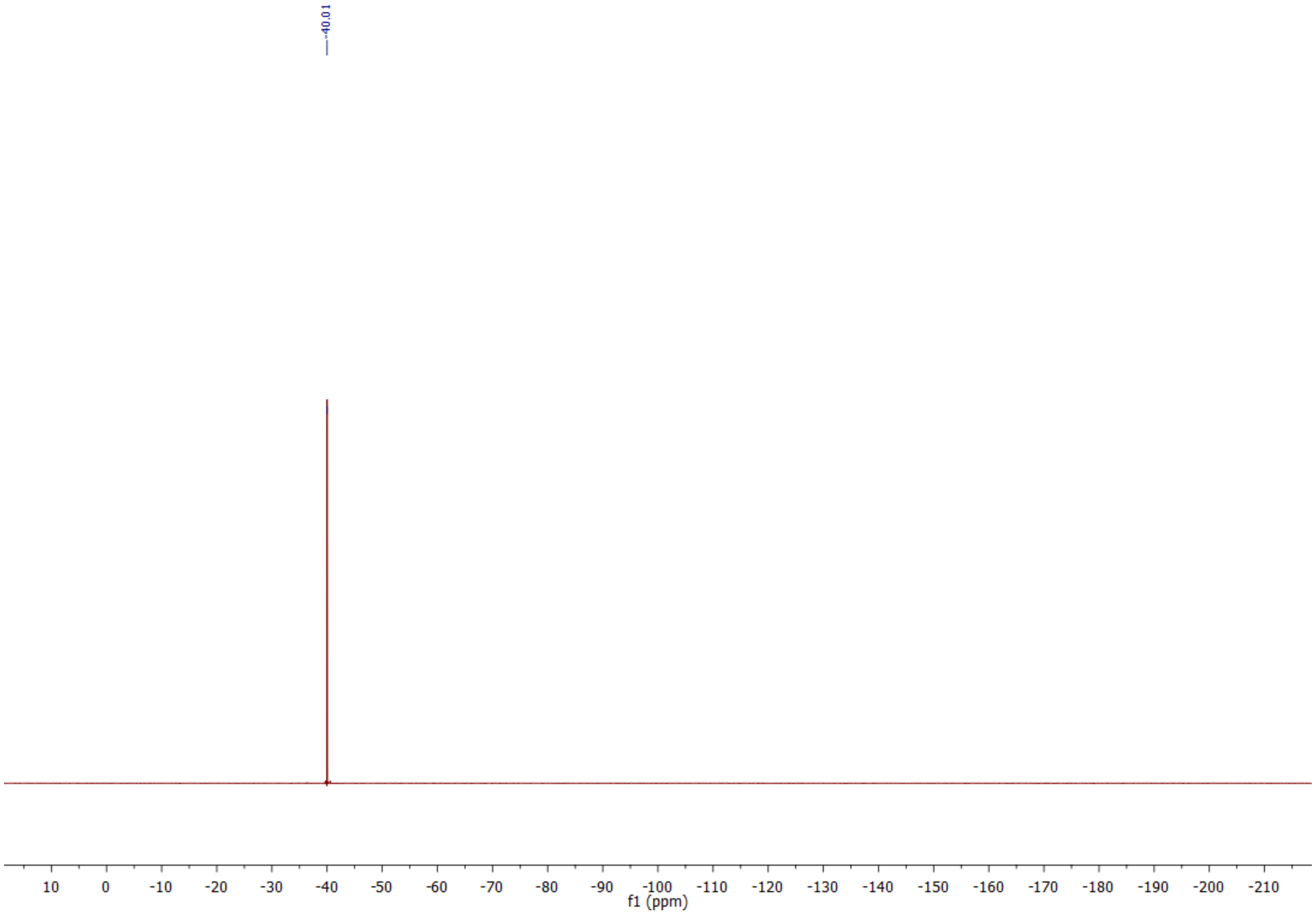

^{1}H NMR ($CDCl_3$, 400 MHz). 1-(4-Methoxyphenyl)-2-phenyl-2-((trifluoromethyl)thio)ethan-1-one (**5e**)

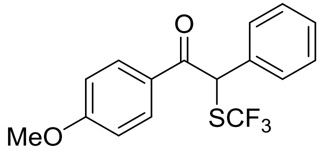

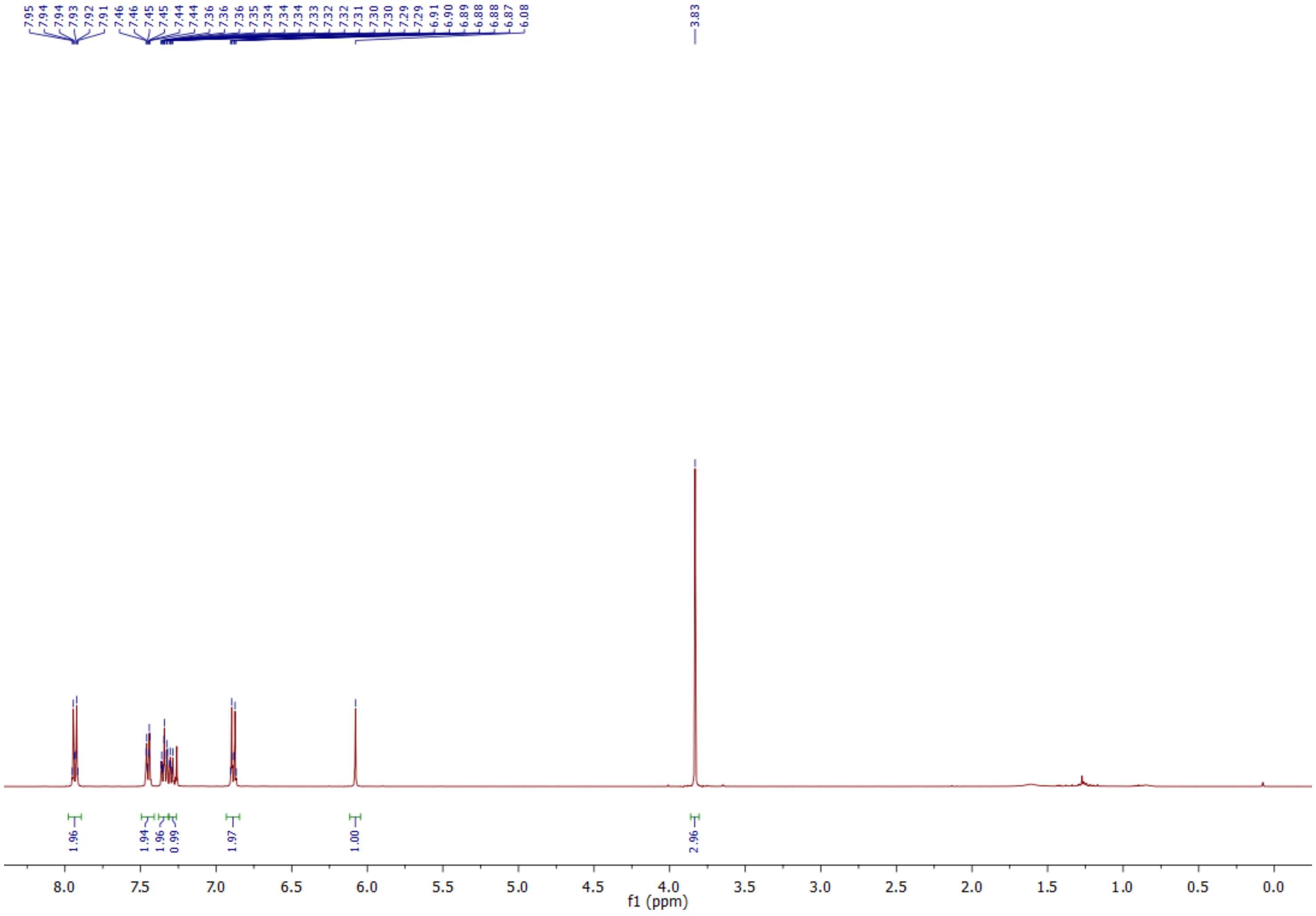

^{13}C NMR ($CDCl_3$, 100 MHz). 1-(4-Methoxyphenyl)-2-phenyl-2-((trifluoromethyl)thio)ethan-1-one (**5e**)

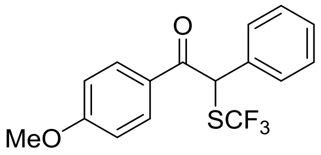

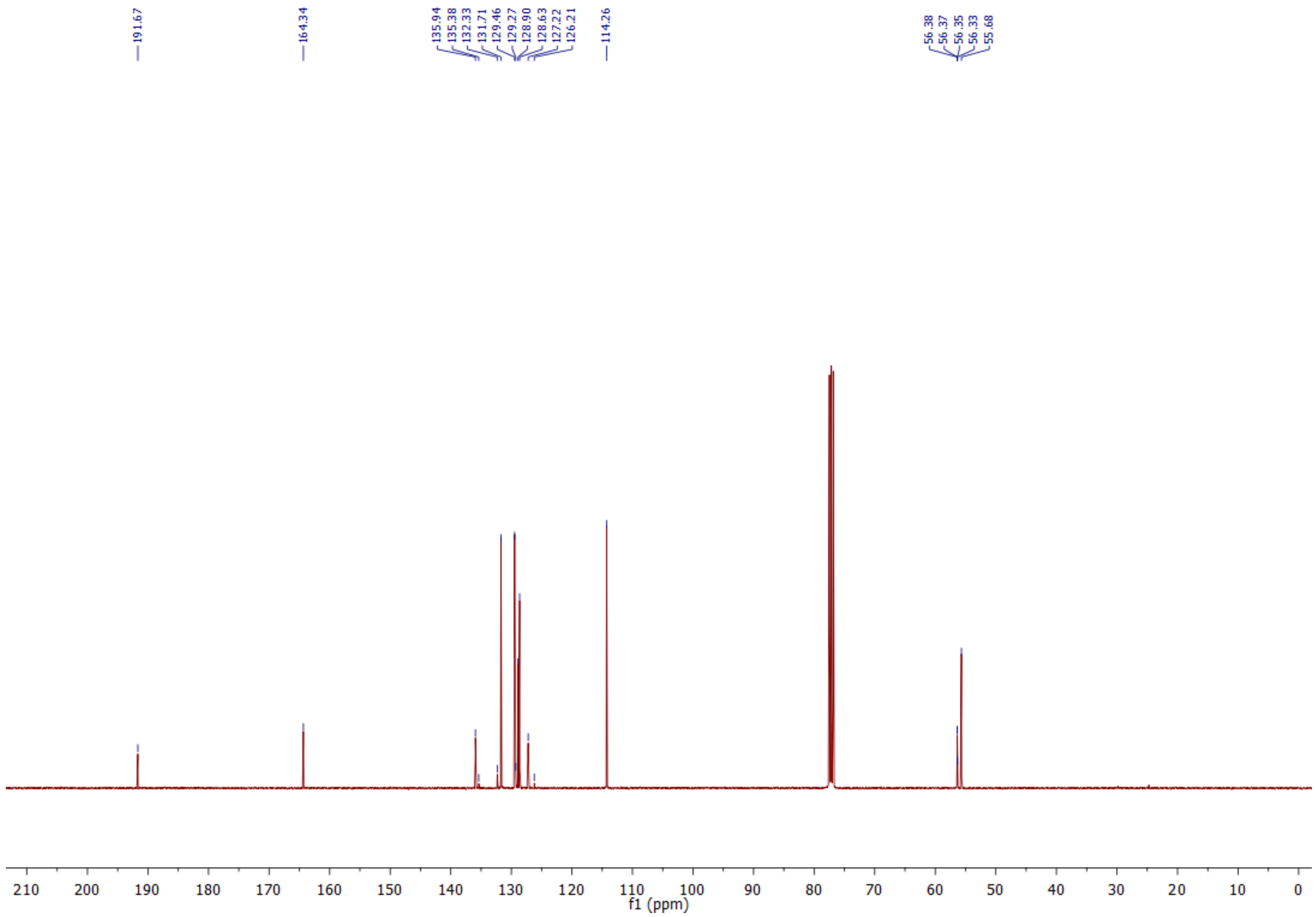

^{19}F NMR ($CDCl_3$, 377 MHz). 1-(4-Methoxyphenyl)-2-phenyl-2-((trifluoromethyl)thio)ethan-1-one (**5e**)

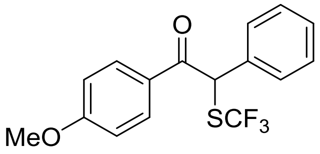

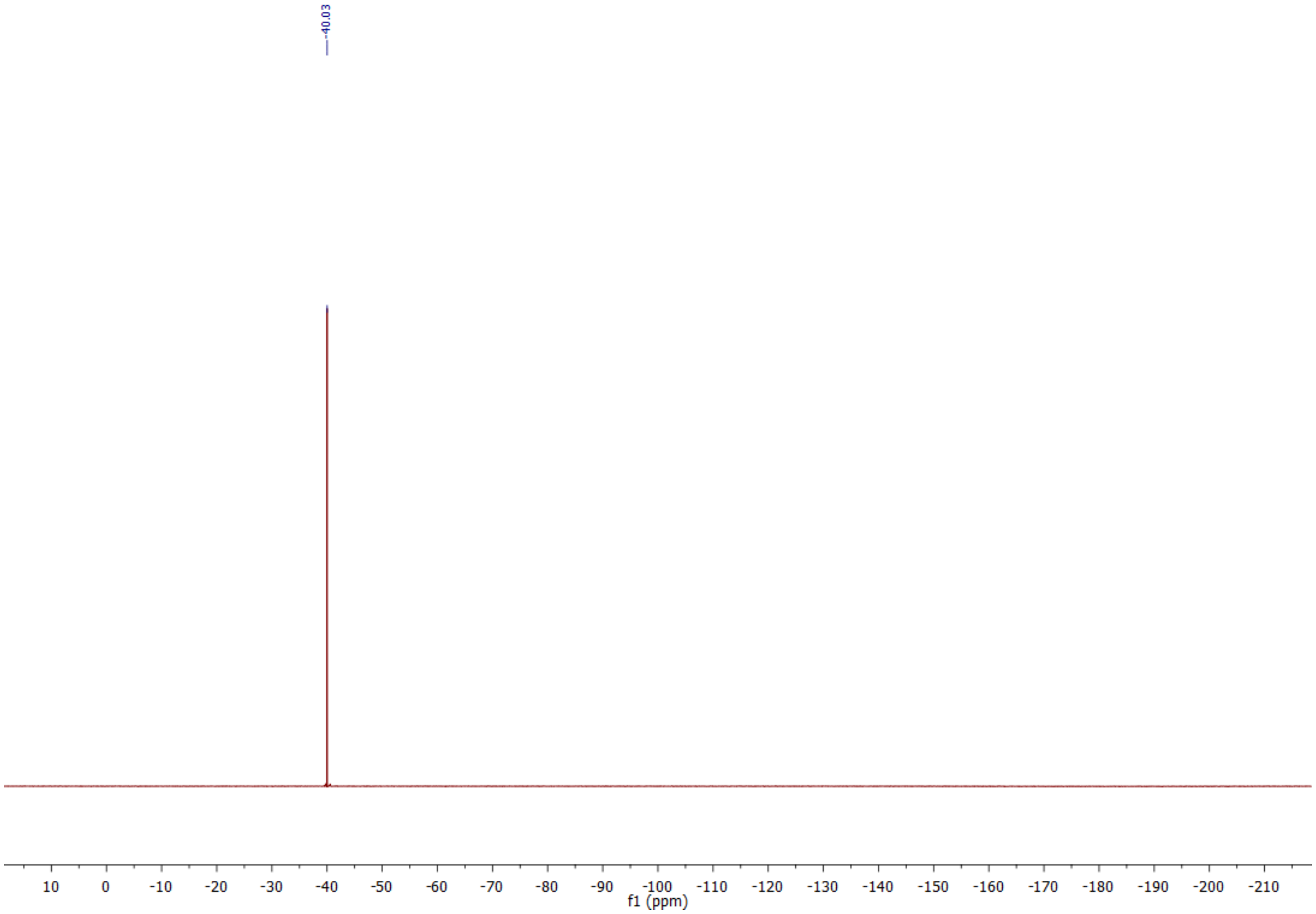

^{1}H NMR ($CDCl_3$, 400 MHz). 1-(4-(1*H*-Tetrazol-1-yl)phenyl)-2-phenyl-2-((trifluoromethyl)thio)ethan-1-one (**5f**)

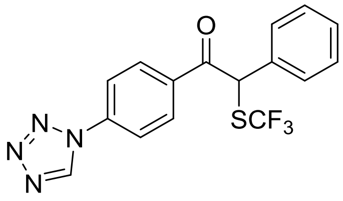

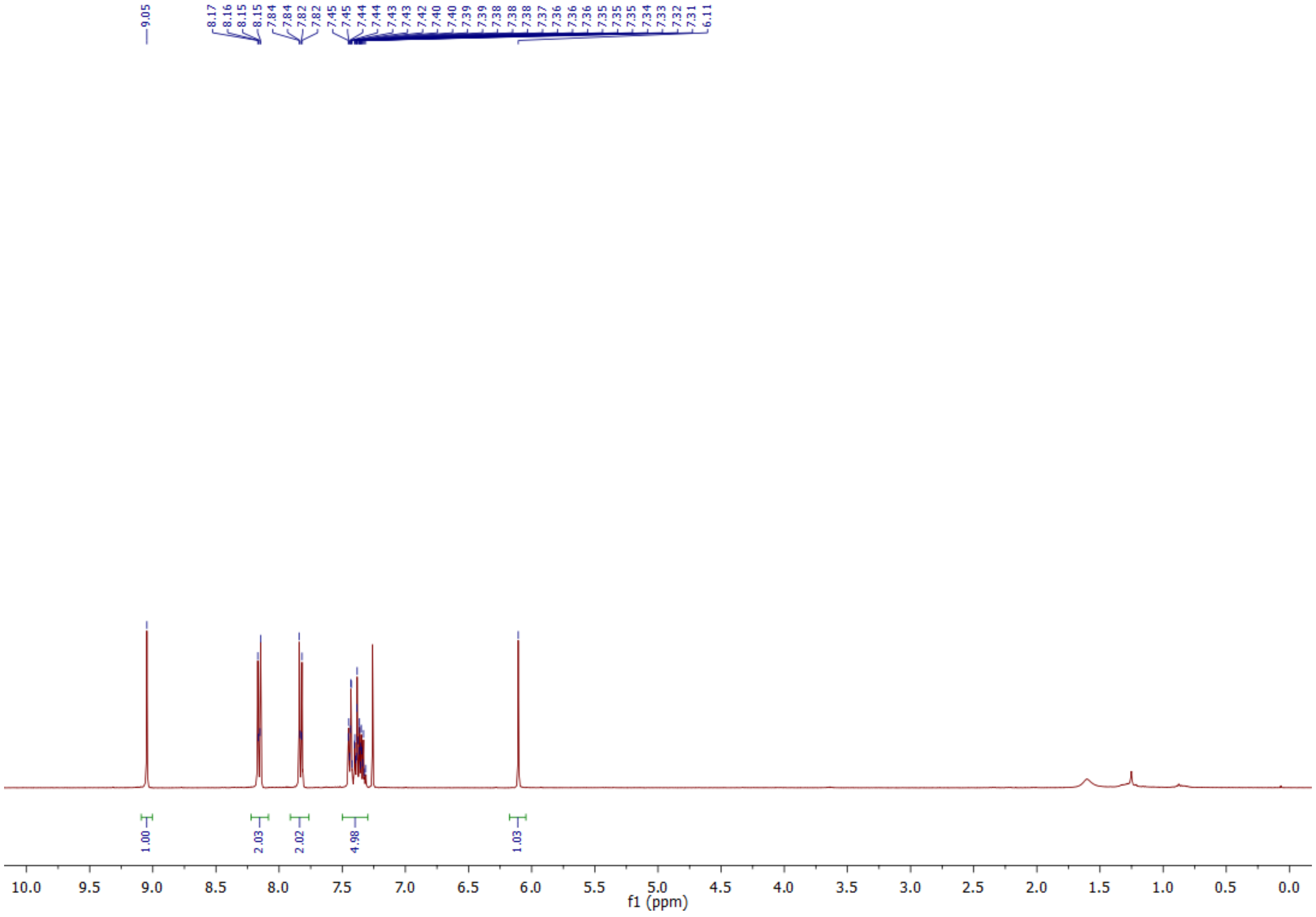

^{13}C NMR ($CDCl_3$, 100 MHz). 1-(4-(1*H*-Tetrazol-1-yl)phenyl)-2-phenyl-2-((trifluoromethyl)thio)ethan-1-one (**5f**)

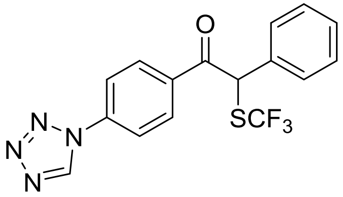

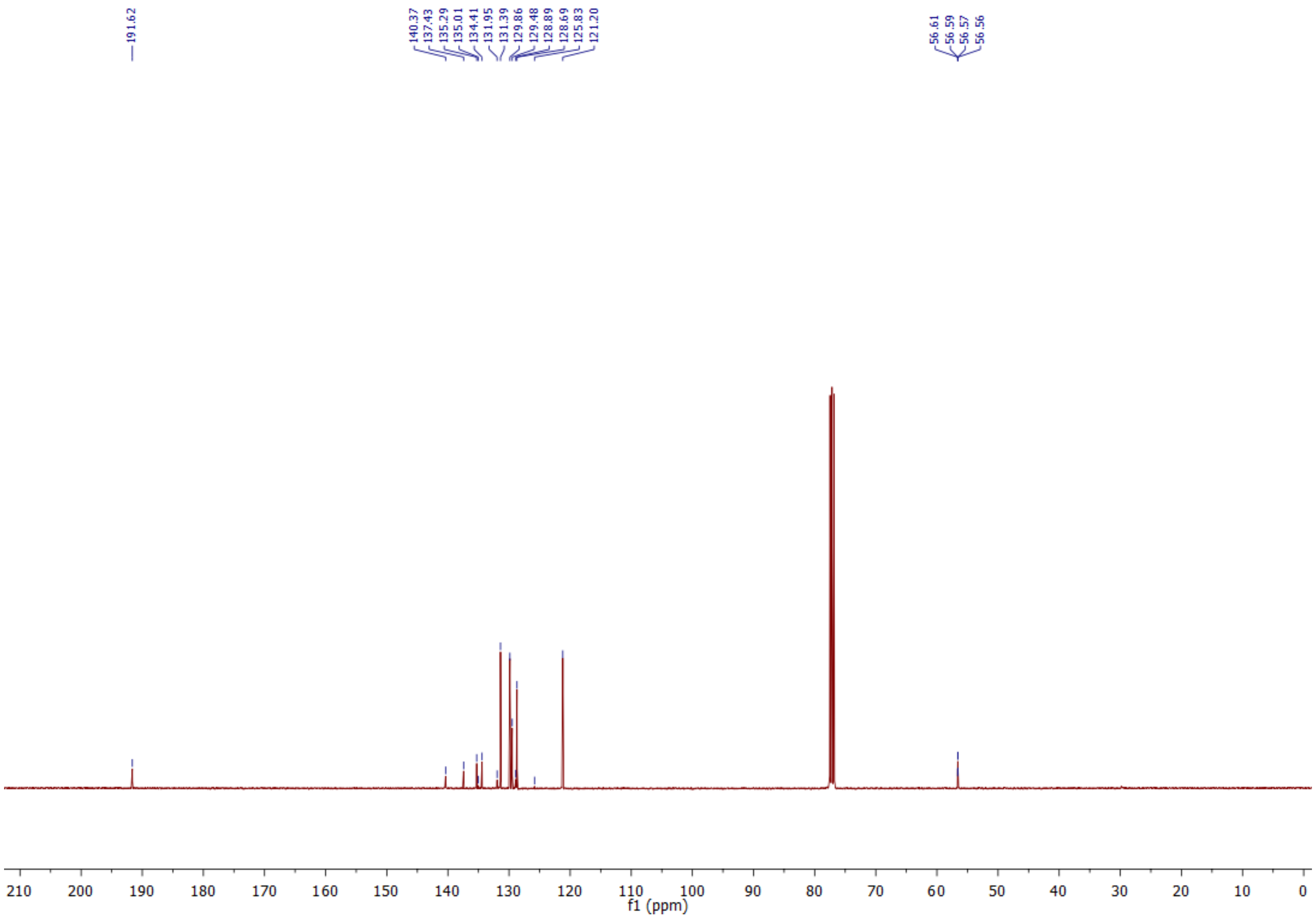

^{19}F NMR ($CDCl_3$, 377 MHz). 1-(4-(1*H*-Tetrazol-1-yl)phenyl)-2-phenyl-2-((trifluoromethyl)thio)ethan-1-one (**5f**)

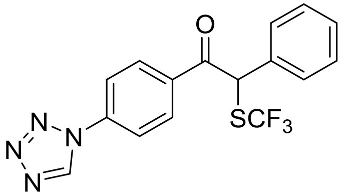

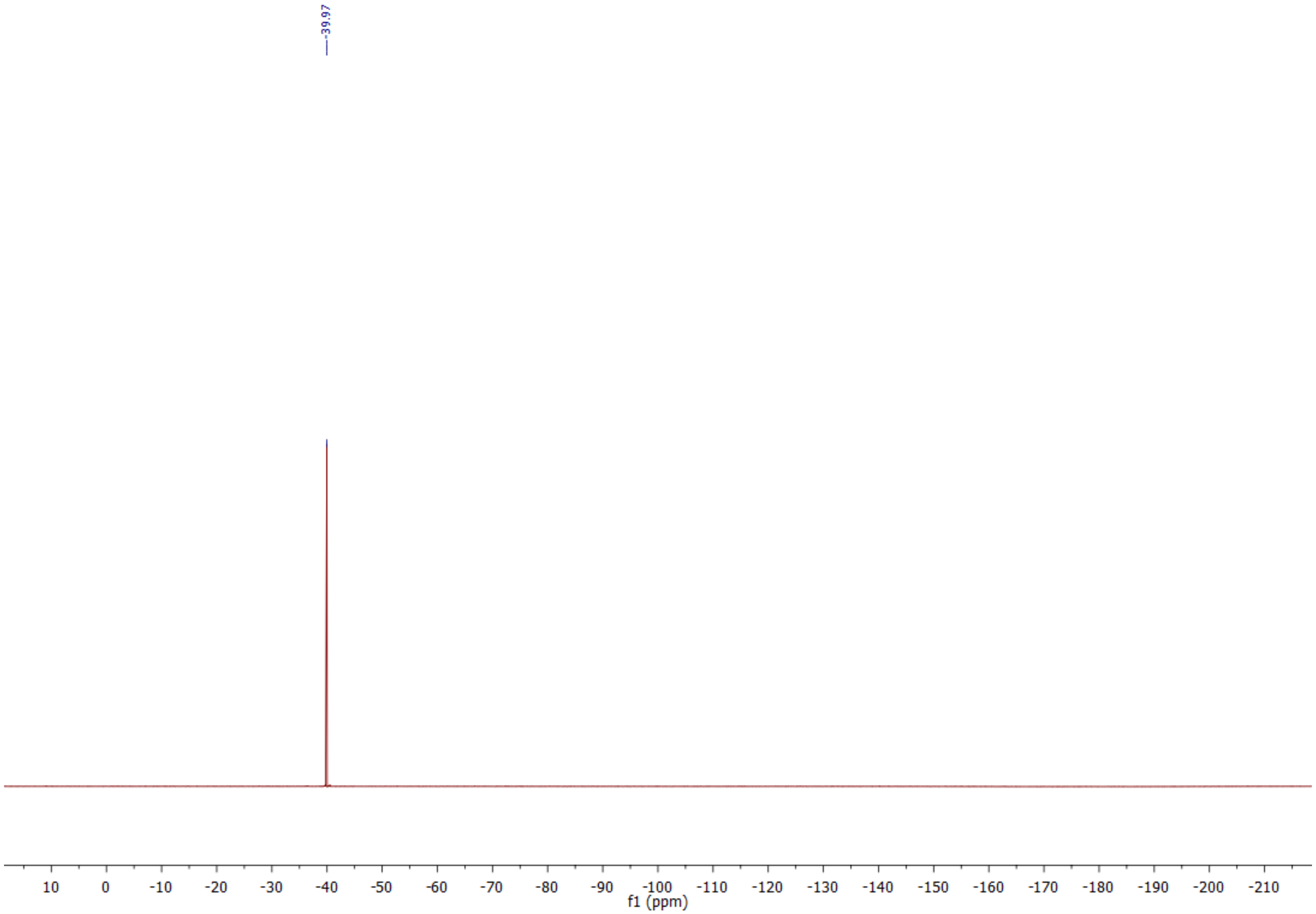

^1H NMR ($CDCl_3$, 400 MHz). 1-(Furan-2-yl)-2-phenyl-2-((trifluoromethyl)thio)ethan-1-one (**5g**)

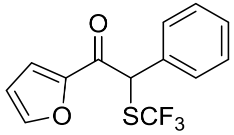

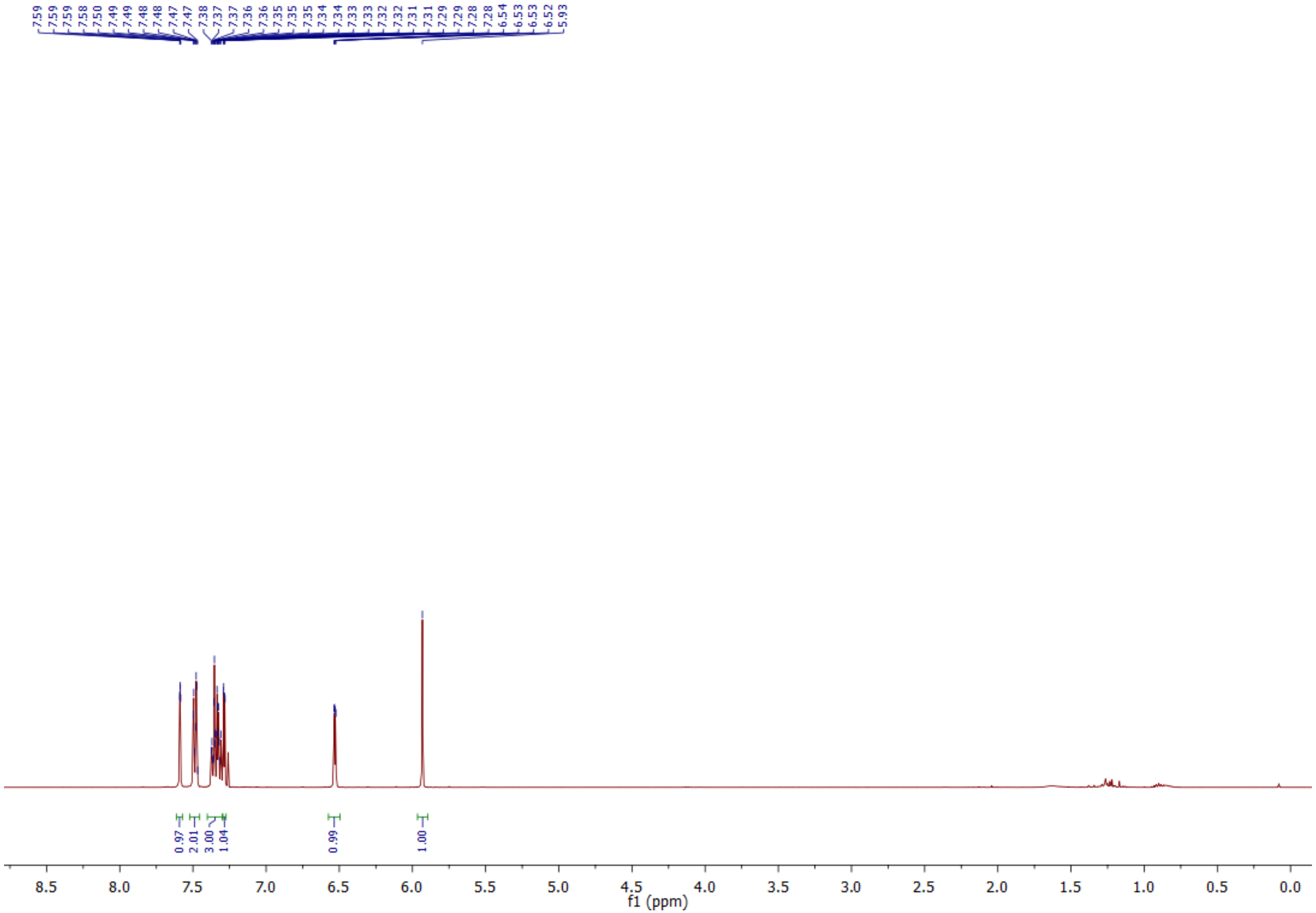

^{13}C NMR ($CDCl_3$, 100 MHz). 1-(Furan-2-yl)-2-phenyl-2-((trifluoromethyl)thio)ethan-1-one (**5g**)

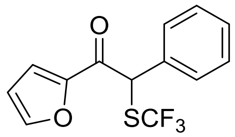

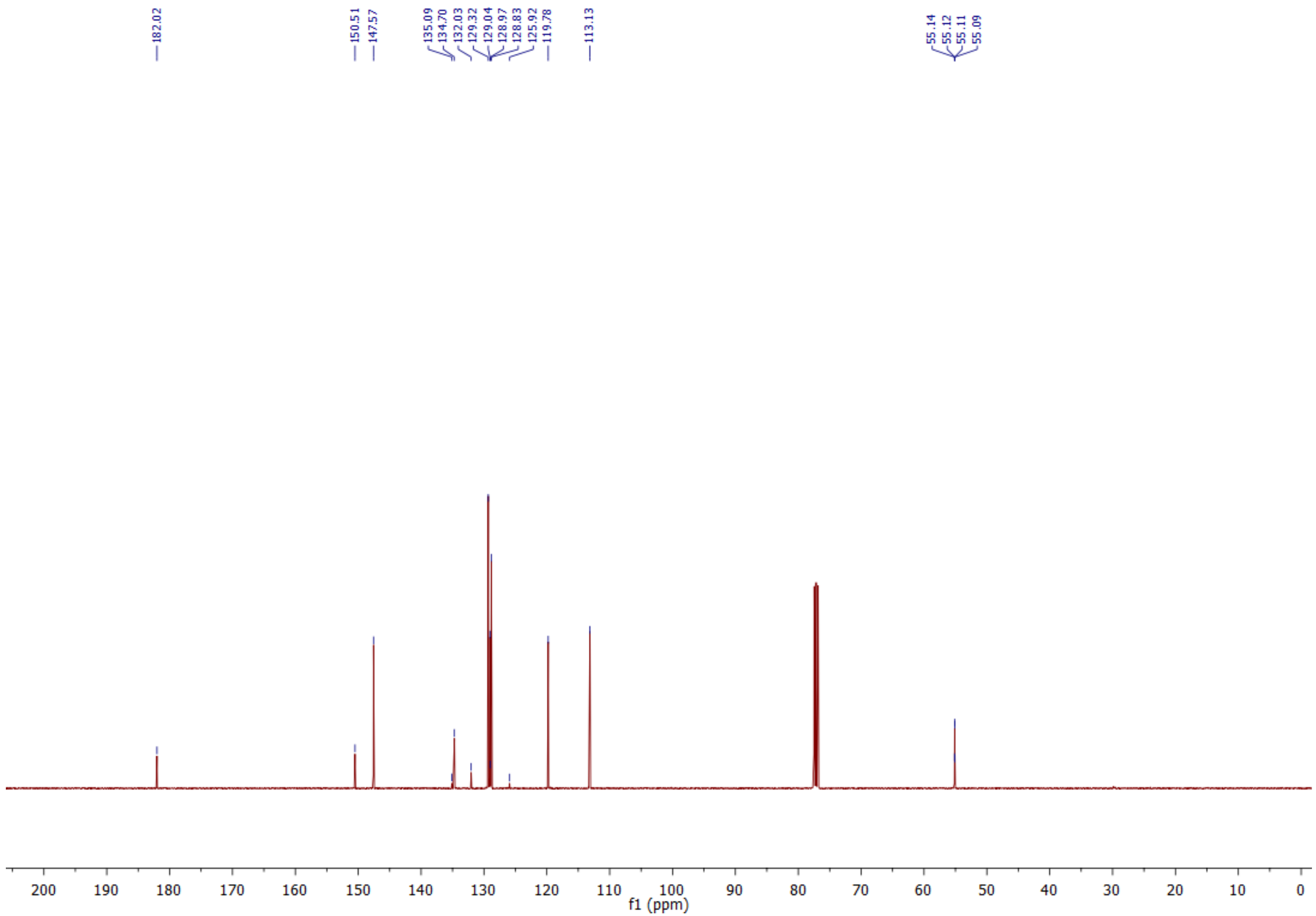

^{19}F NMR ($CDCl_3$, 377 MHz). 1-(Furan-2-yl)-2-phenyl-2-((trifluoromethyl)thio)ethan-1-one (**5g**)

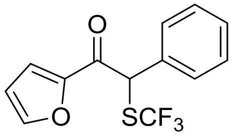

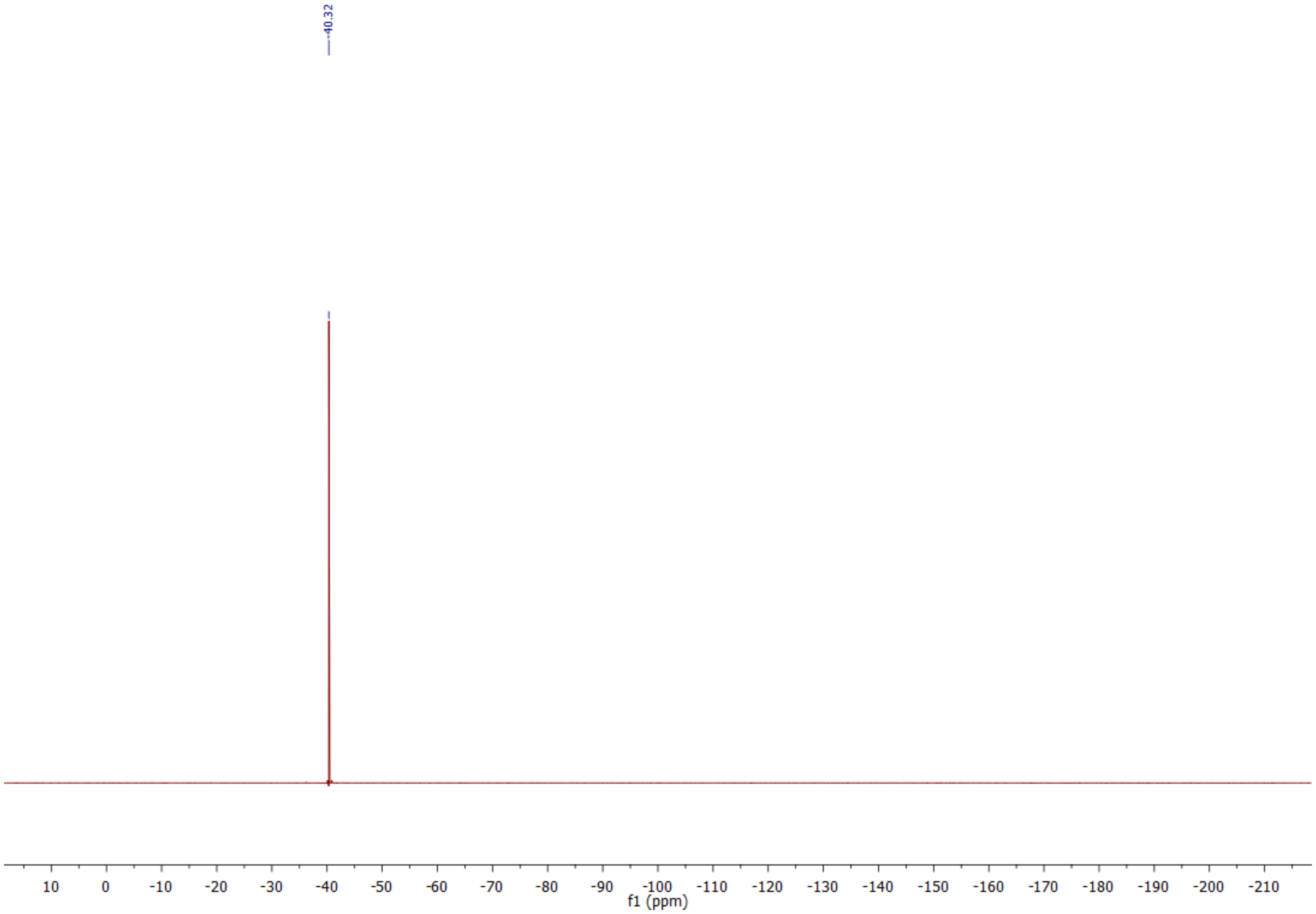

^{1}H NMR ($CDCl_3$, 400 MHz). 2-Phenyl-2-((trifluoromethyl)thio)-2,3-dihydro-1*H*-inden-1-one (**5h**)

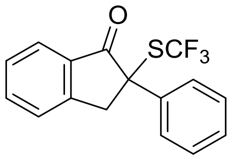

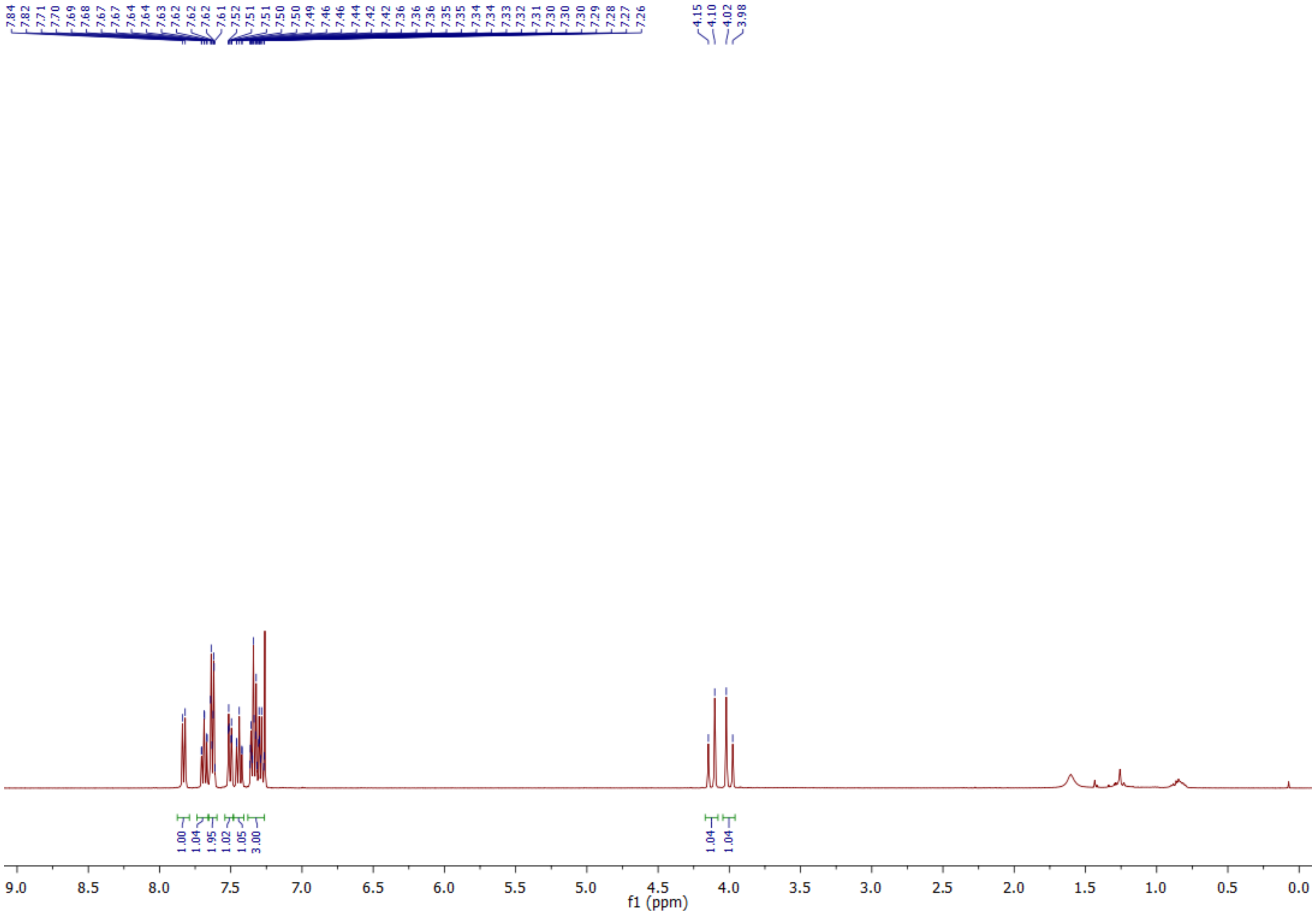

^{13}C NMR ($CDCl_3$, 100 MHz). 2-Phenyl-2-((trifluoromethyl)thio)-2,3-dihydro-1*H*-inden-1-one (**5h**)

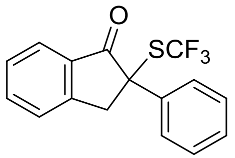

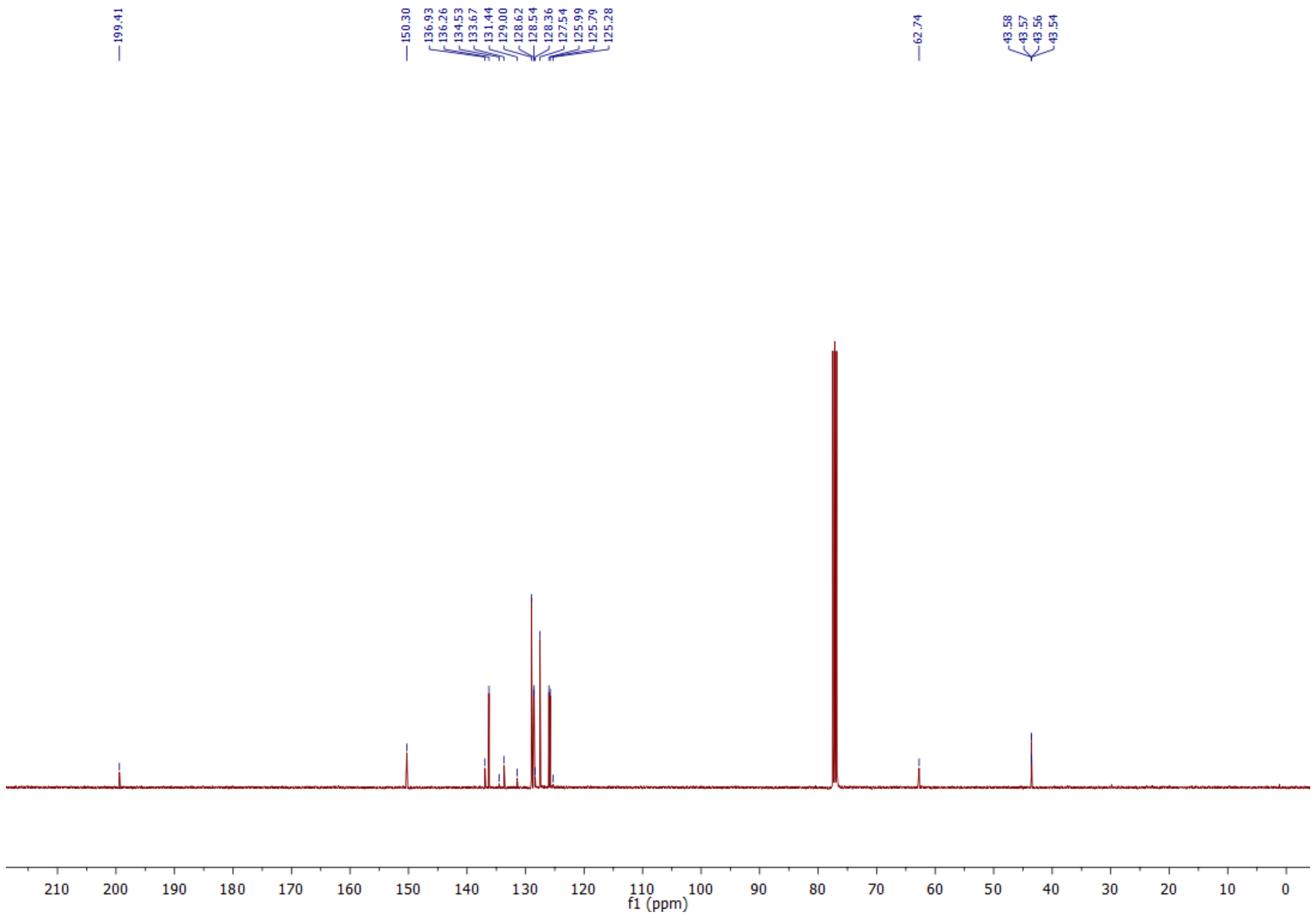

^{19}F NMR (CDCl$_3$, 377 MHz). 2-Phenyl-2-((trifluoromethyl)thio)-2,3-dihydro-1*H*-inden-1-one (**5h**)

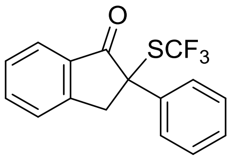

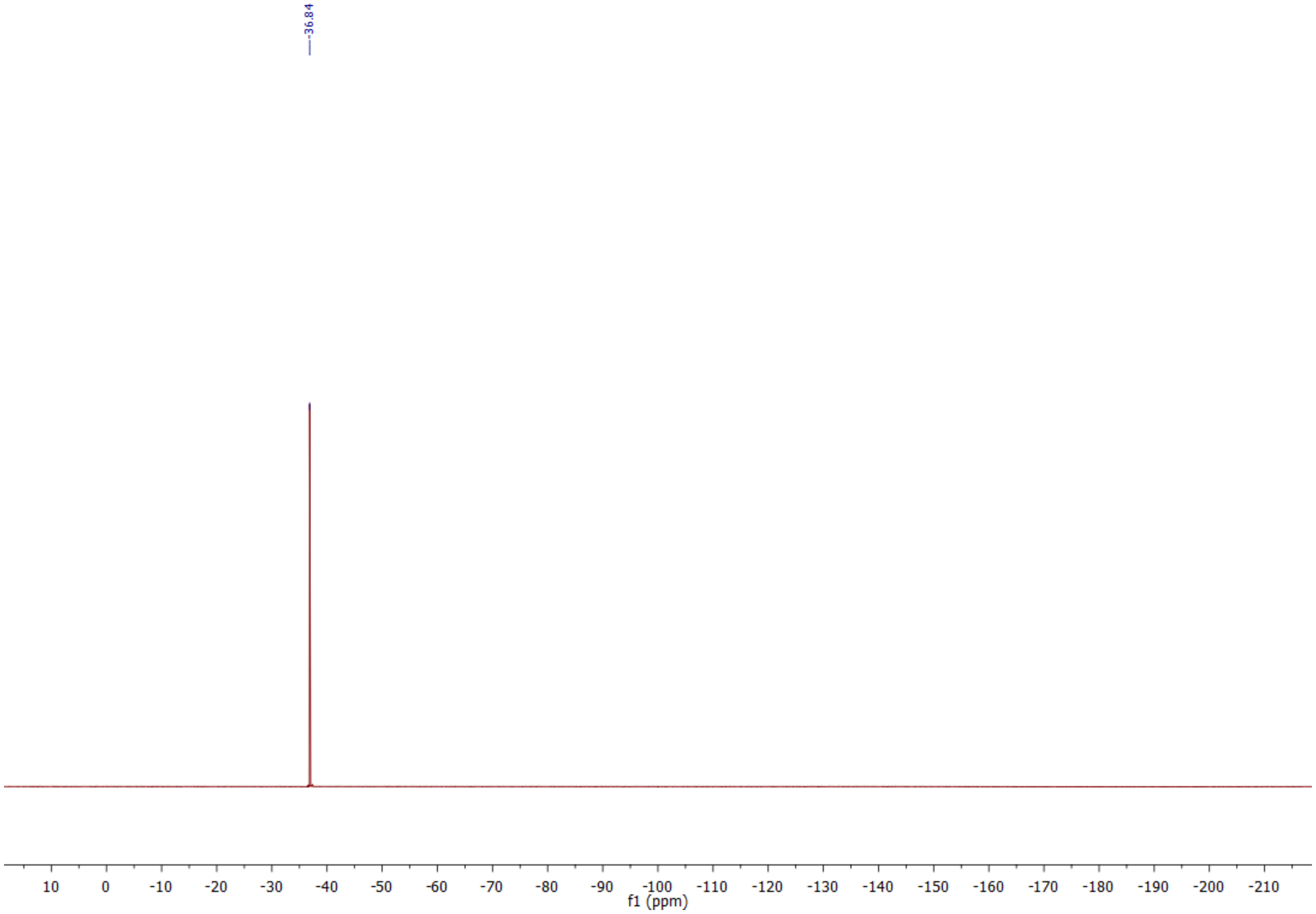

^{1}H NMR ($CDCl_3$, 400 MHz). 1-Phenyl-1-((trifluoromethyl)thio)undecan-2-one (**5i**)

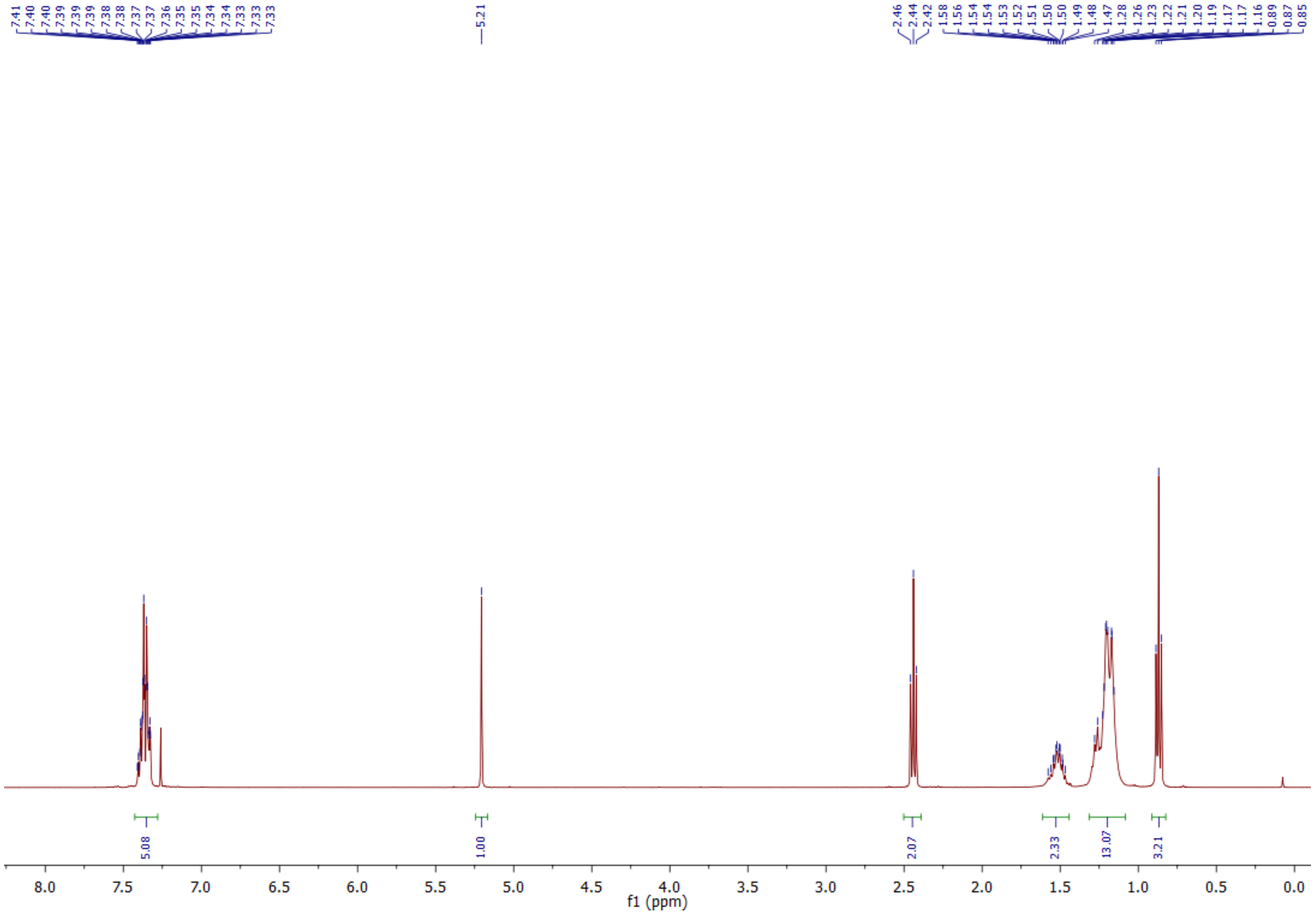

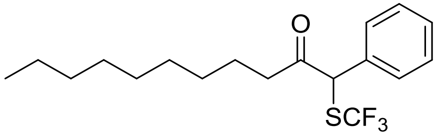

^{13}C NMR ($CDCl_3$, 100 MHz). 1-Phenyl-1-((trifluoromethyl)thio)undecan-2-one (**5i**)

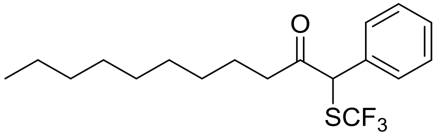

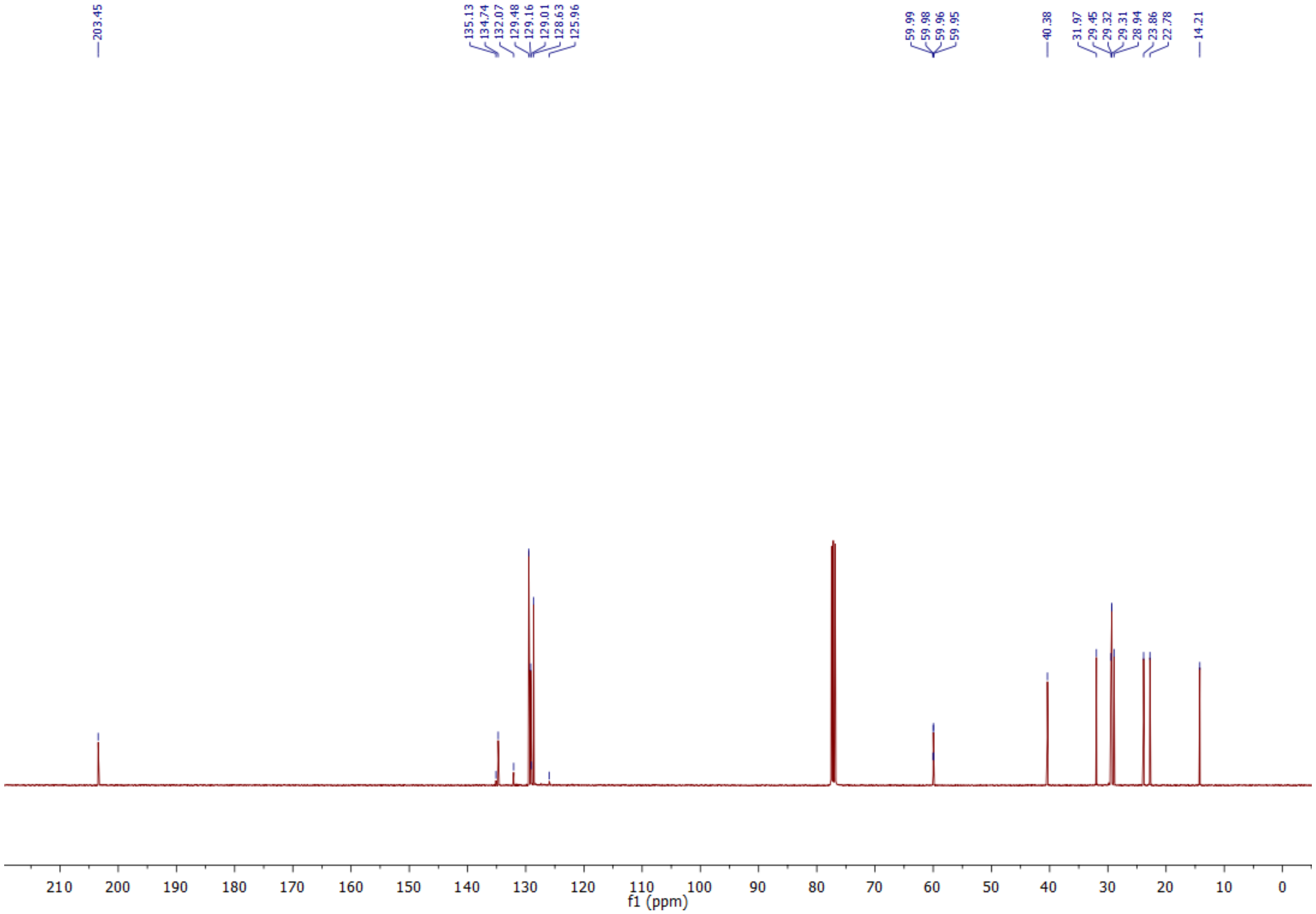

^{19}F NMR ($CDCl_3$, 377 MHz). 1-Phenyl-1-((trifluoromethyl)thio)undecan-2-one (**5i**)

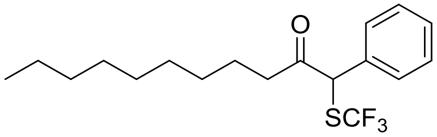

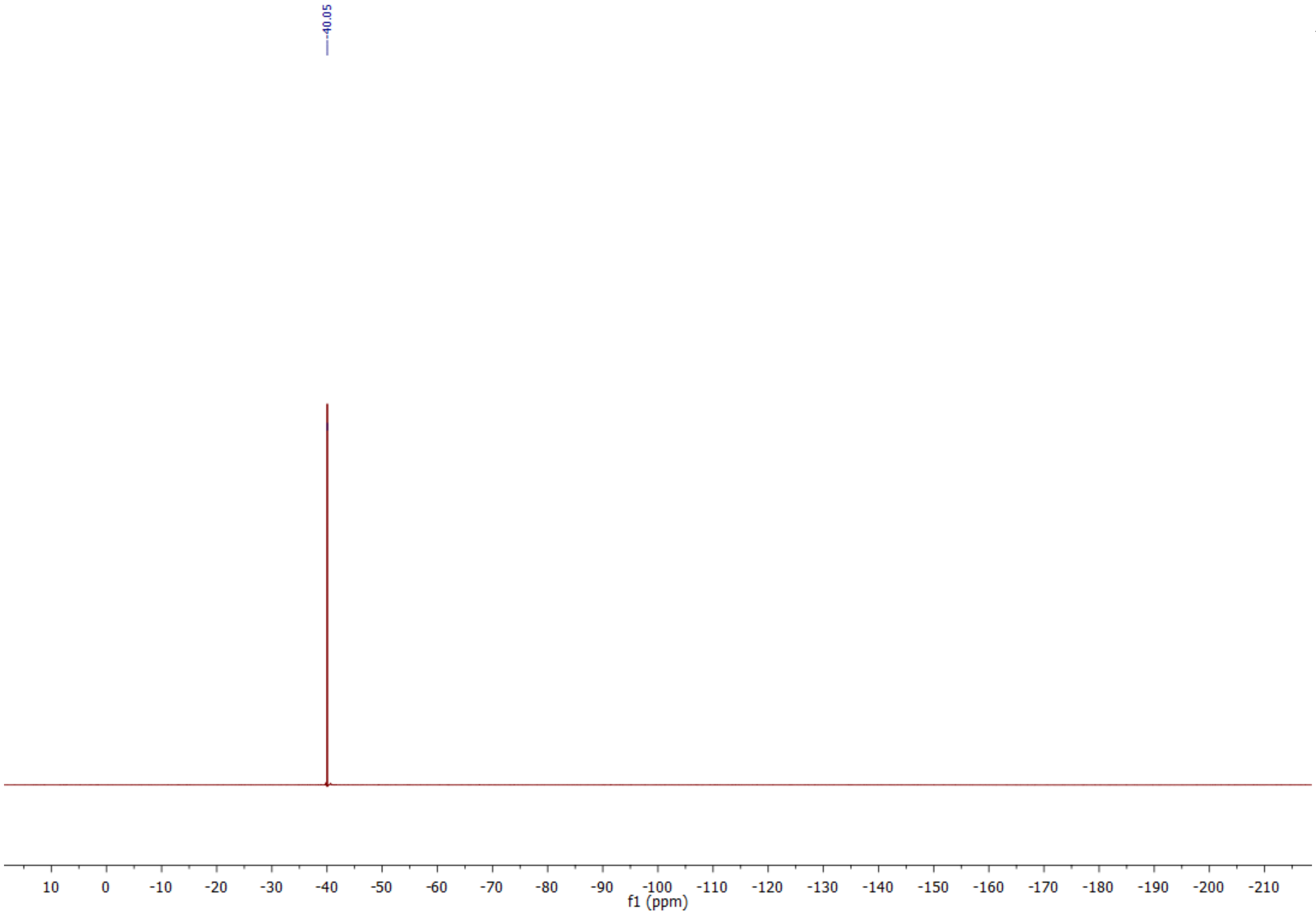

^{1}H NMR ($CDCl_3$, 400 MHz). 1-Cyclopentyl-2-phenyl-2-((trifluoromethyl)thio)ethan-1-one (**5j**)

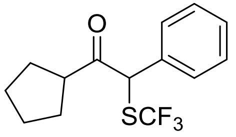

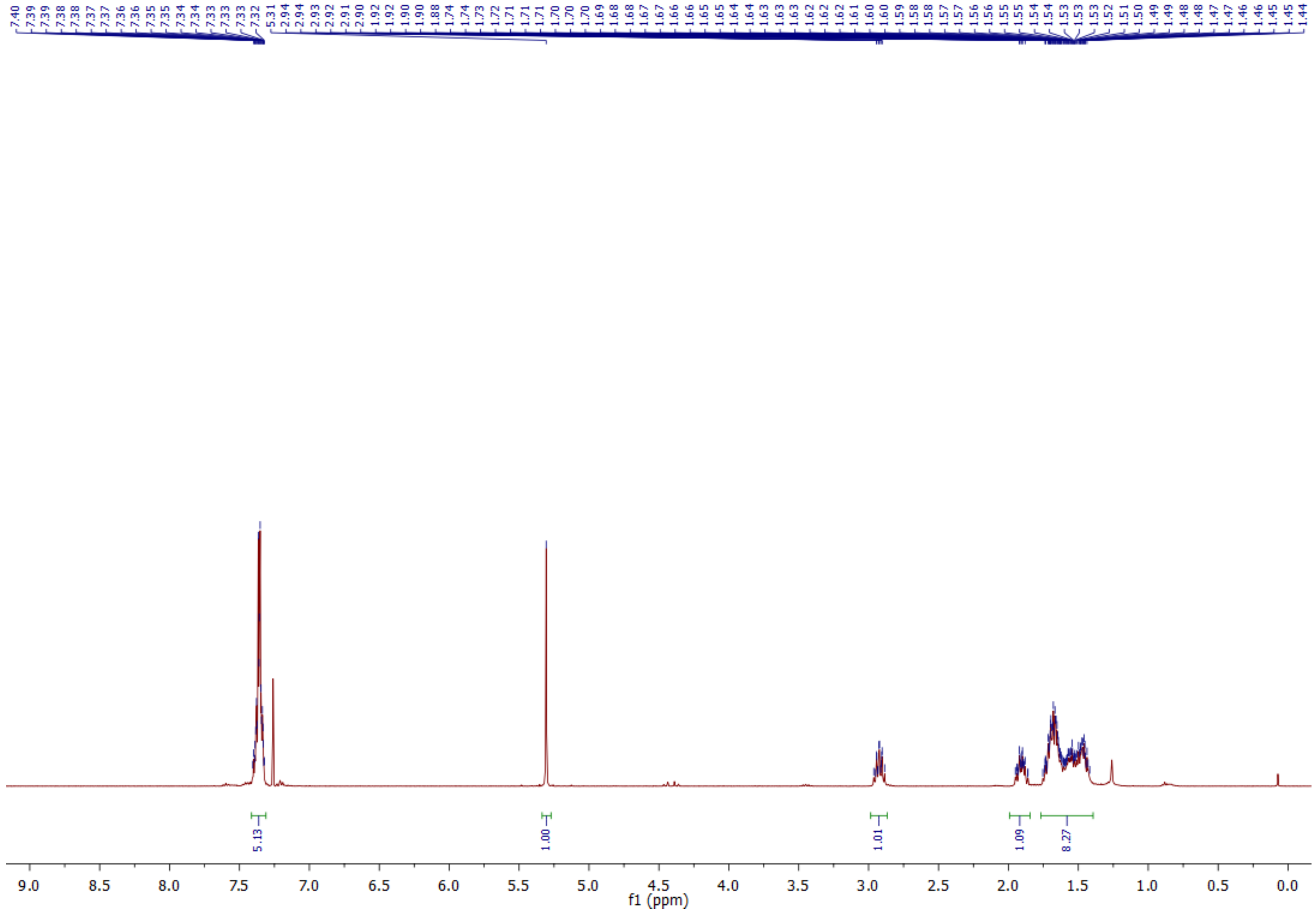

^{13}C NMR ($CDCl_3$, 100 MHz). 1-Cyclopentyl-2-phenyl-2-((trifluoromethyl)thio)ethan-1-one (**5j**)

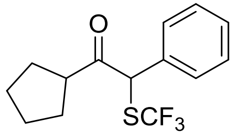

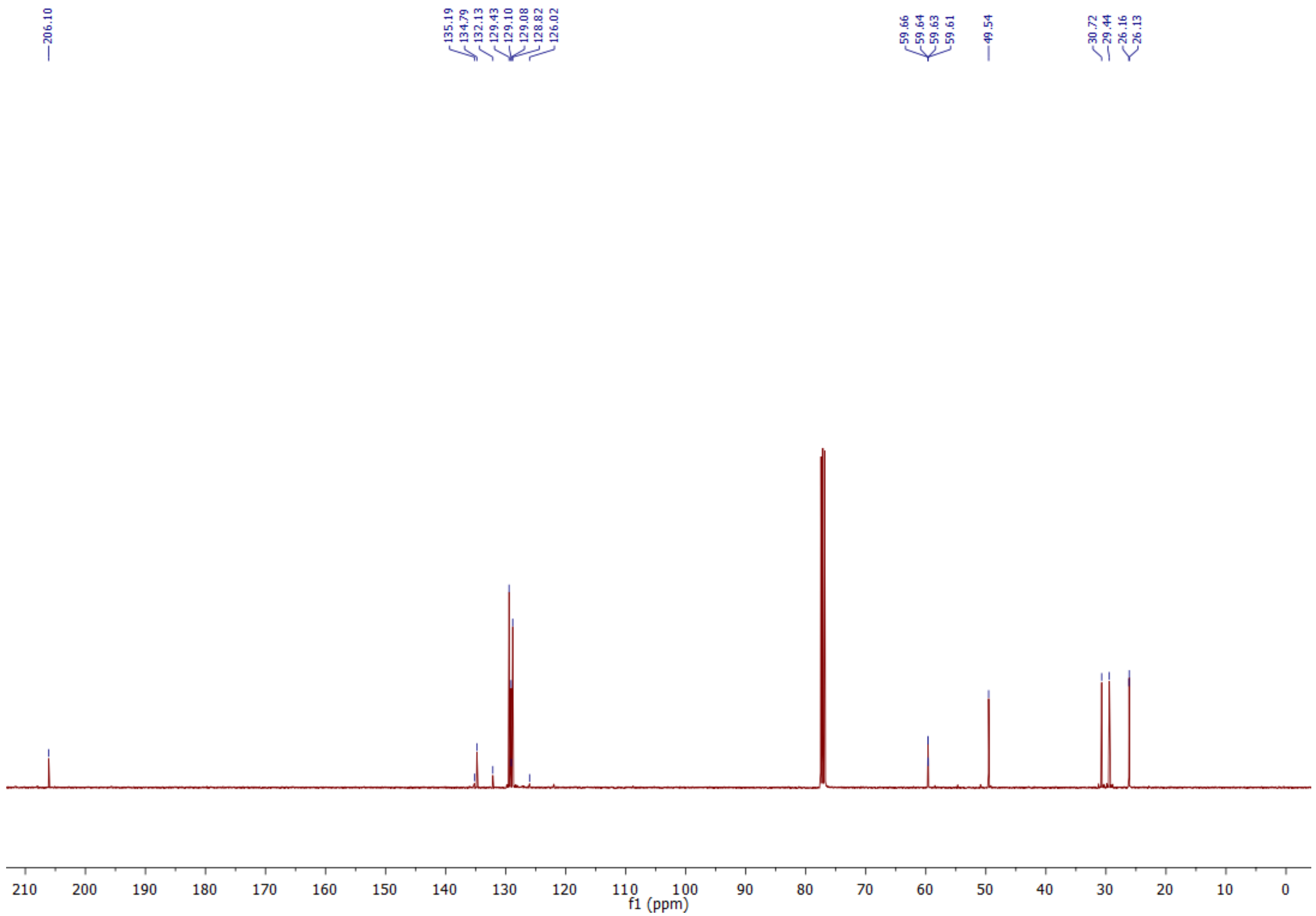

^{19}F NMR ($CDCl_3$, 377 MHz). 1-Cyclopentyl-2-phenyl-2-((trifluoromethyl)thio)ethan-1-one (**5j**)

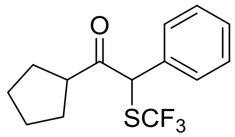

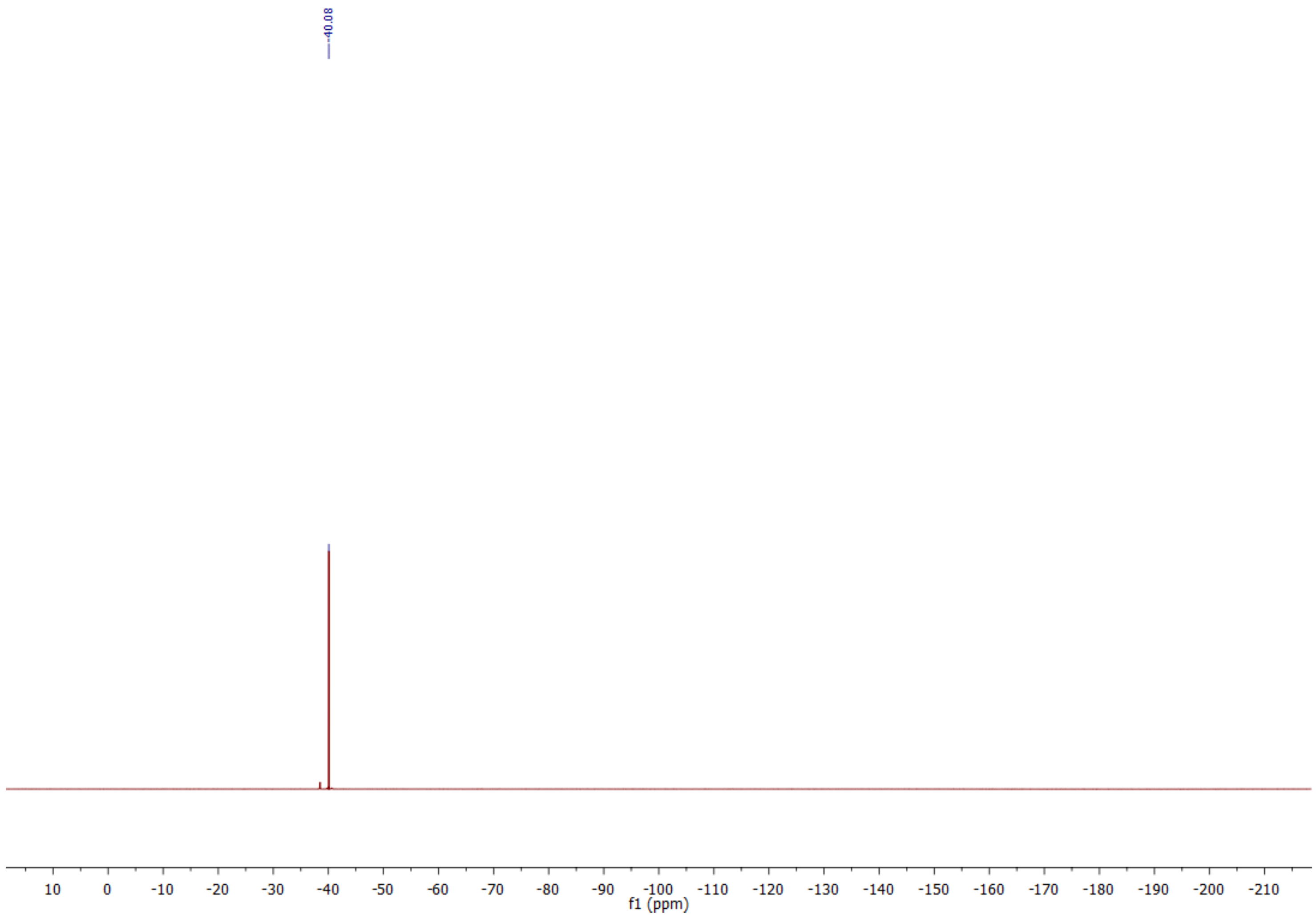

^1H NMR ($CDCl_3$, 400 MHz). Ethyl 2-phenyl-2-((trifluoromethyl)thio)acetate (**5k**)

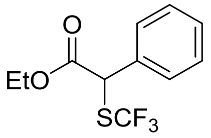

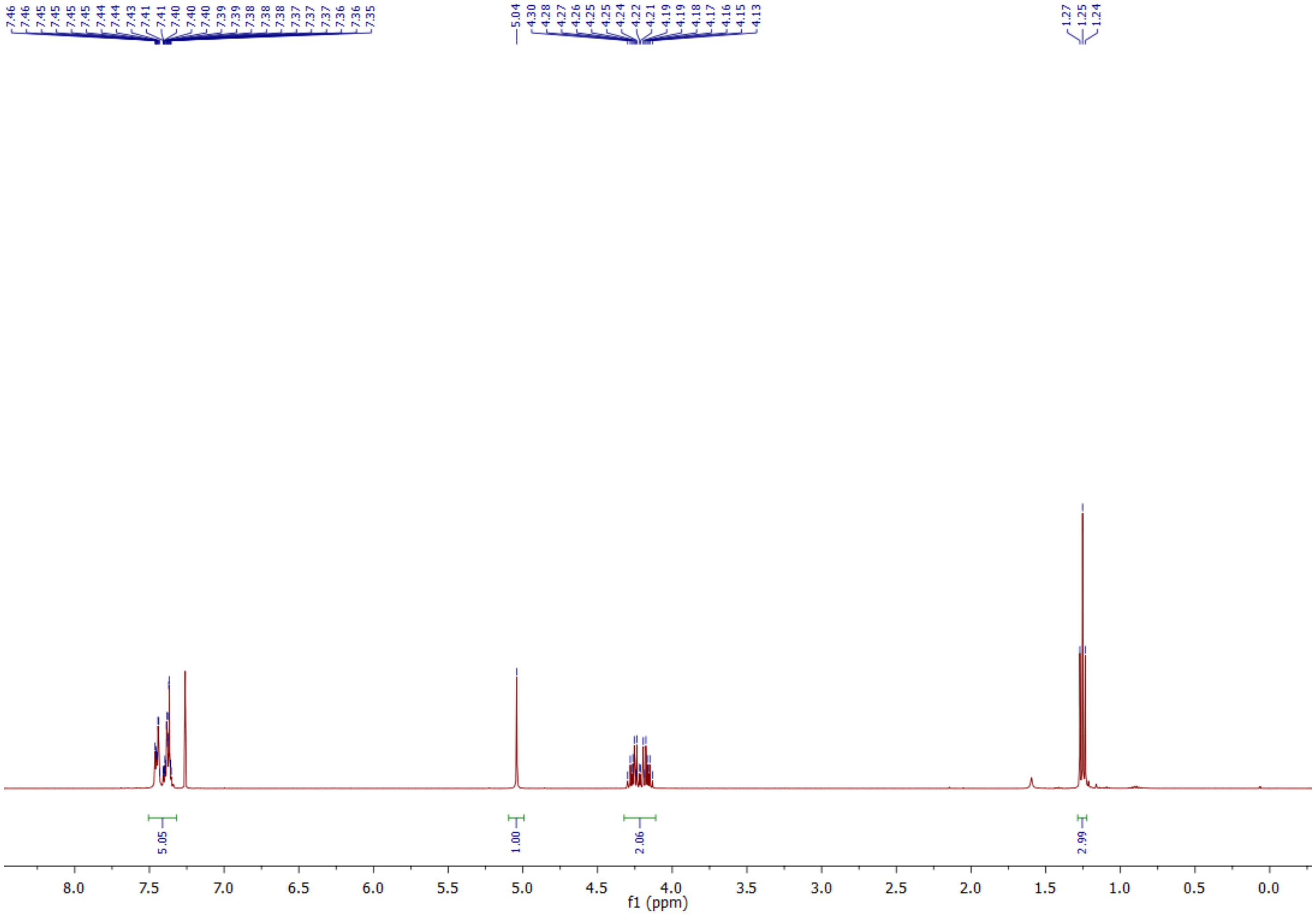

^{13}C NMR ($CDCl_3$, 100 MHz). Ethyl 2-phenyl-2-((trifluoromethyl)thio)acetate (**5k**)

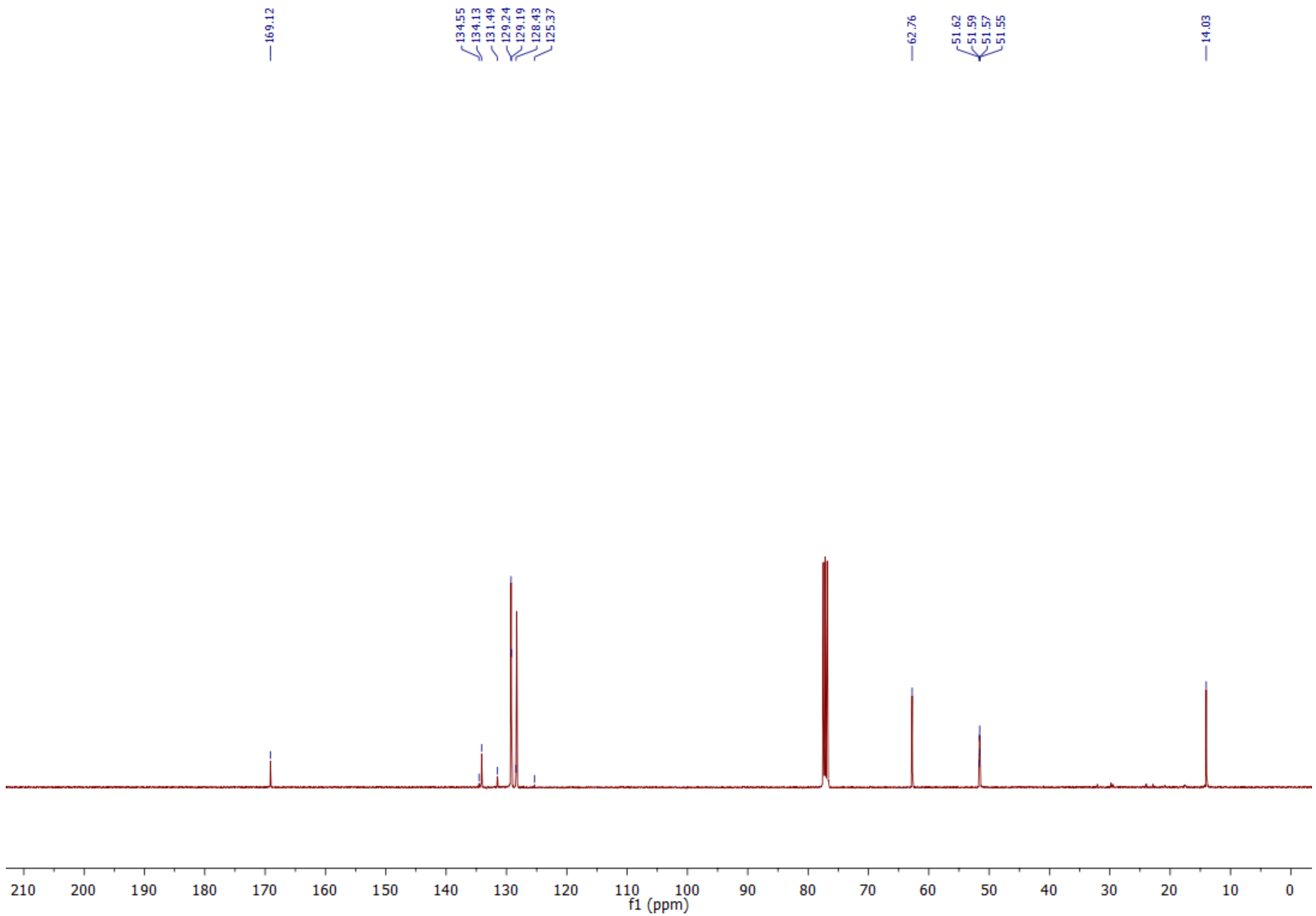

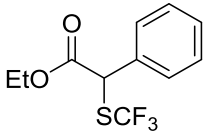

^{19}F NMR ($CDCl_3$, 377 MHz). Ethyl 2-phenyl-2-((trifluoromethyl)thio)acetate (**5k**)

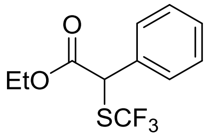

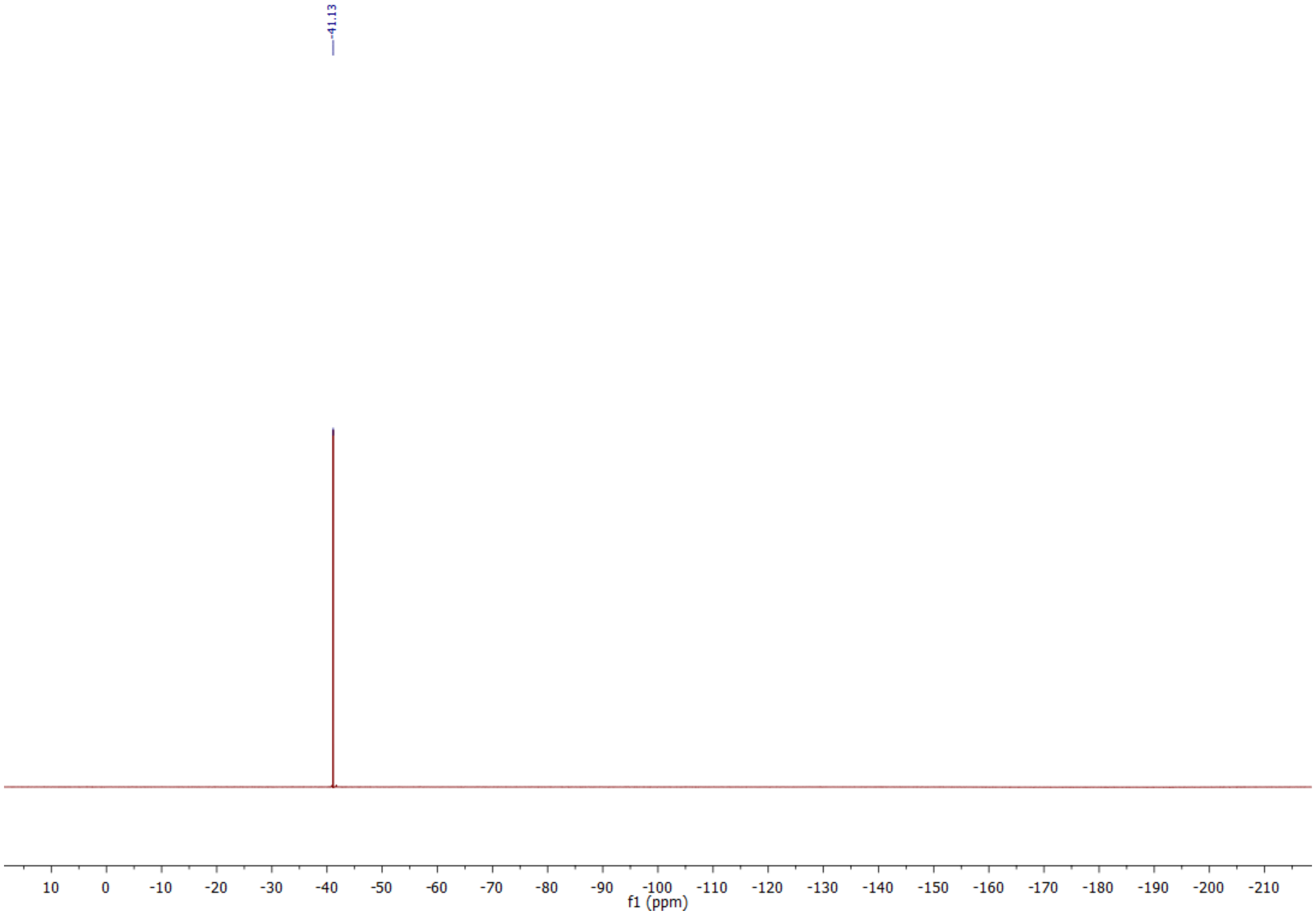

^{1}H NMR ($CDCl_3$, 400 MHz). 1-Morpholino-2-phenyl-2-((trifluoromethyl)thio)ethan-1-one (**5m**)

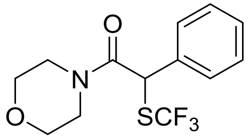

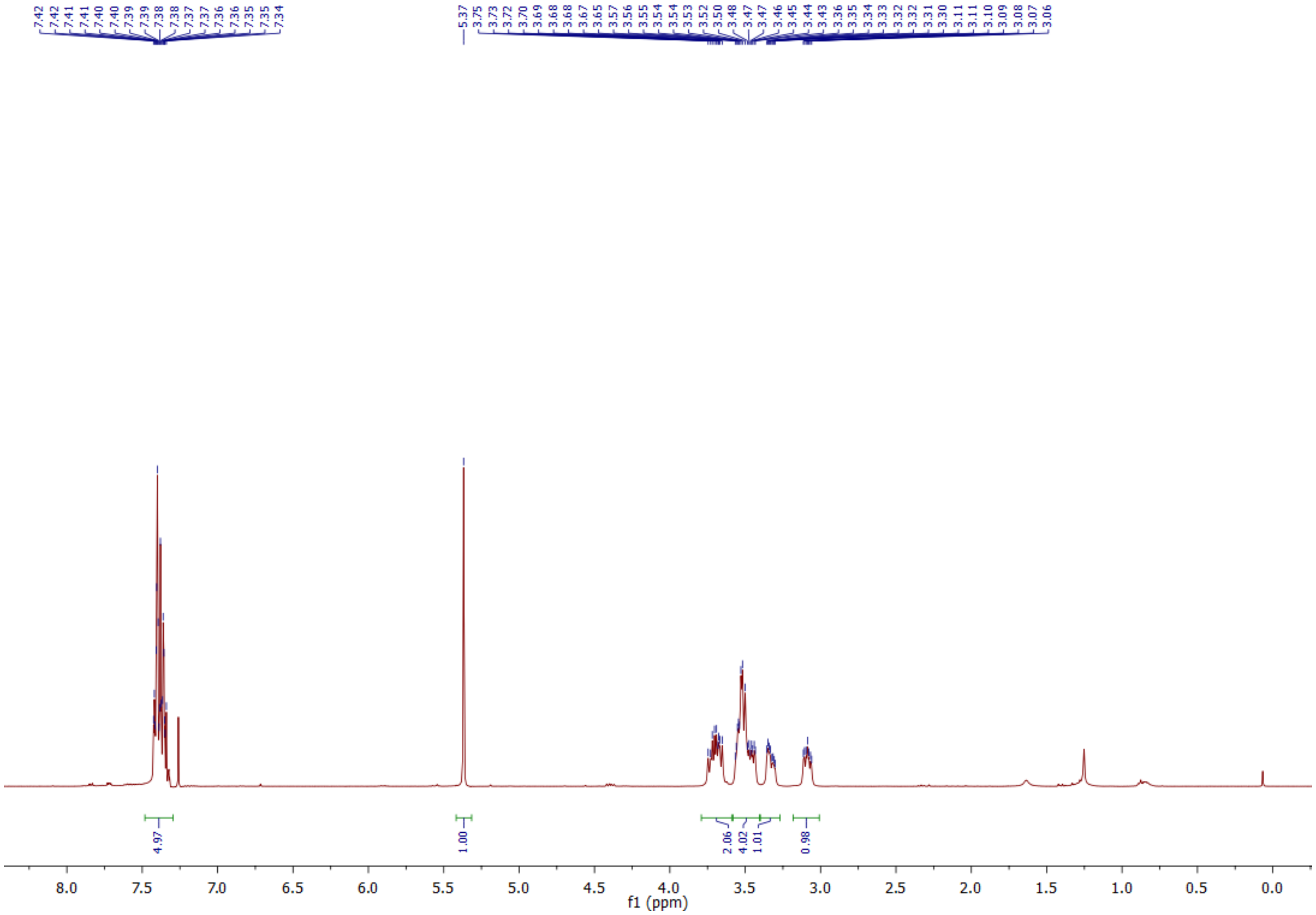

^{13}C NMR ($CDCl_3$, 100 MHz). 1-Morpholino-2-phenyl-2-((trifluoromethyl)thio)ethan-1-one (**5m**)

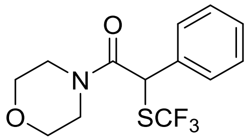

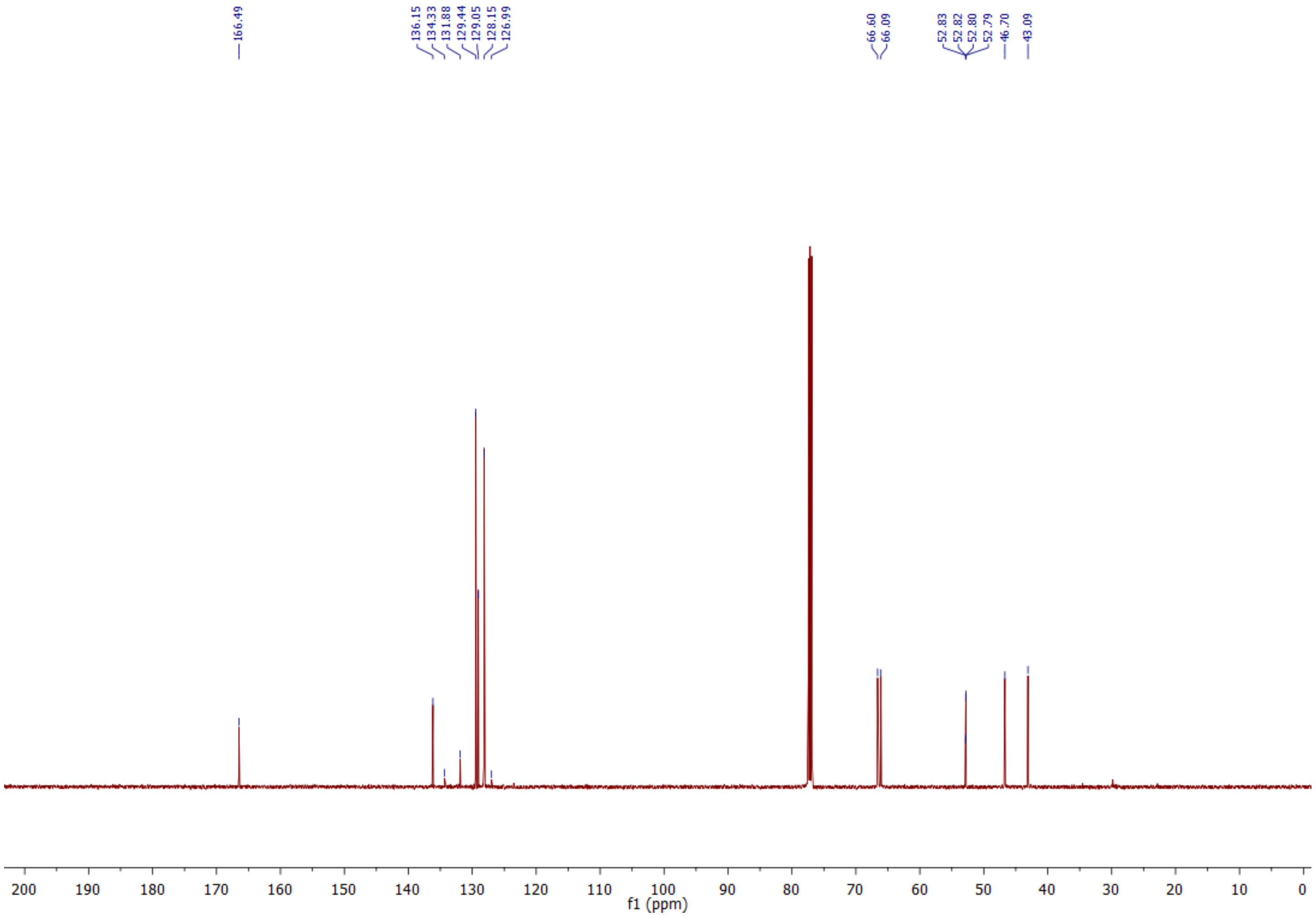

^{19}F NMR ($CDCl_3$, 377 MHz). 1-Morpholino-2-phenyl-2-((trifluoromethyl)thio)ethan-1-one (**5m**)

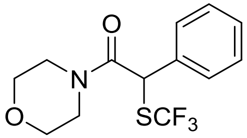

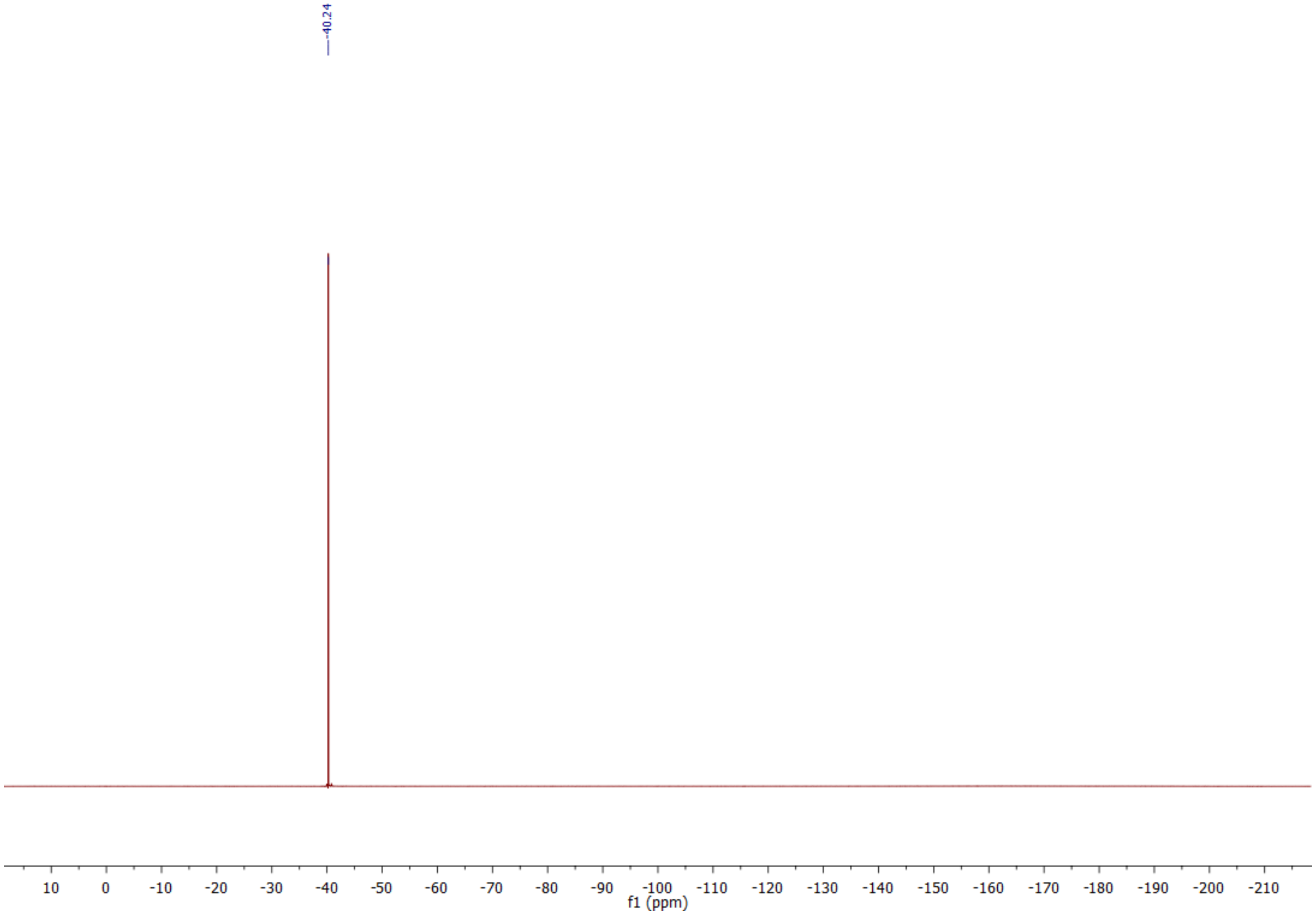

^{1}H NMR ($CDCl_3$, 400 MHz). Ethyl 2-(4-chlorophenyl)-2-((trifluoromethyl)thio)acetate (**5o**)

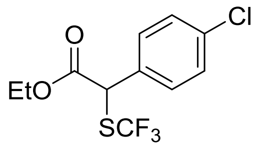

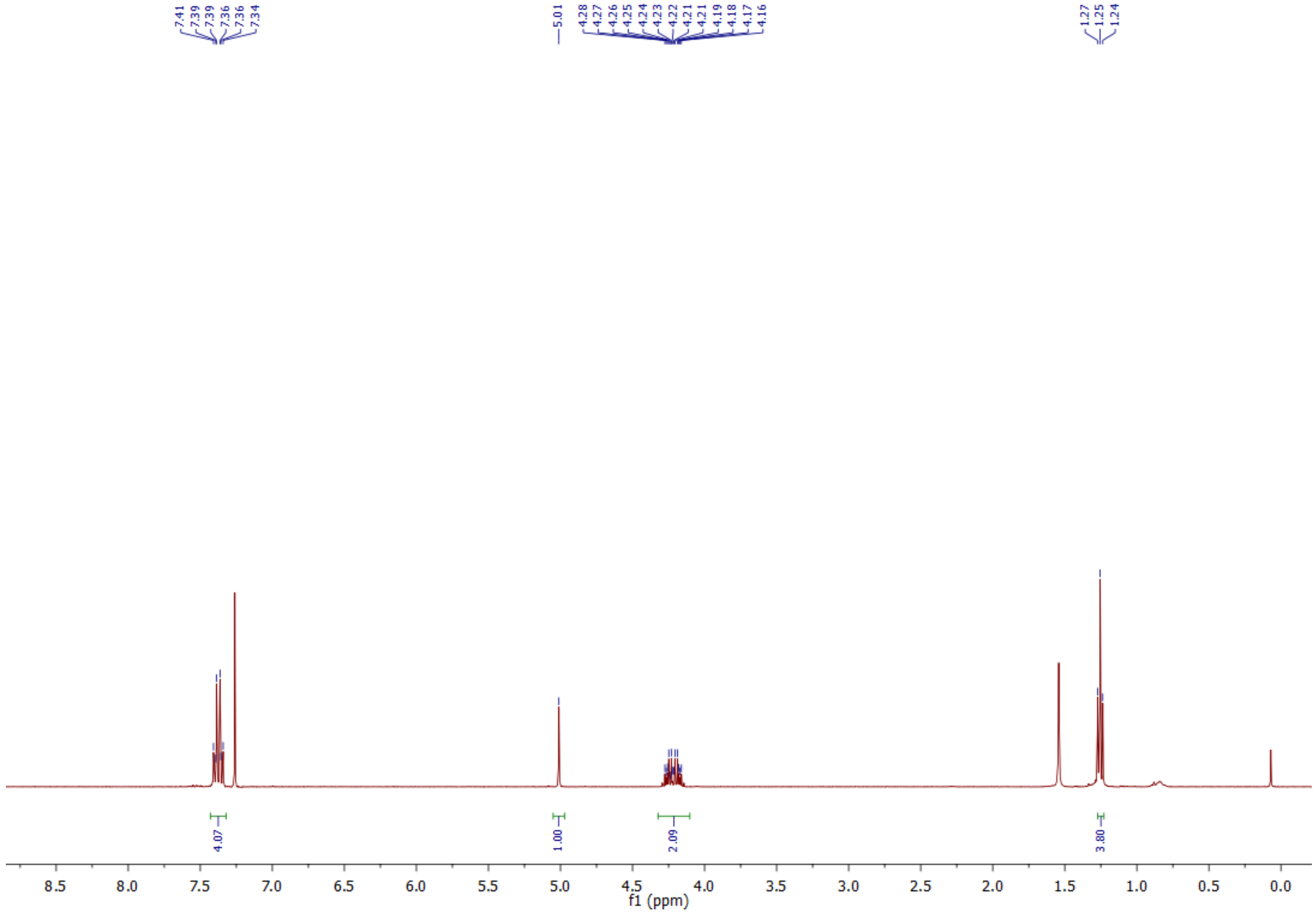

^{13}C NMR ($CDCl_3$, 100 MHz). Ethyl 2-(4-chlorophenyl)-2-((trifluoromethyl)thio)acetate (**5o**)

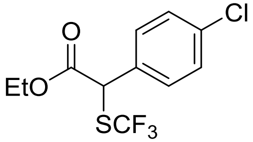

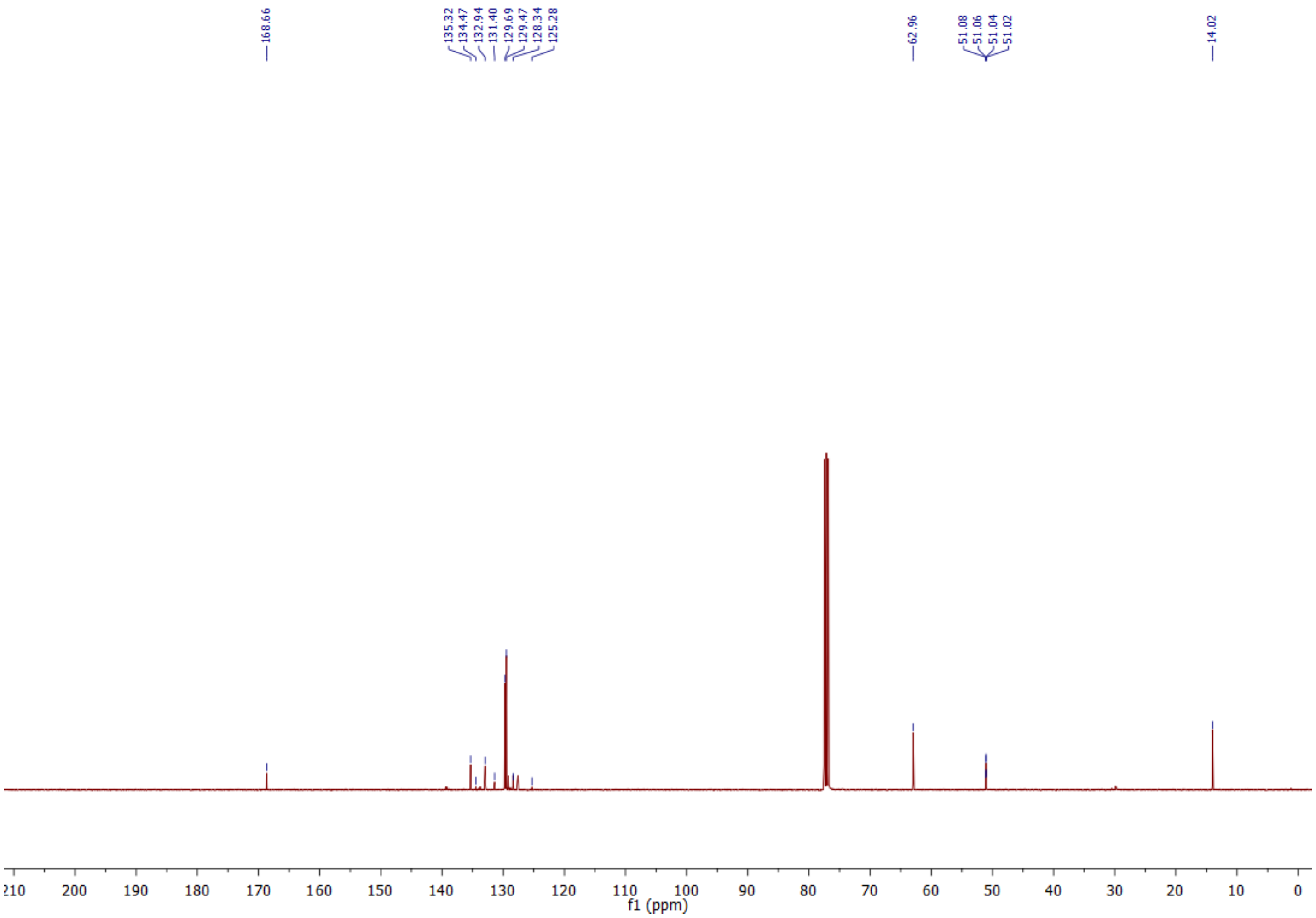

^{19}F NMR ($CDCl_3$, 377 MHz). Ethyl 2-(4-chlorophenyl)-2-((trifluoromethyl)thio)acetate (**5o**)

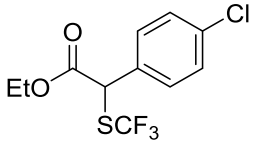

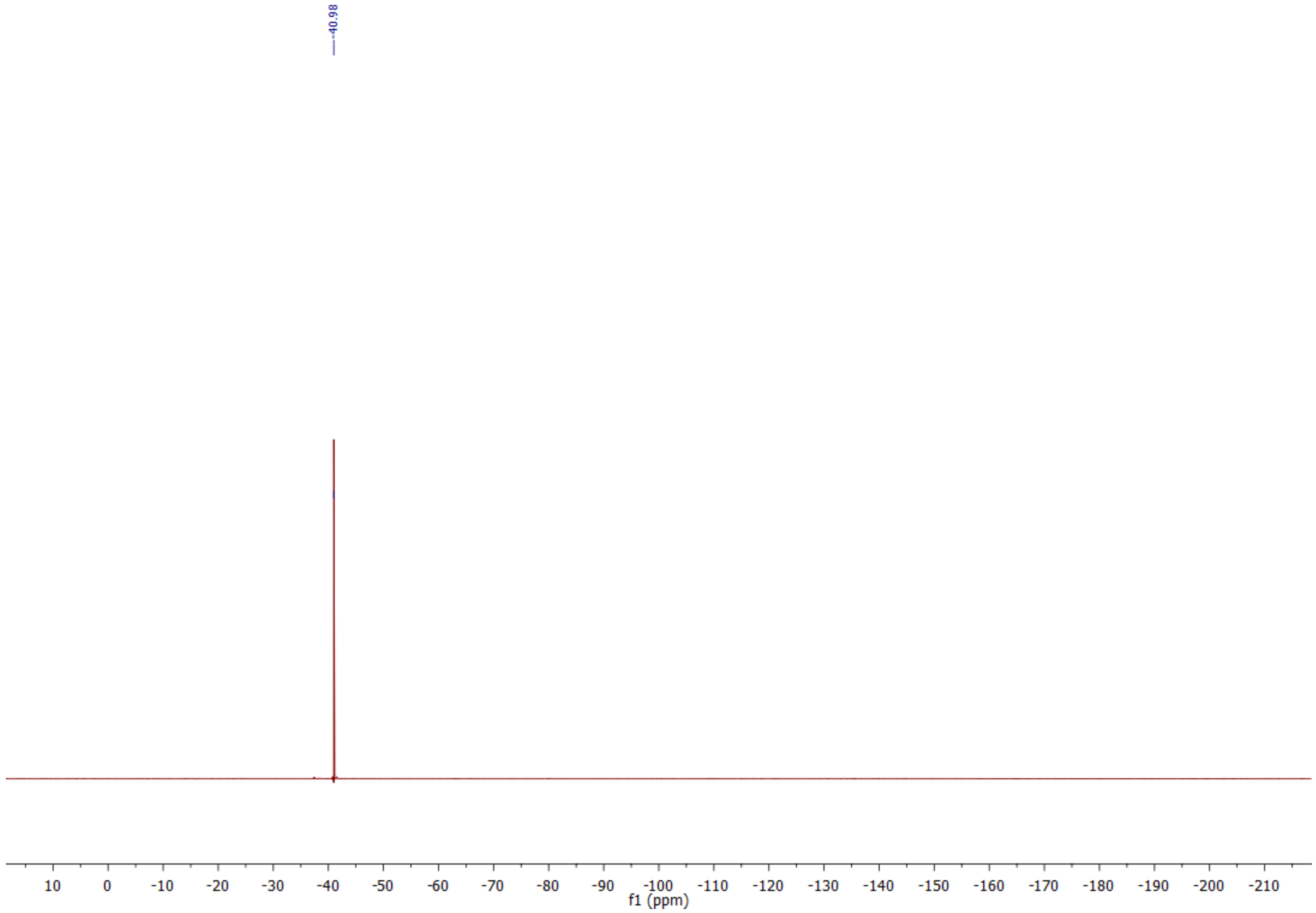

^1H NMR ($CDCl_3$, 400 MHz). 1-(4-Bromophenyl)-2-(4-chlorophenyl)-2-((trifluoromethyl)thio)ethan-1-one (**5p**)

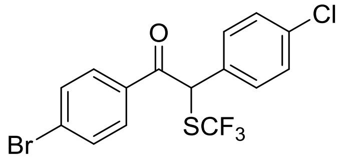

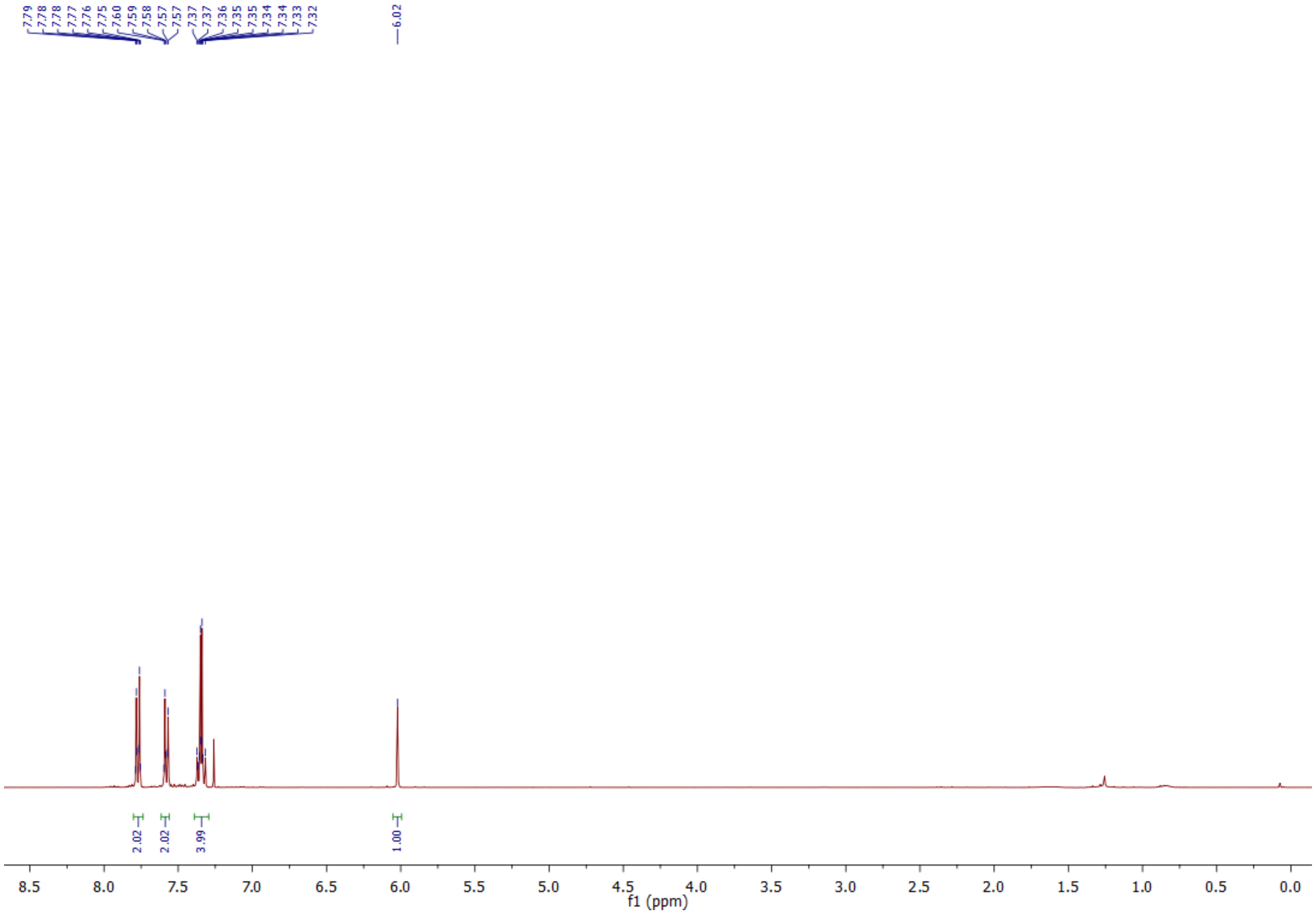

^{13}C NMR ($CDCl_3$, 100 MHz). 1-(4-Bromophenyl)-2-(4-chlorophenyl)-2-((trifluoromethyl)thio)ethan-1-one (**5p**)

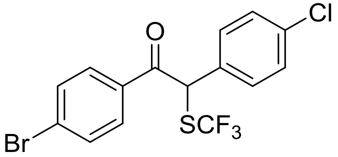

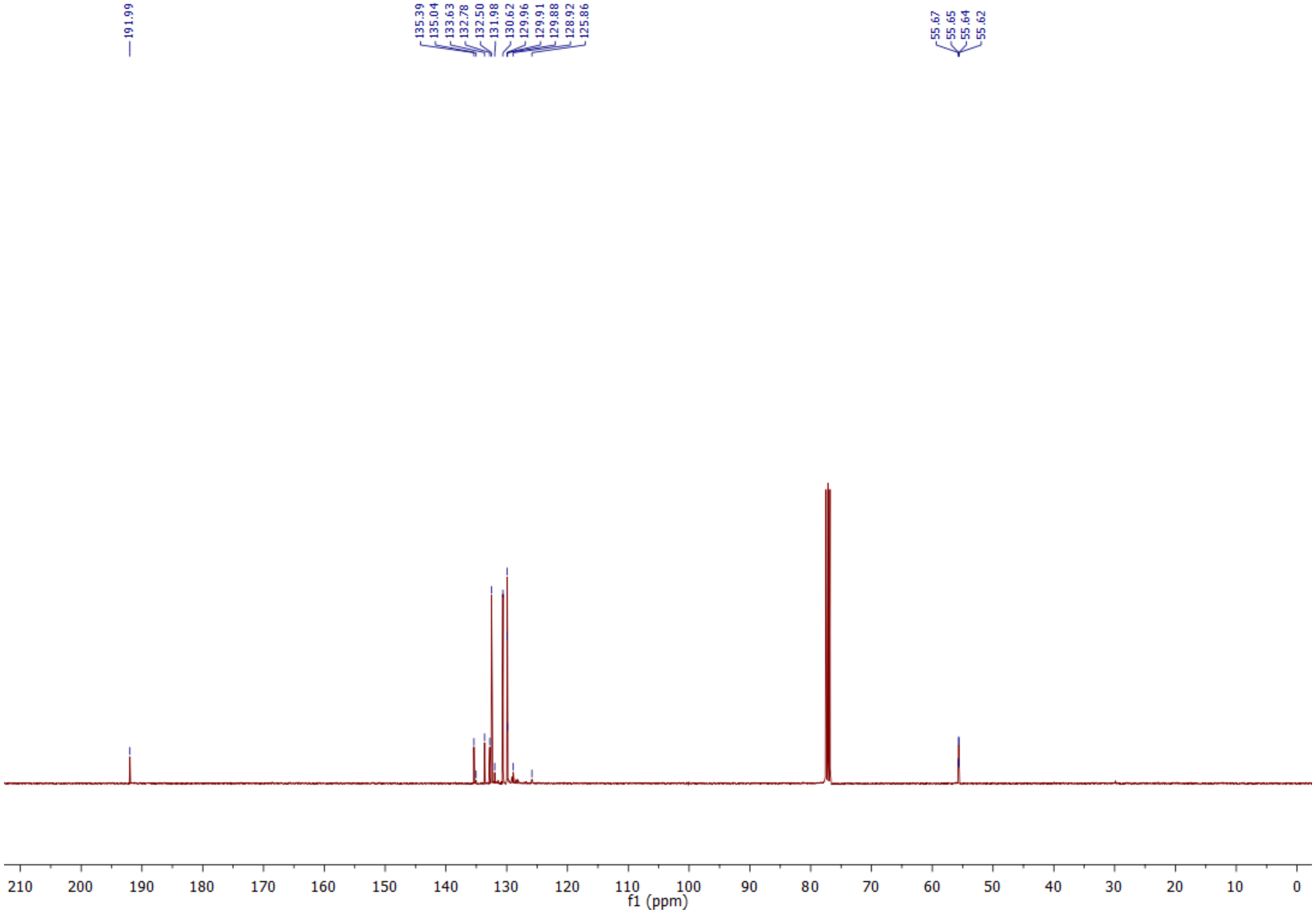

^{19}F NMR ($CDCl_3$, 377 MHz). 1-(4-Bromophenyl)-2-(4-chlorophenyl)-2-((trifluoromethyl)thio)ethan-1-one (**5p**)

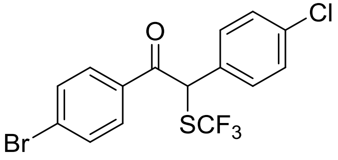

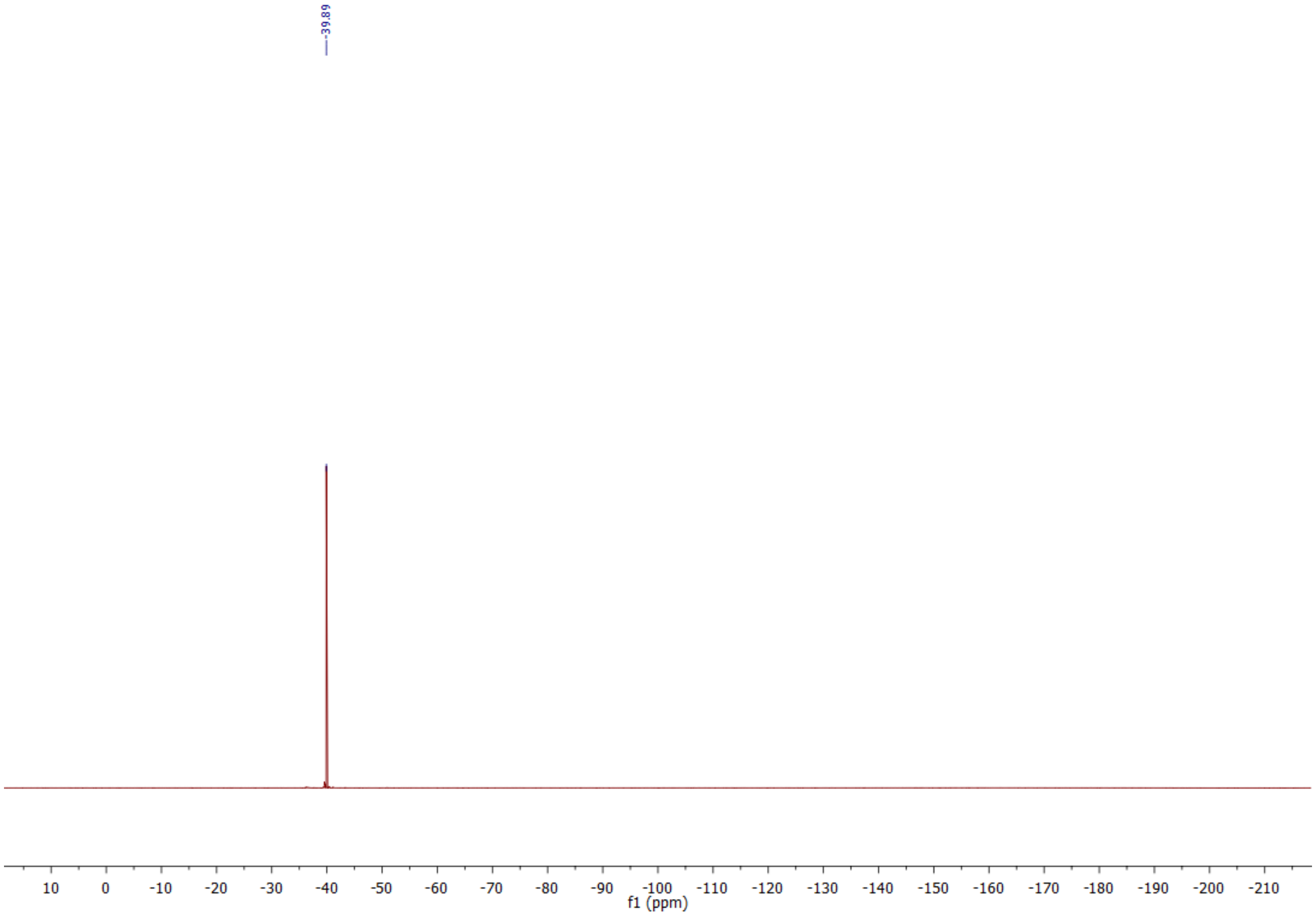

^{1}H NMR ($CDCl_3$, 400 MHz). 1-Phenyl-2-(thiophen-2-yl)-2-((trifluoromethyl)thio)ethan-1-one (**5q**)

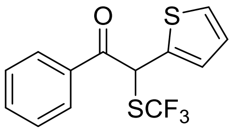

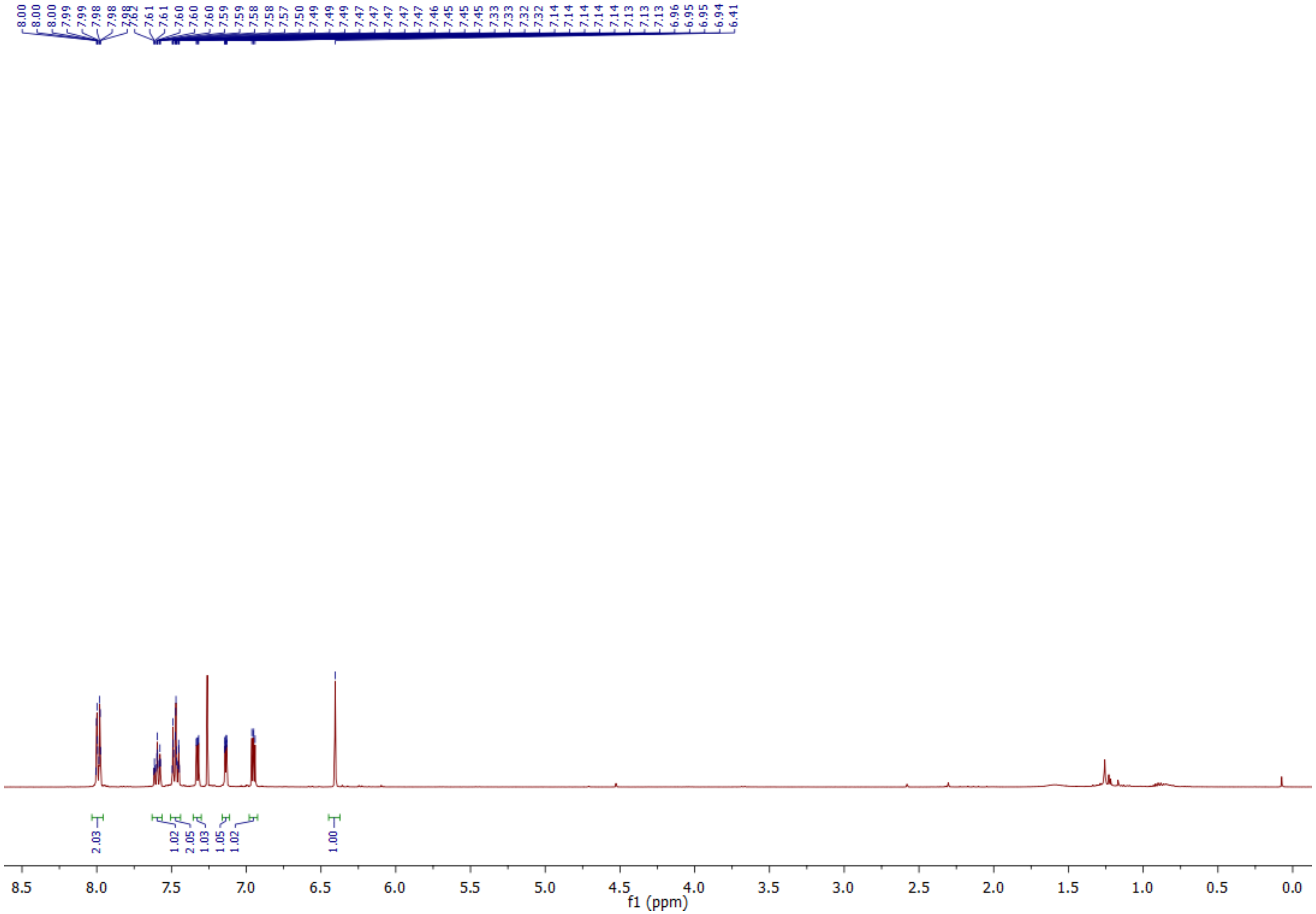

^{13}C NMR ($CDCl_3$, 100 MHz). 1-Phenyl-2-(thiophen-2-yl)-2-((trifluoromethyl)thio)ethan-1-one (**5q**)

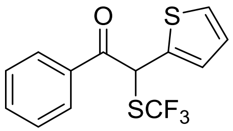

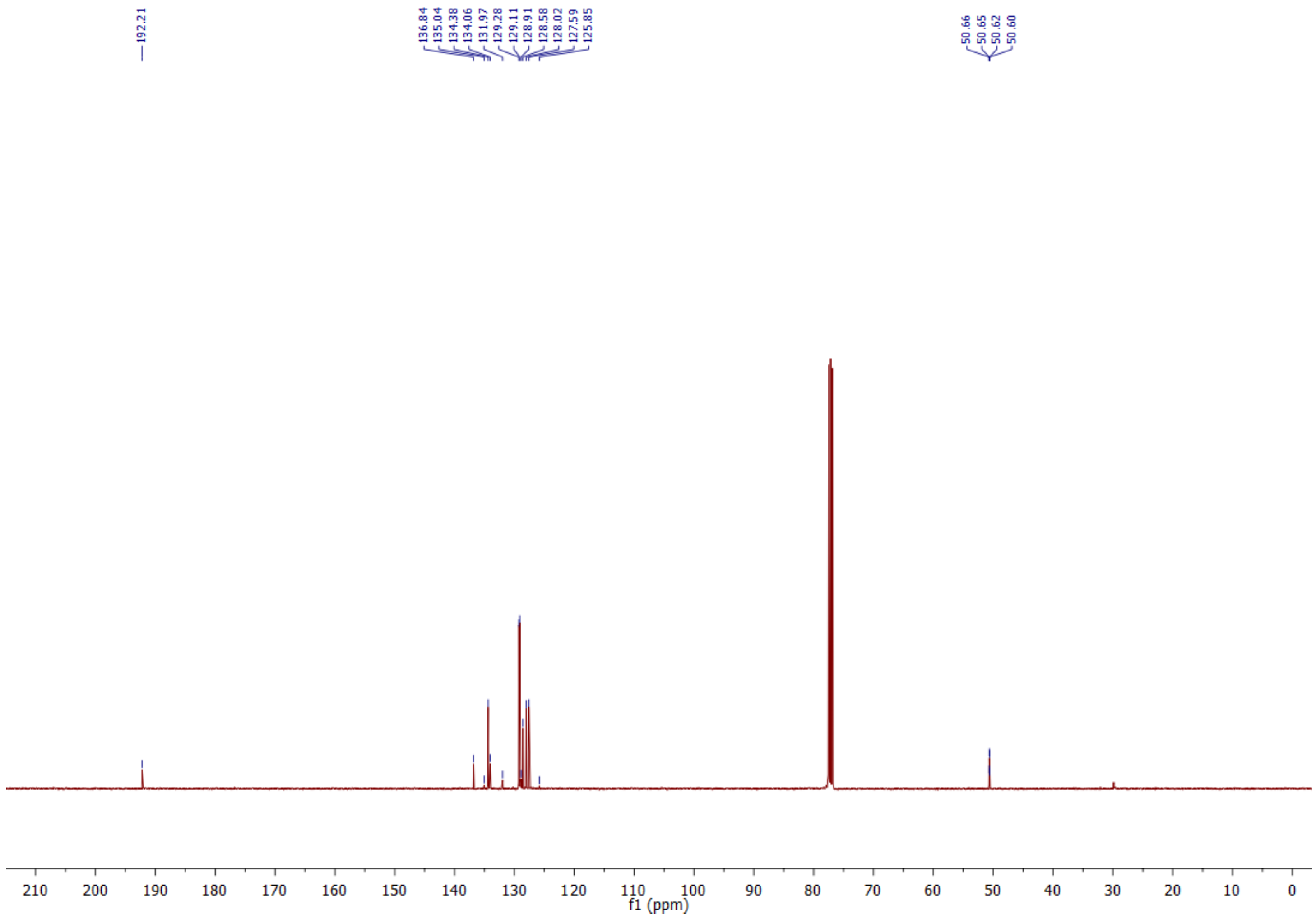

^{19}F NMR ($CDCl_3$, 377 MHz). 1-Phenyl-2-(thiophen-2-yl)-2-((trifluoromethyl)thio)ethan-1-one (**5q**)

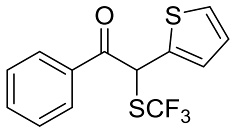

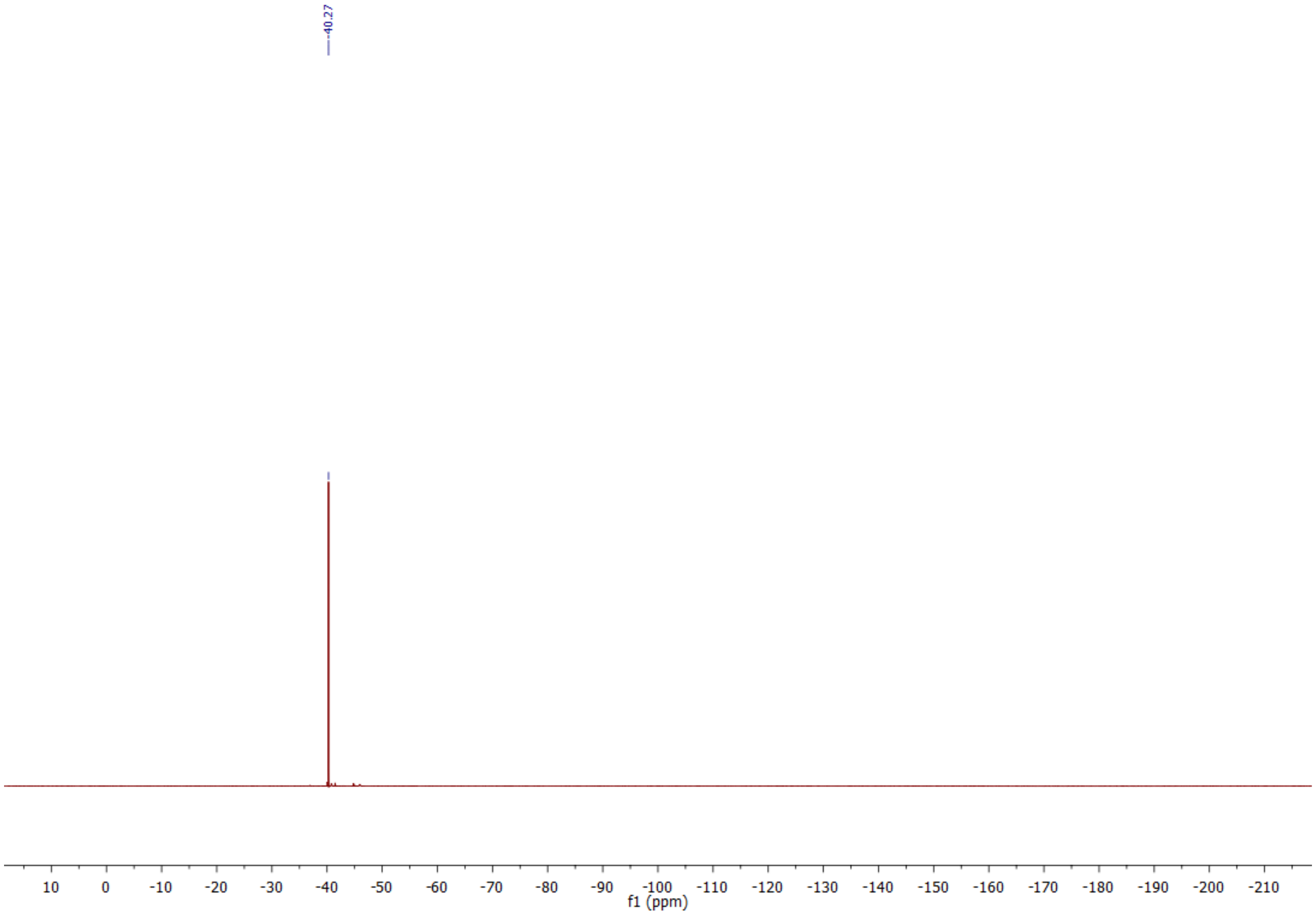

^1H NMR ($CDCl_3$, 400 MHz). 1-(Furan-2-yl)-2-(thiophen-2-yl)-2-((trifluoromethyl)thio)ethan-1-one (**5r**)

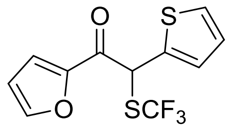

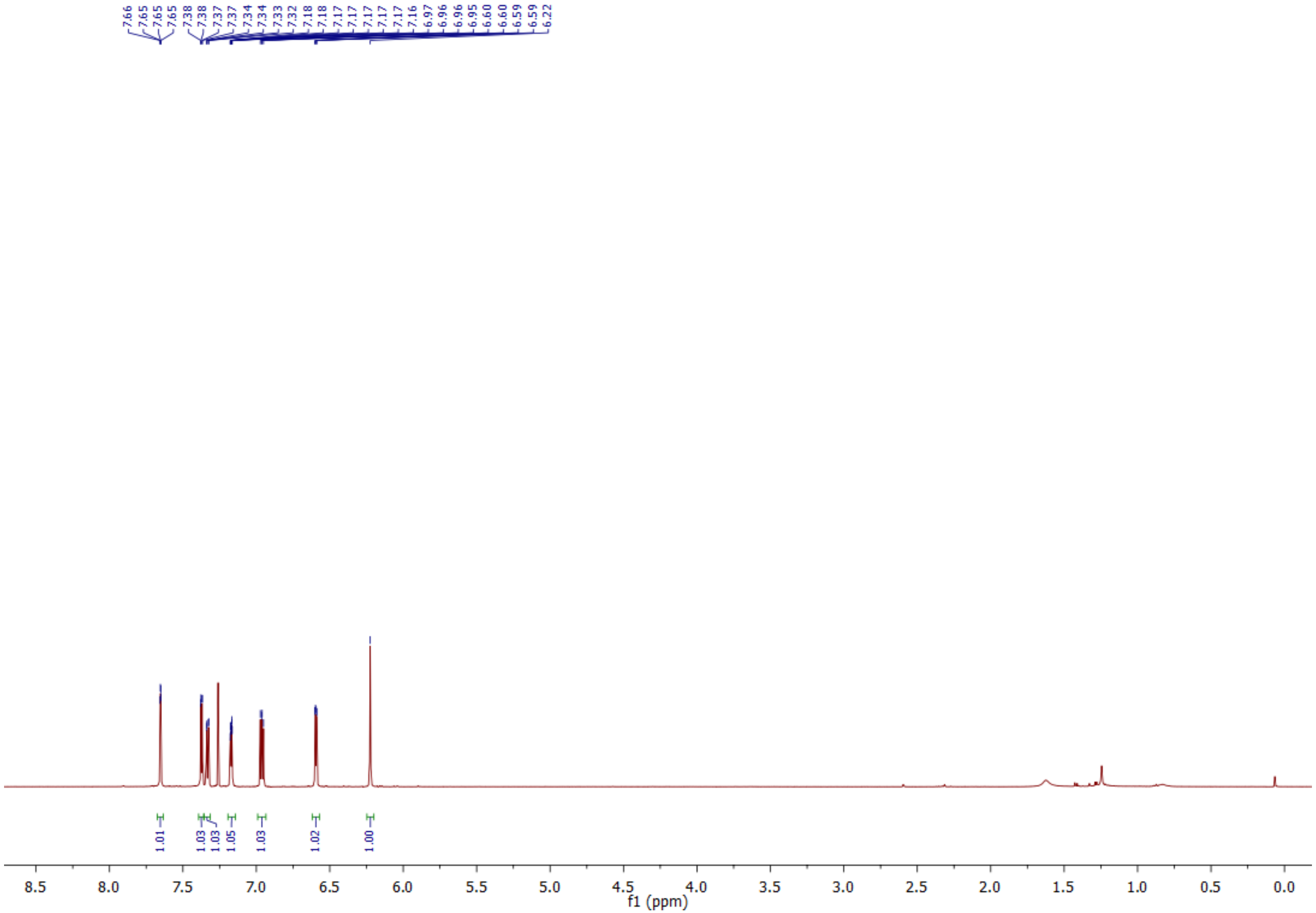

^{13}C NMR ($CDCl_3$, 100 MHz). 1-(Furan-2-yl)-2-(thiophen-2-yl)-2-((trifluoromethyl)thio)ethan-1-one (**5r**)

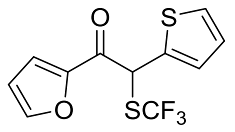

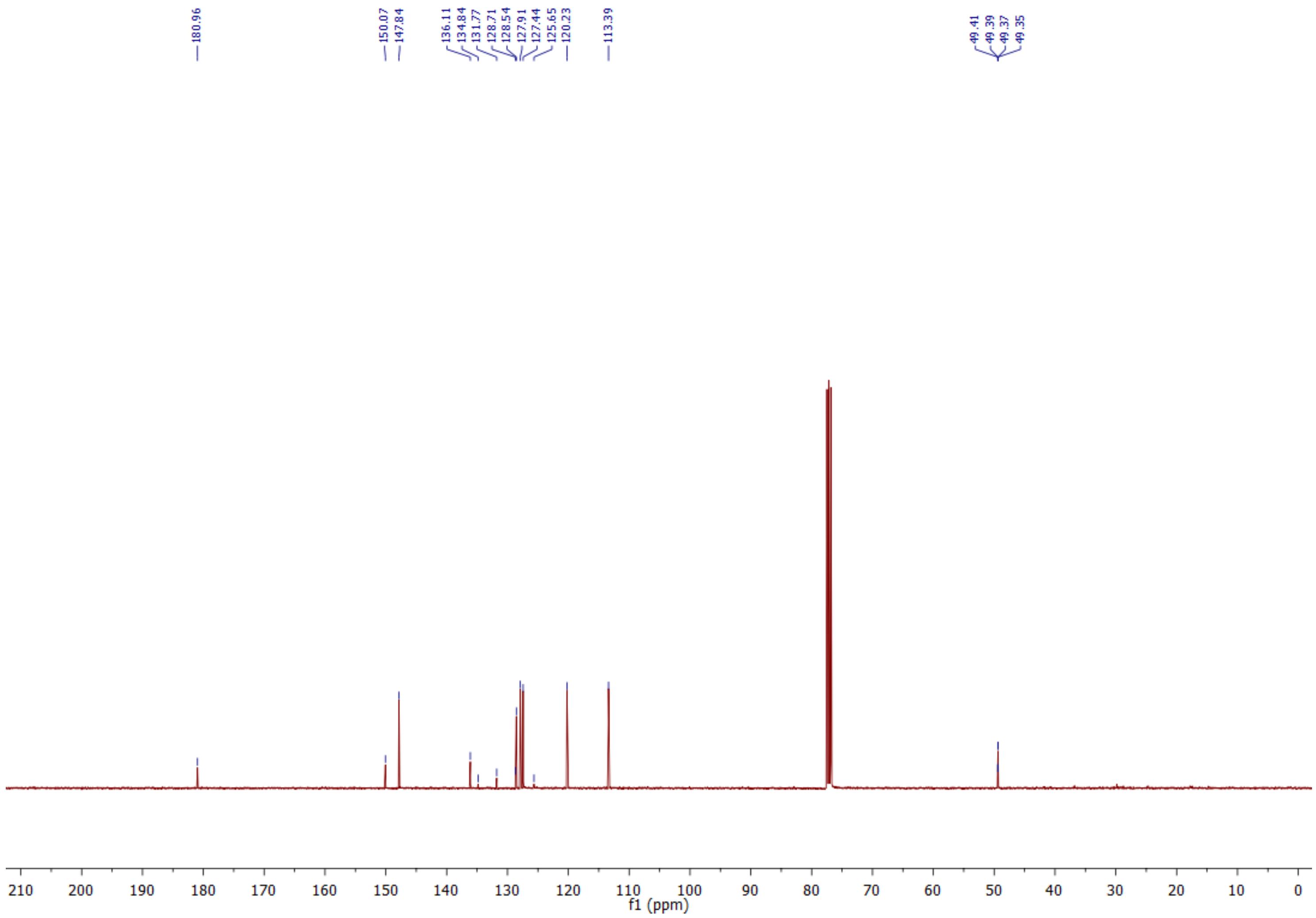

^{19}F NMR ($CDCl_3$, 377 MHz). 1-(Furan-2-yl)-2-(thiophen-2-yl)-2-((trifluoromethyl)thio)ethan-1-one (**5r**)

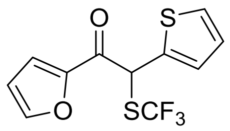

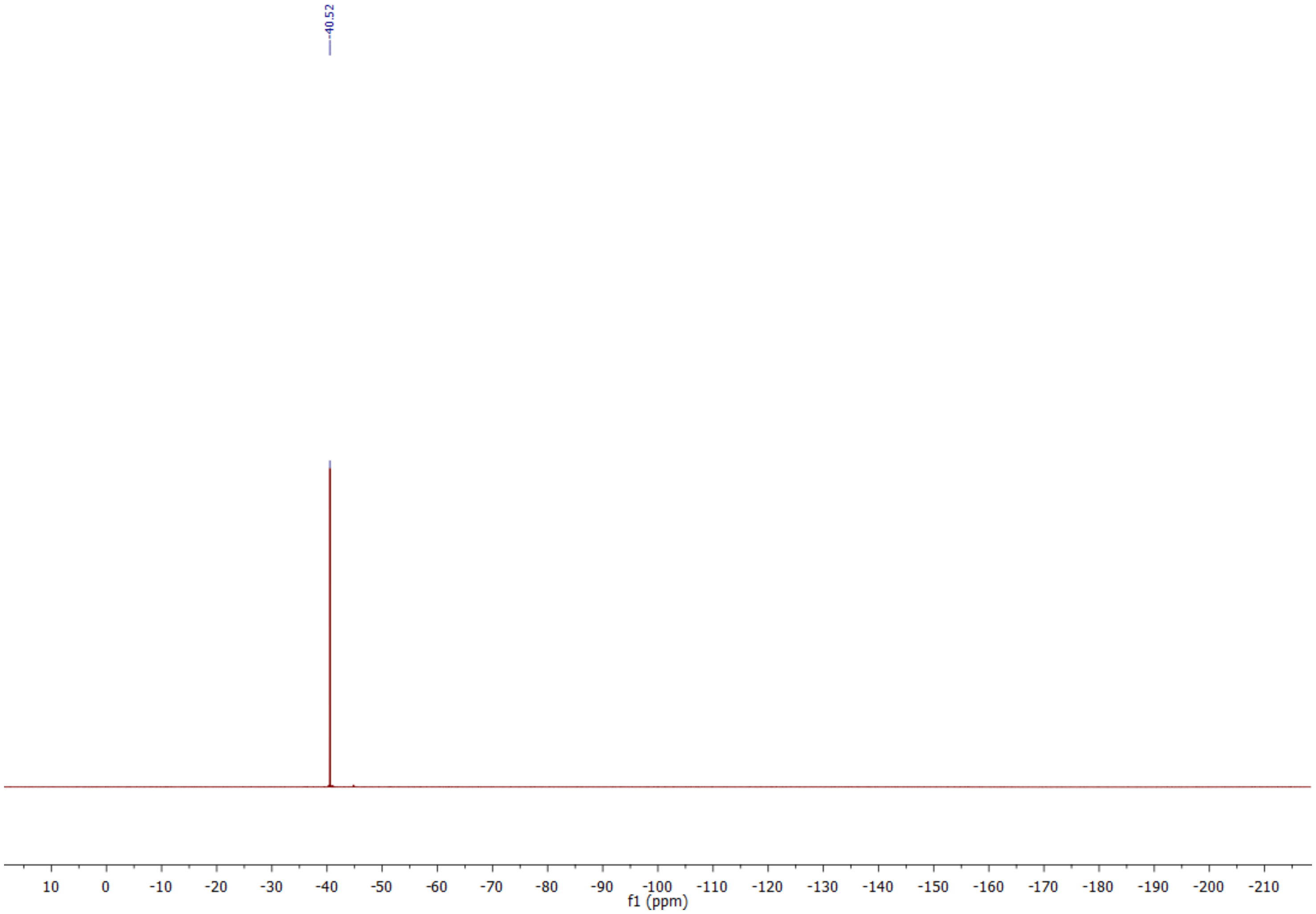

^{1}H NMR ($CDCl_3$, 400 MHz). 2-Fluoro-1-(4-nitrophenyl)-2-phenylethan-1-one (**6**)

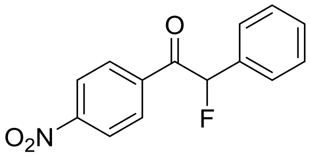

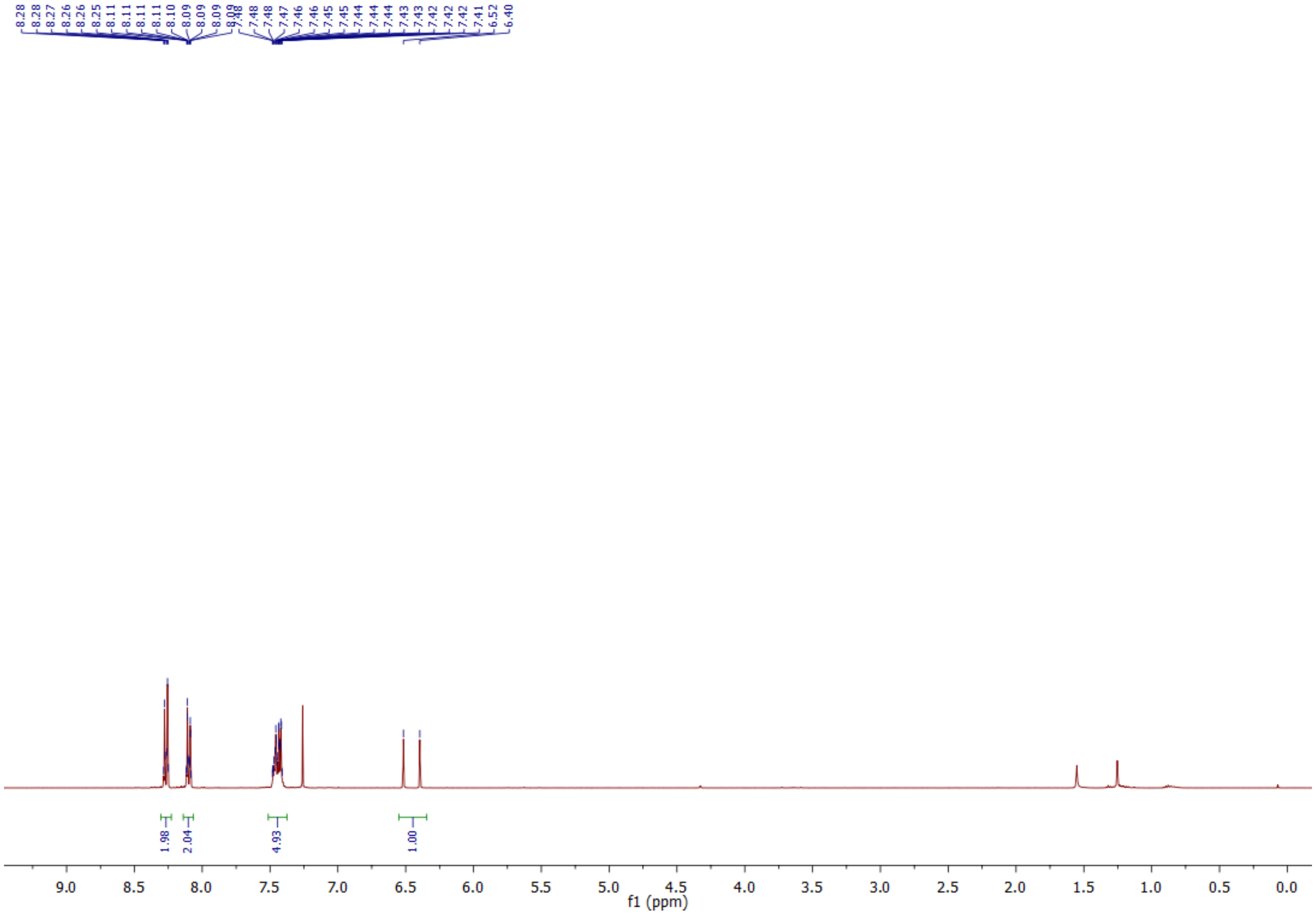

^{13}C NMR ($CDCl_3$, 100 MHz). 2-Fluoro-1-(4-nitrophenyl)-2-phenylethan-1-one (**6**)

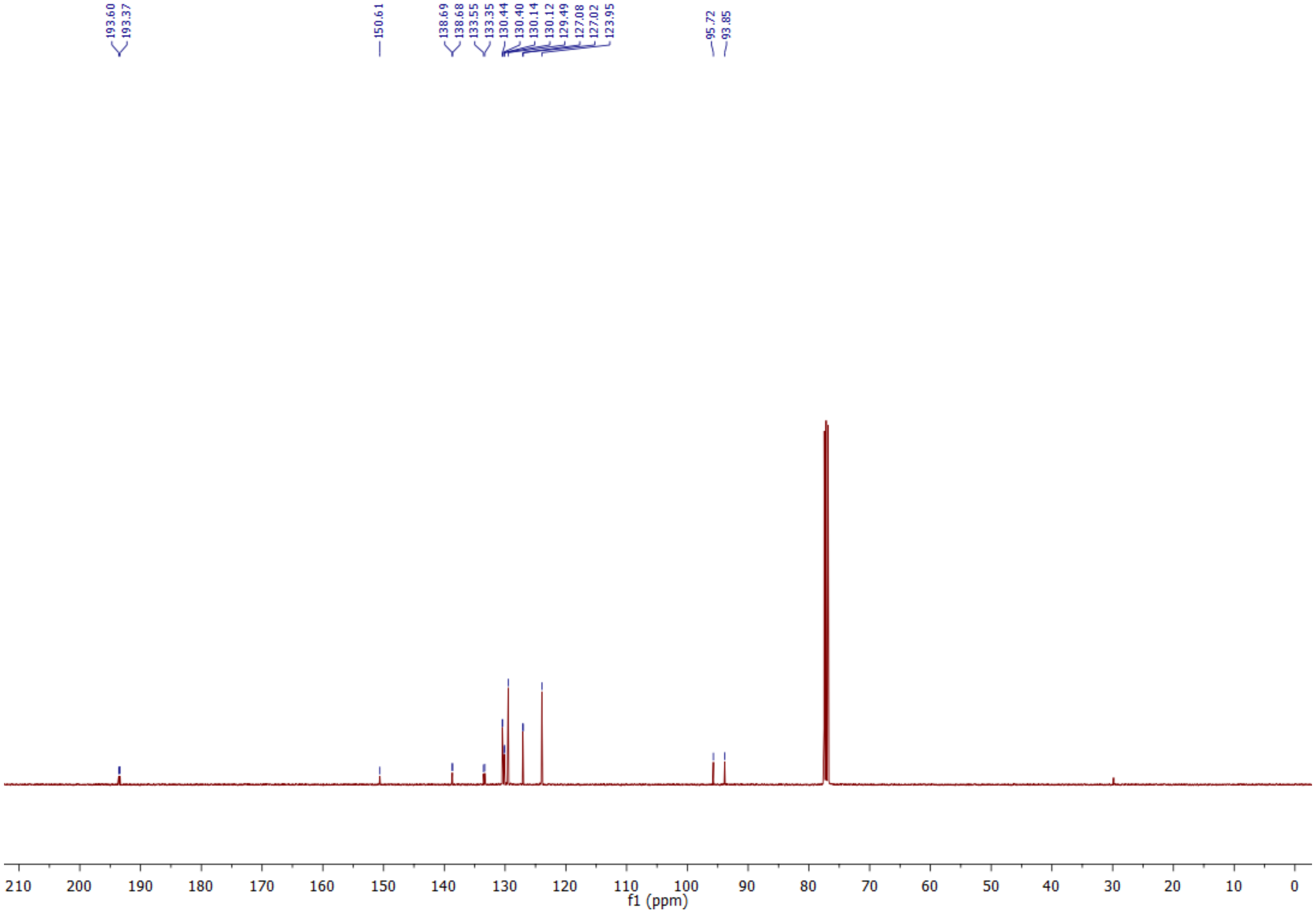

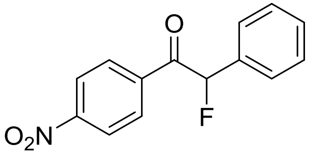

^{19}F NMR ($CDCl_3$, 377 MHz). 2-Fluoro-1-(4-nitrophenyl)-2-phenylethan-1-one (**6**)

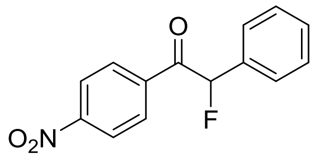

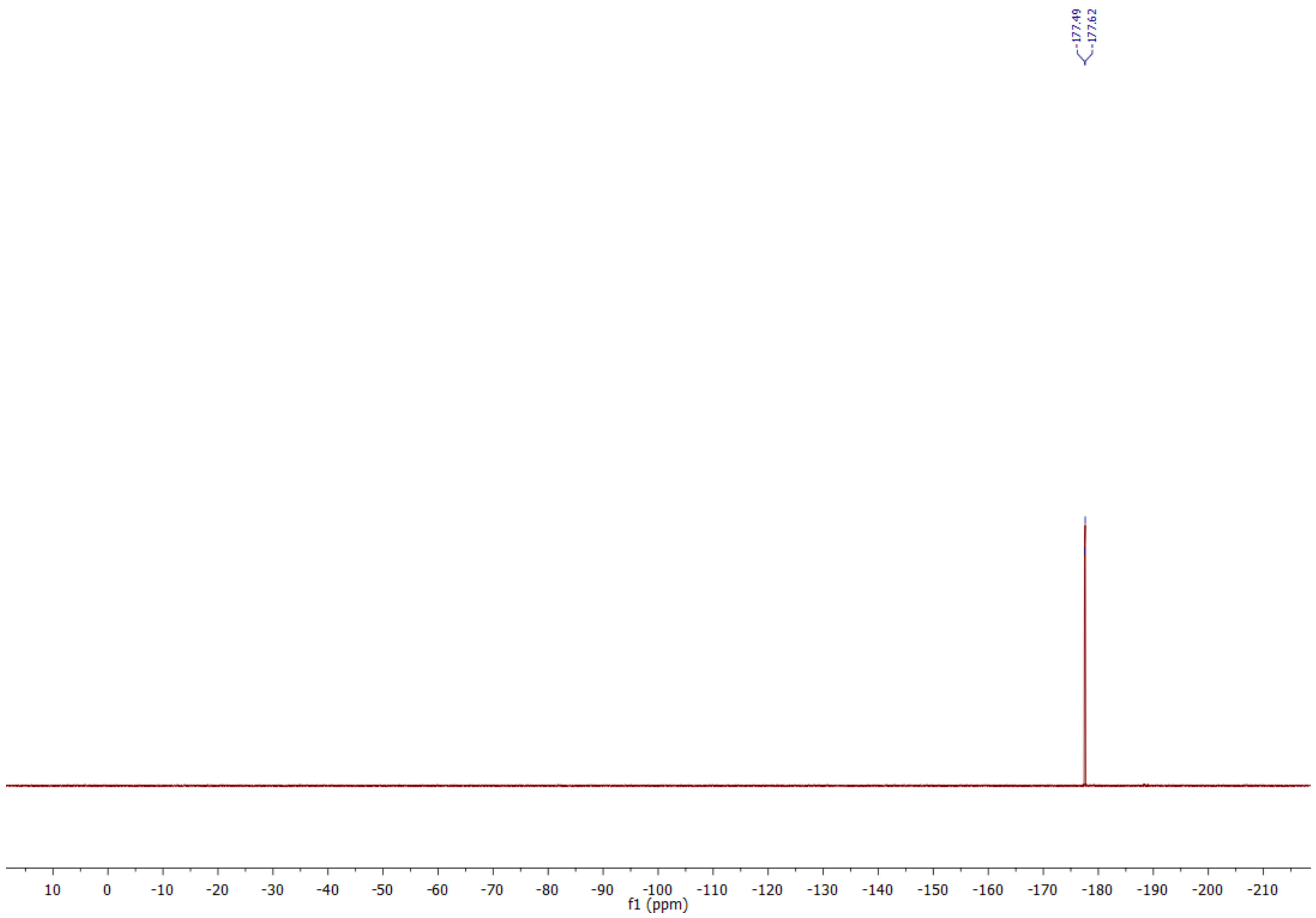

^{1}H NMR ($CDCl_3$, 400 MHz). 3-(4-Bromophenyl)-1-(4-fluorophenyl)-3-hydroxy-2-phenylpropan-1-one (**18**)

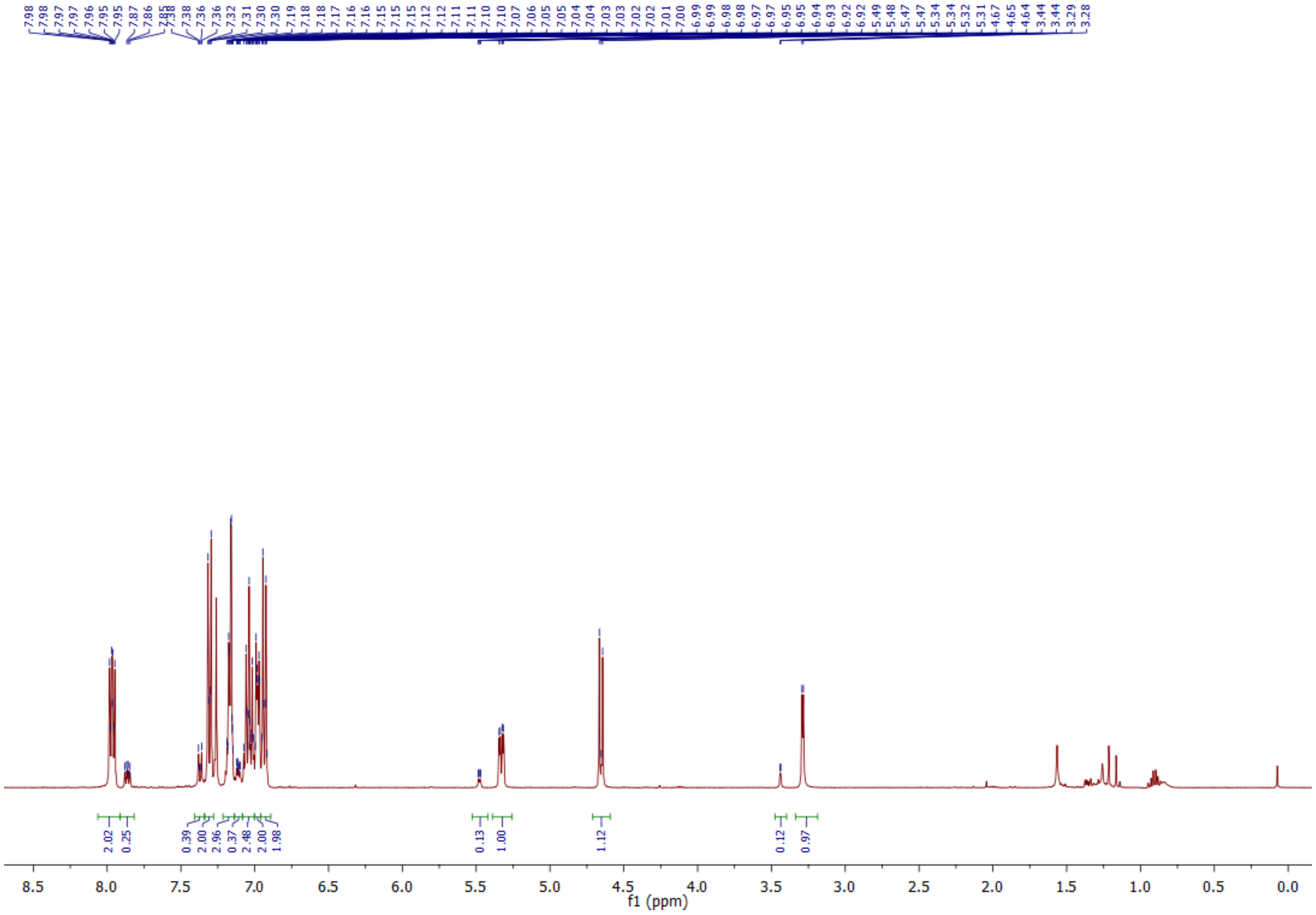

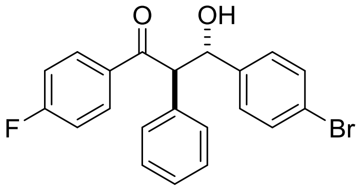

anti

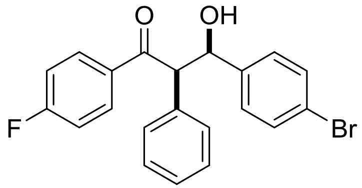

syn

^{13}C NMR (CDCl$_3$, 100 MHz). 3-(4-Bromophenyl)-1-(4-fluorophenyl)-3-hydroxy-2-phenylpropan-1-one (**18**)

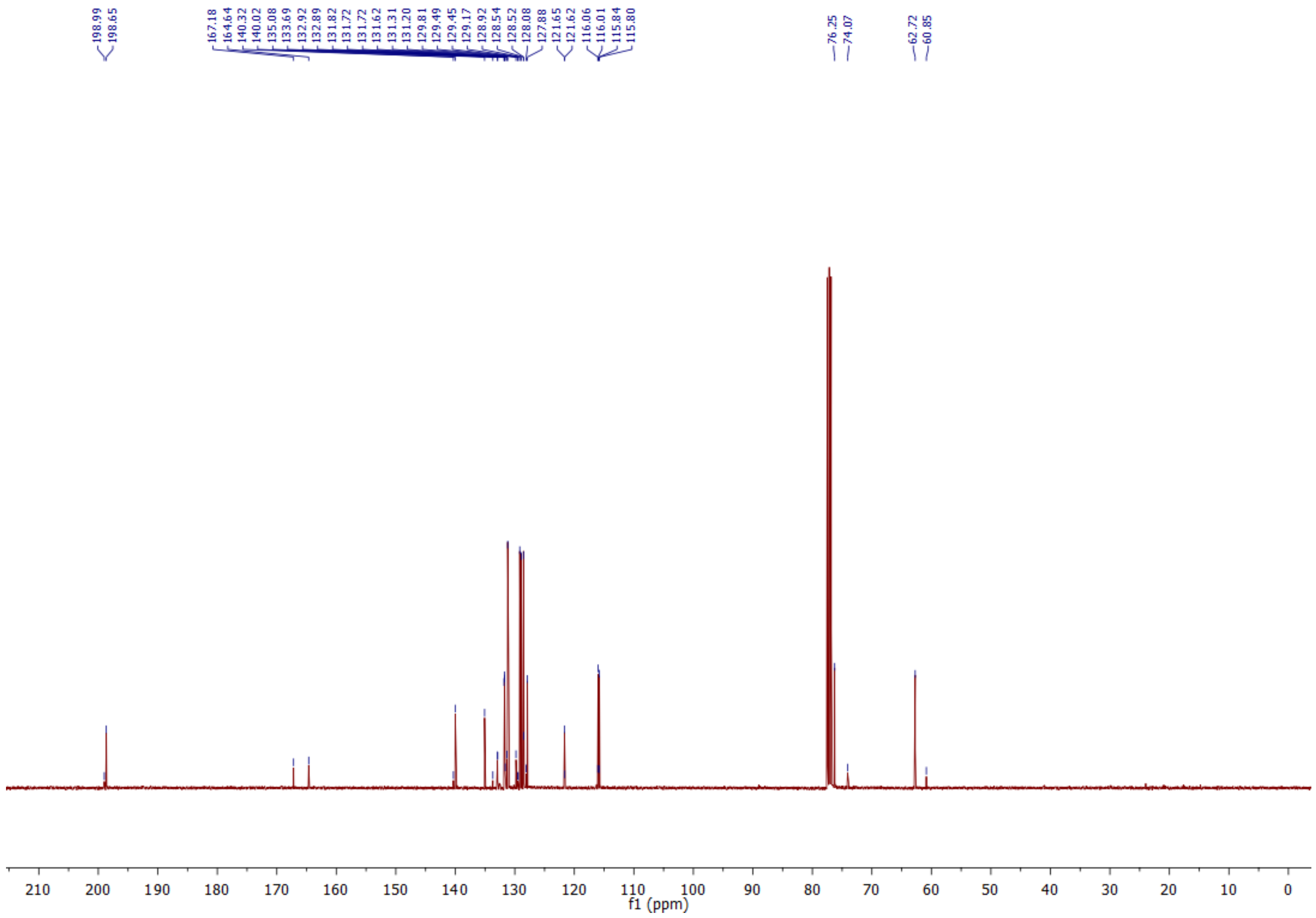

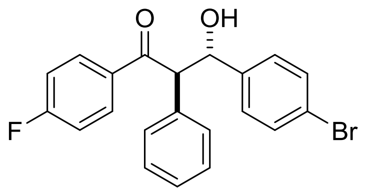

anti

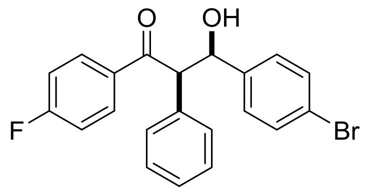

syn

^{19}F NMR ($CDCl_3$, 377 MHz). 3-(4-Bromophenyl)-1-(4-fluorophenyl)-3-hydroxy-2-phenylpropan-1-one (**18**)

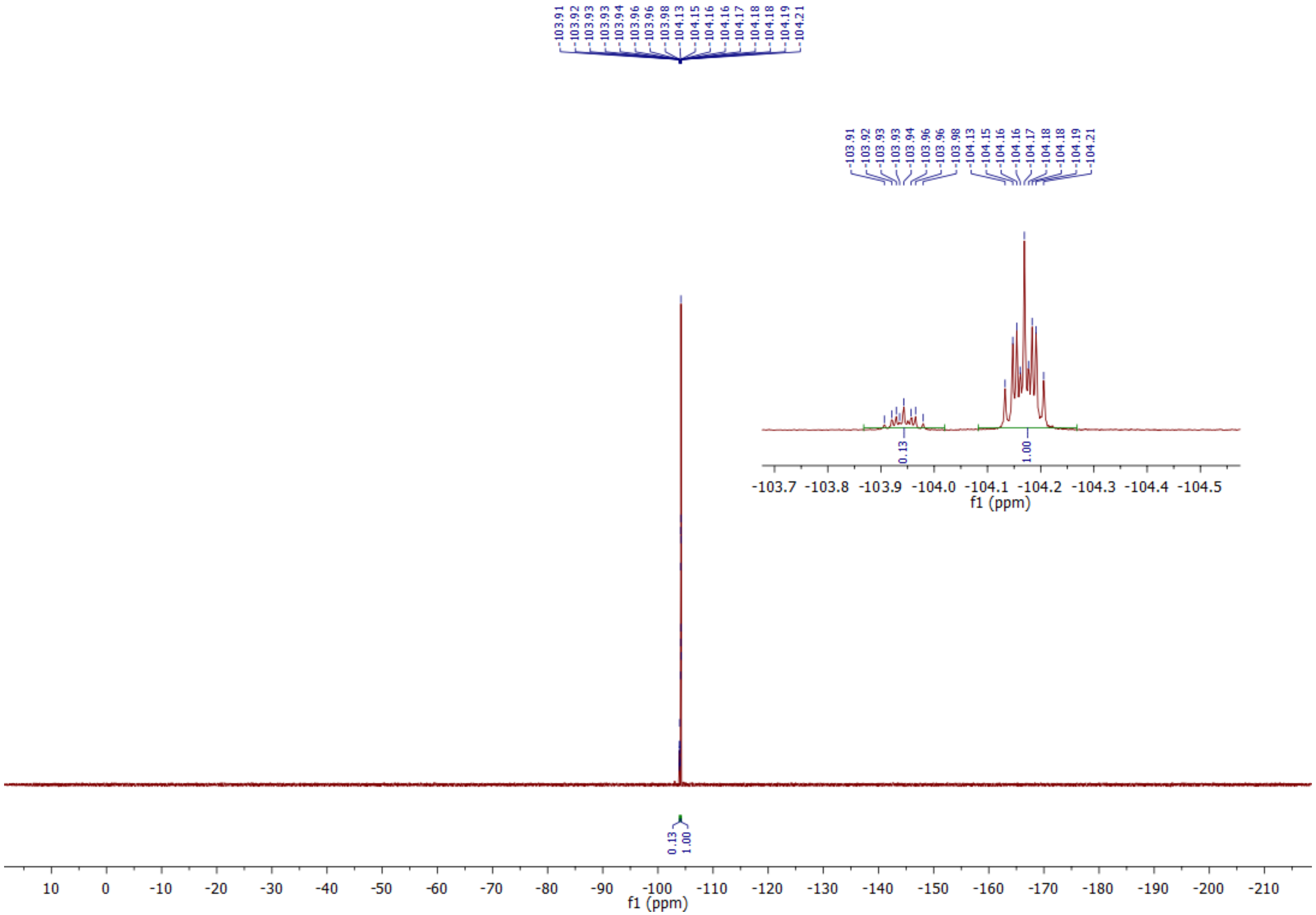

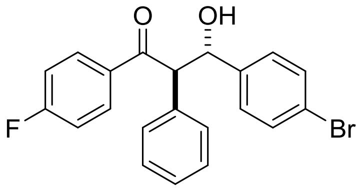

anti

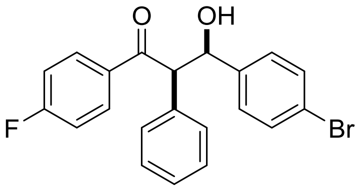

syn

References:

1. A. Das, D. Wang, M.-C. Belhomme and K. J. Szabó, *Org. Lett.*, 2015, **17**, 4754-4757.
2. L. J. Martin, A. L. Marzinzik, S. V. Ley and I. R. Baxendale, *Org. Lett.*, 2011, **13**, 320-323.
3. M. S. Lall, Y. K. Ramtohul, M. N. G. James and J. C. Vederas, *J. Org. Chem.*, 2002, **67**, 1536-1547.
4. N. Döben, H. Yan, M. Kischkewitz, J. Mao and A. Studer, *Org. Lett.*, 2018, **20**, 7933-7936.
5. P. Zhang, M. Li, X.-S. Xue, C. Xu, Q. Zhao, Y. Liu, H. Wang, Y. Guo, L. Lu and Q. Shen, *J. Org. Chem.*, 2016, **81**, 7486-7509.
6. D. Türp, M. Wagner, V. Enkelmann and K. Müllen, *Angew. Chem. Int. Ed.*, 2011, **50**, 4962-4965.
7. Cianci, C., W.; Gerritz, S.; Li, G.; Pearce, B., C.; Pendri, A.; Shi, S.; Zhai, W.; Zhu, S, US pat., US20100380728P, 2010.
8. X. Wang, Y. Zhou, G. Ji, G. Wu, M. Li, Y. Zhang and J. Wang, *Eur. J. Org. Chem.*, 2014, **2014**, 3093-3096.
9. Q. Lefebvre, E. Fava, P. Nikolaienko and M. Rueping, *Chem. Commun.*, 2014, **50**, 6617-6619.
10. Y.-F. Liang, K. Wu, S. Song, X. Li, X. Huang and N. Jiao, *Org. Lett.*, 2015, **17**, 876-879.
11. P. Singh and A. Bhardwaj, *J. Med. Chem.*, 2010, **53**, 3707-3717.
